# Adult body weight trends in 27 urban populations of Brazil from 2006 to 2016: A population-based study

**DOI:** 10.1371/journal.pone.0213254

**Published:** 2019-03-06

**Authors:** Renzo Flores-Ortiz, Deborah Carvalho Malta, Gustavo Velasquez-Melendez

**Affiliations:** Departamento de Enfermagem Materno-Infantil e Saúde Pública, Escola de Enfermagem, Universidade Federal de Minas Gerais, Belo Horizonte, Minas Gerais, Brazil; Weill Cornell Medical College in Qatar, QATAR

## Abstract

**Objective:**

We aimed to estimate trends in population-level adult body weight indicators in the 26 state capitals and the Federal District of Brazil.

**Methods:**

Self-reported weight and height data of 572,437 adults were used to estimate the mean body mass index (BMI), and the prevalence of BMI categories ranging from underweight to morbid obesity, in Brazil’s state capitals and Federal District, from 2006 to 2016, by sex. All estimates were standardized by age.

**Results:**

From 2006 to 2016, the main findings showed that: (i) the overall mean BMI increased from 25.4 kg/m^2^ to 26.3 kg/m^2^ in men, and from 24.5 kg/m^2^ to 25.8 kg/m^2^ in women; (ii) the overall prevalence of overweight increased from 48.1% to 57.5% in men, and from 37.8% to 48.2% in women; (iii) the overall prevalence of obesity increased from 11.7% to 18.1% in men, and from 12.1% to 18.8% in women; (iv) in general, the largest increases in overweight and obesity prevalence were found in state capitals located in the north, northeast, and central-west regions of Brazil; (v) the prevalence of severe obesity surpassed the prevalence of underweight in 22 and 9 state capitals among men and women, respectively; and (vi) the mean BMI trend was stable only in Vitória state capital in men.

**Conclusions:**

The policies for preventing and treating obesity in Brazil over the past years were not able to halt the increase in obesity prevalence either in the state capitals or the Federal District. Thus, a revision of policies is warranted. Furthermore, although policies are necessary in all state capitals, our results suggest that policies are especially necessary in the north, northeast, and central-west regions’ state capitals, where, in general, the largest increases in overweight and obesity prevalence were experienced.

## Introduction

Body weight is an important health-related characteristic. Overweight (levels of weight higher than what is considered healthy for a given height) can be a potential risk factor for non-communicable diseases (NCDs) such as diabetes, hypertension, stroke, and osteoarthritis [1]. Underweight (levels of weight lower than what is considered healthy for a given height) can be a potential risk factor for NCDs such as dementia, osteoporosis, infertility, and iron-deficiency anemia [2]. In addition, both overweight and underweight are associated with disabilities, premature death, and various types of cancers [1,2].

Overweight and particularly obesity are among the most alarming public health problems being faced worldwide today. It was estimated that in 2015, overweight contributed to 4 million deaths and 120 million disability-adjusted life years globally [1]. Furthermore, it was estimated that in 2012, obesity had a global economic impact of US$ 2 trillion, which accounted for the loss of productive life years, the direct costs to health-care systems, and the investments required to mitigate the impacts of obesity [3]. Despite overweight and obesity being the most worrisome weight status in the world today, it is important to highlight that underweight still remains a major public health problem in some low and middle income countries [2,4].

Given the severity of the health and economic burdens associated with body weight, the monitoring of population-level body weight indicators is important to support the planning of public health responses [1,5]. This is especially applicable in urban areas and in low and middle income countries, where the highest levels of overweight and obesity prevalence are being observed [5–7]. Thus, the purpose of this study was to estimate trends in body weight indicators in a representative sample of adults in the state capitals and Federal District of Brazil, which may be considered the main urban areas of this important middle income country. It should be mentioned that the monitoring of body weight indicators in Brazil’s state capitals and Federal District is of interest not only to Brazil, but it may also be of interest to other middle income countries in less advanced stages of the nutrition transition, as they may experience similar trends in the future.

Previous studies that estimated trends in adult body weight indicators in Brazil’s state capitals and Federal District, provided, in general, crude estimates of overweight and obesity prevalence until 2013 [8,9]. Our study proposed to extend these previous studies by providing age-standardized estimates of mean body mass index (BMI) and prevalence of BMI categories ranging from underweight to morbid obesity (i.e., covering the entire range of the BMI distribution) up to 2016.

## Materials and methods

### Data source, collection, sampling, and ethical aspects

**Fig 1:**
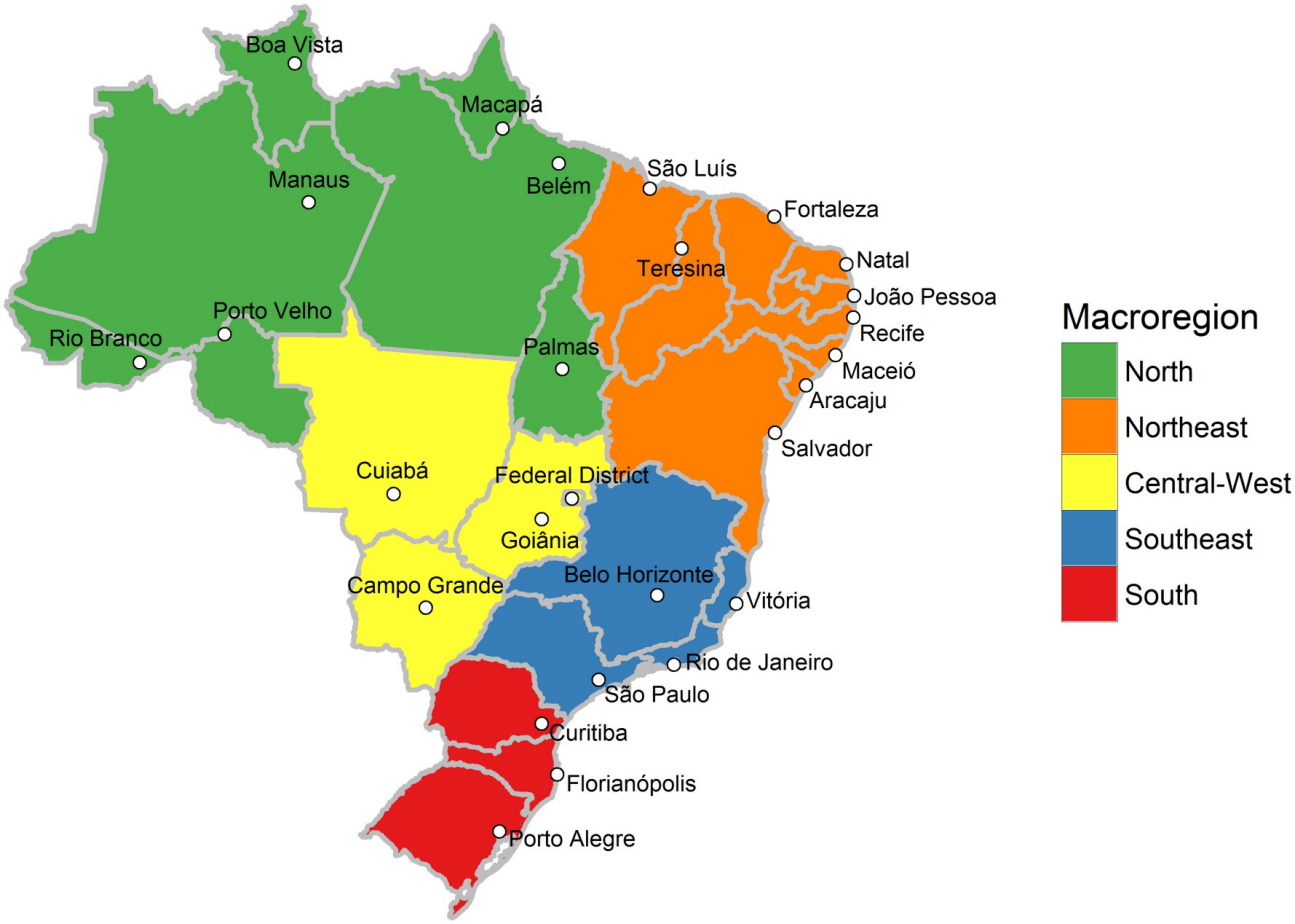
State capitals, Federal District, and macro-regions of Brazil. The territory of Brazil is composed of 26 states and a Federal District. The grey lines depict the states’ limits. The white dots indicate the location of the states’ capitals and the Federal District. The colored areas represent the five macro-regions of Brazil.

We analyzed data from the Telephone Surveillance of Risk and Protective Factors for Chronic Diseases (VIGITEL, from its acronym in Portuguese). This is a telephone health survey designed to provide a representative sample of the 26 state capitals and the Federal District of Brazil adult population with at least one residential fixed telephone line (Fig 1) [10]. For ease of communication, the Federal District will henceforth be regarded as one of the state capitals, making a total of 27 state capitals.

The VIGITEL survey is conducted annually since 2006 and we analyzed the data from the surveys of 2006 to 2016, which were the survey-years whose data were available for analysis at the time of the development of this study.

The VIGITEL survey conducts approximately 2,000 interviews per year in each state capital, totaling approximately 54,000 interviews per survey-year. The interviews are conducted by a team of approximately 40 interviewers who administer a structured questionnaire evaluating sociodemographic, anthropometric, lifestyle, dietary, NCDs, and health services utilization characteristics.

The VIGITEL sampling procedure is performed in two stages where in the first stage are selected telephone lines, and in the second stage is selected an adult from the residence of the selected telephone line. An adult is defined as a person aged 18 years or older.

The VIGITEL survey is approved by the National Ethics Committee for Research on Human Beings of the Brazilian Ministry of Health (project protocol number: 16202813.2.0000.0008 [10]), and informed consent was obtained orally from all participants prior to the questionnaire administration.

### Data analysis

Initially, the BMI, defined as weight in kilograms divided by the square of the height in meters, was calculated for all participants. For the participants who did not know or preferred not to supply their weight or height information, the BMI was calculated using imputed values provided by the VIGITEL survey [10].

Individual-level continuous BMI values were then used to estimate the mean BMI and the prevalence of BMI categories, from 2006 to 2016, by sex, for each state capital and for the state capitals overall. The BMI categories included: underweight (BMI < 18.5 kg/m^2^), normal weight (18.5 kg/m^2^ ≤ BMI < 25 kg/m^2^), pre-obesity (25 kg/m^2^ ≤ BMI < 30 kg/m^2^), moderate obesity (30 kg/m^2^ ≤ BMI < 35 kg/m^2^), severe obesity (35 kg/m^2^ ≤ BMI < 40 kg/m^2^), morbid obesity (BMI ≥ 40 kg/m^2^), overweight (BMI ≥ 25 kg/m^2^), and obesity (BMI ≥ 30 kg/m^2^). These BMI categories were defined according to World Health Organization (WHO) guidelines on BMI classification [11].

The mean BMI and prevalence of BMI categories were estimated using the direct age-standardization method [12]. The standard population used to apply this method was the state capitals’ total population assessed in the 2010 Brazilian Census [13]. Confidence intervals (CI) with 95% confidence level were estimated for all indicators. Descriptive statistics (means or proportions, and standard deviations (SD)) were estimated for age, sex, years of schooling, marital status, and occupational status. The estimation of body weight indicators, CI, and descriptive statistics considered the complex survey sampling design and post-stratification weights, which allowed correcting for deviances in the probability of selection of the study participants: adults living in households with more than one telephone line had a higher probability of being selected, and adults living in households with other adults had a lower probability of being selected [10]. Lastly, a linear regression model of the mean BMI against year was estimated to obtain the mean rate of change in the mean BMI throughout the study period. This linear regression model was estimated using the Prais-Winsten method [14], which allowed taking into account the temporal correlation present in the mean BMI data.

All analyses described were conducted using the R statistical software version 3.3.3 (The R Foundation for Statistical Computing, Vienna, Austria; http://www.r-project.org).

## Results

### Study sample description

A total of 572,437 adults were interviewed in the VIGITEL surveys conducted from 2006 to 2016. The survey-years with the most and least participants were 2006 (54,369) and 2014 (40,853), respectively. The state capitals with the most and least participants were Belo Horizonte (21,363) and Macapá (21,040), respectively. On average, the participants were 41 years old (SD = 16.4 years), had 10 years of schooling (SD = 4.8 years), the majority were women (53.9%), married (42.4%), and had worked in the three months prior to the survey interview (64.7%).

### Mean BMI trends

**Fig 2:**
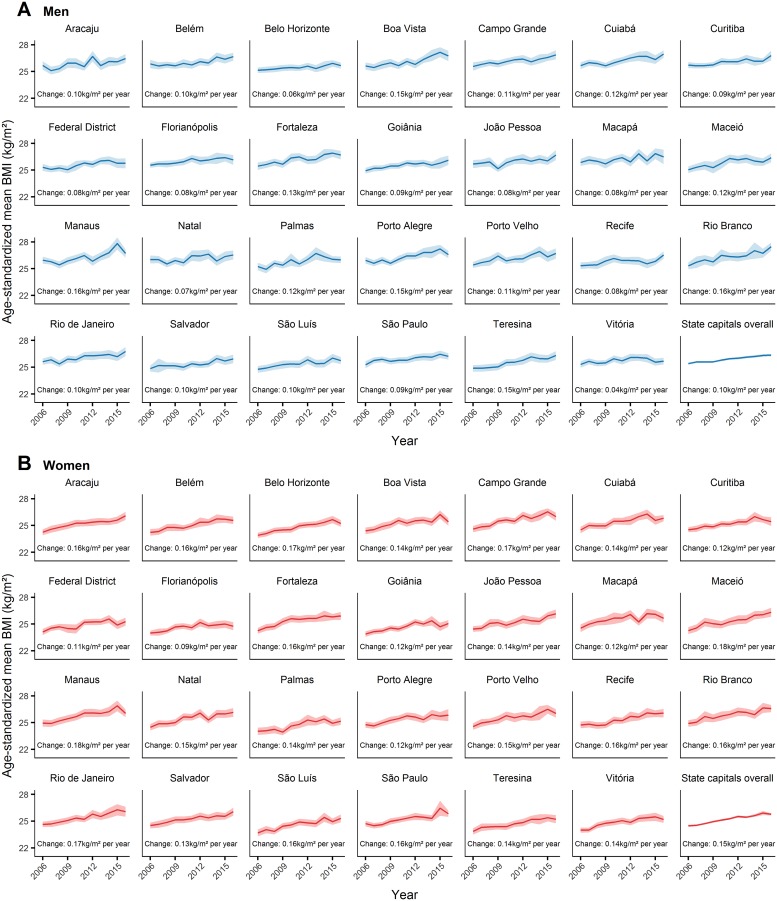
Trends in age-standardized mean BMI in Brazil’s state capitals, from 2006 to 2016, among men (A) and women (B). The solid line represents the mean BMI and the shaded area the 95% confidence interval. For numerical results see S1 and S2 Tables. BMI = body mass index.

From 2006 to 2016, a trend of increase in the mean BMI was found in all state capitals in both sexes (Fig 2; for numerical results see S1 and S2 Tables). In men, the mean rate of increase in the mean BMI was highest in Manaus and Rio Branco (0.16 kg/m^2^ per year), and lowest in Vitória (0.04 kg/m^2^ per year). In women, the mean rate of increase in the mean BMI was highest in Maceió and Manaus (0.18 kg/m^2^ per year), and lowest in Florianópolis (0.09 kg/m^2^ per year). The mean rate of increase in the overall mean BMI was highest in women (0.15 kg/m^2^ per year) compared to men (0.10 kg/m^2^ per year).

**Fig 3:**
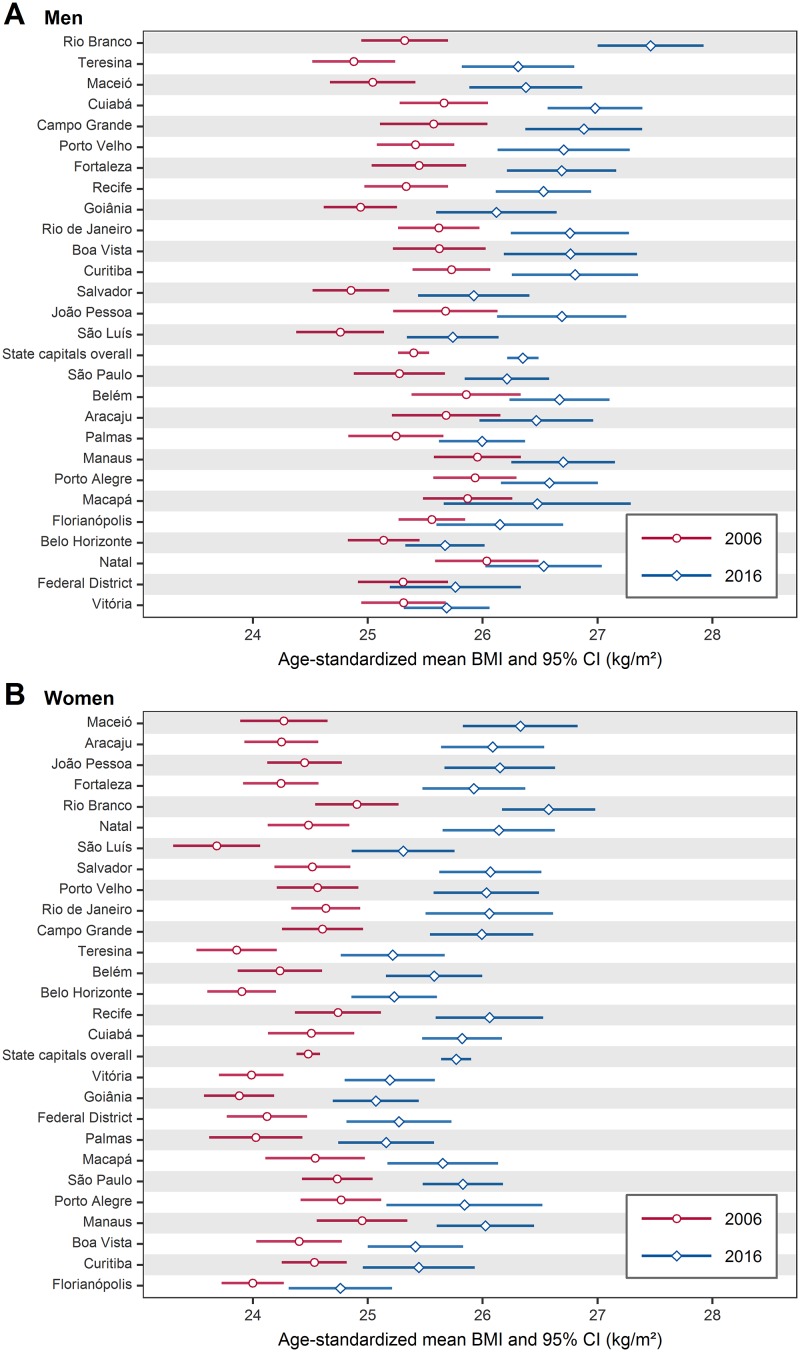
Age-standardized mean BMI in Brazil’s state capitals, in 2006 and 2016, among men (A) and women (B). The state capitals are ordered from bottom to top by increase in mean BMI. For numerical results see S1 and S2 Tables. BMI = body mass index; CI = confidence interval.

From 2006 to 2016, the mean BMI in men (Fig 3A) increased more in Rio Branco (increased from 25.3 kg/m^2^ to 27.5 kg/m^2^, a relative increase of 8.7%), and less in Vitória (increased from 25.3 kg/m^2^ to 25.7 kg/m^2^, a relative increase of 1.6%). The mean BMI in women (Fig 3B) increased more in Maceió (increased from 24.3 kg/m^2^ to 26.3 kg/m^2^, a relative increase of 8.2%), and less in Florianópolis (increased from 24.0 kg/m^2^ to 24.8 kg/m^2^, a relative increase of 3.3%). The overall mean BMI increased from 25.4 kg/m^2^ to 26.3 kg/m^2^ in men (a relative increase of 3.5%), and from 24.5 kg/m^2^ to 25.8 kg/m^2^ in women (a relative increase of 5.3%).

### BMI category prevalence trends

**Fig 4:**
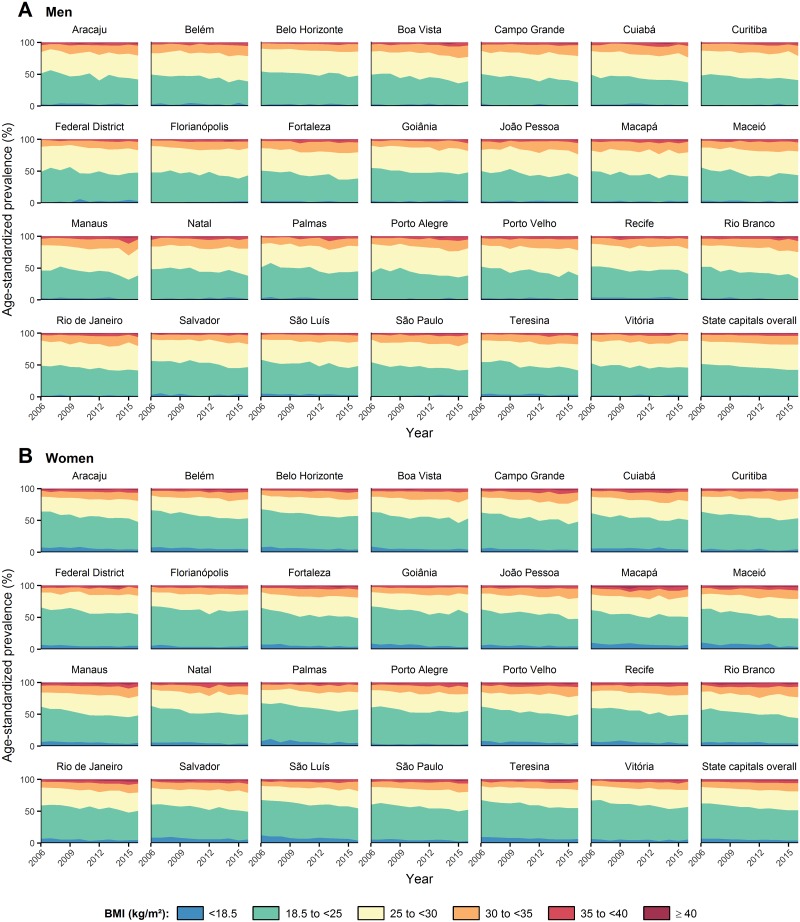
Trends in age-standardized prevalence of underweight (BMI < 18.5 kg/m^2^), normal weight (18.5 kg/m^2^ ≤ BMI < 25 kg/m^2^), pre-obesity (25 kg/m^2^ ≤ BMI < 30 kg/m^2^), moderate obesity (30 kg/m^2^ ≤ BMI < 35 kg/m^2^), severe obesity (35 kg/m^2^ ≤ BMI < 40 kg/m^2^), and morbid obesity (BMI ≥ 40 kg/m^2^) in Brazil’s state capitals, from 2006 to 2016, among men (A) and women (B). For numerical results see S3–S14 Tables. BMI = body mass index.

Fig 4 shows the prevalence of underweight, normal weight, pre-obesity, moderate obesity, severe obesity, and morbid obesity in Brazil’s state capitals, from 2006 to 2016, among men and women (for numerical results see S3–S14 Tables).

The prevalence of underweight (BMI < 18.5 kg/m^2^) in men decreased in all state capitals except Aracaju, Belo Horizonte, Curitiba, Federal District, Florianópolis, Macapá, Natal, Porto Alegre, and Vitória. The prevalence of underweight in women decreased in all state capitals except Curitiba and Florianópolis. The overall prevalence of underweight decreased from 2.3% to 2.0% in men (a relative decrease of 13.0%), and from 6.7% to 4.2% in women (a relative decrease of 37.3%).

The prevalence of normal weight (18.5 kg/m^2^ ≤ BMI < 25 kg/m^2^) decreased in all state capitals in both sexes. The overall prevalence of normal weight decreased from 49.6% to 40.5% in men (a relative decrease of 18.3%), and from 55.6% to 47.6% in women (a relative decrease of 14.4%).

The prevalence of pre-obesity (25 kg/m^2^ ≤ BMI < 30 kg/m^2^) in men increased in all state capitals except Curitiba, Federal District, Goiânia, Porto Alegre, and Rio de Janeiro. The prevalence of pre-obesity in women increased in all state capitals except Porto Alegre. The overall prevalence of pre-obesity increased from 36.5% to 39.4% in men (a relative increase of 7.9%), and from 25.7% to 29.4% in women (a relative increase of 14.4%).

The prevalence of moderate obesity (30 kg/m^2^ ≤ BMI < 35 kg/m^2^) increased in all state capitals in both sexes. The overall prevalence of moderate obesity increased from 9.1% to 13.8% in men (a relative increase of 51.6%), and from 8.3% to 12.8% in women (a relative increase of 54.2%).

The prevalence of severe obesity (35 kg/m^2^ ≤ BMI < 40 kg/m^2^) in men increased in all state capitals except Aracaju, Campo Grande, Macapá, Manaus, Natal, and Vitória. The prevalence of severe obesity in women increased in all state capitals except Recife. The overall prevalence of severe obesity increased from 1.7% to 3.1% in men (a relative increase of 82.4%), and from 2.5% to 4.0% in women (a relative increase of 60.0%).

The prevalence of morbid obesity (BMI ≥ 40 kg/m^2^) in men increased in all state capitals except Belém, Belo Horizonte, Cuiabá, Porto Alegre, and São Paulo. The prevalence of morbid obesity in women increased in all state capitals except Boa Vista, Curitiba, Federal District, Florianópolis, Manaus, São Luís, and Vitória. The overall prevalence of morbid obesity increased from 0.9% to 1.2% in men (a relative increase of 33.3%), and from 1.2% to 2.0% in women (a relative increase of 66.7%).

**Fig 5:**
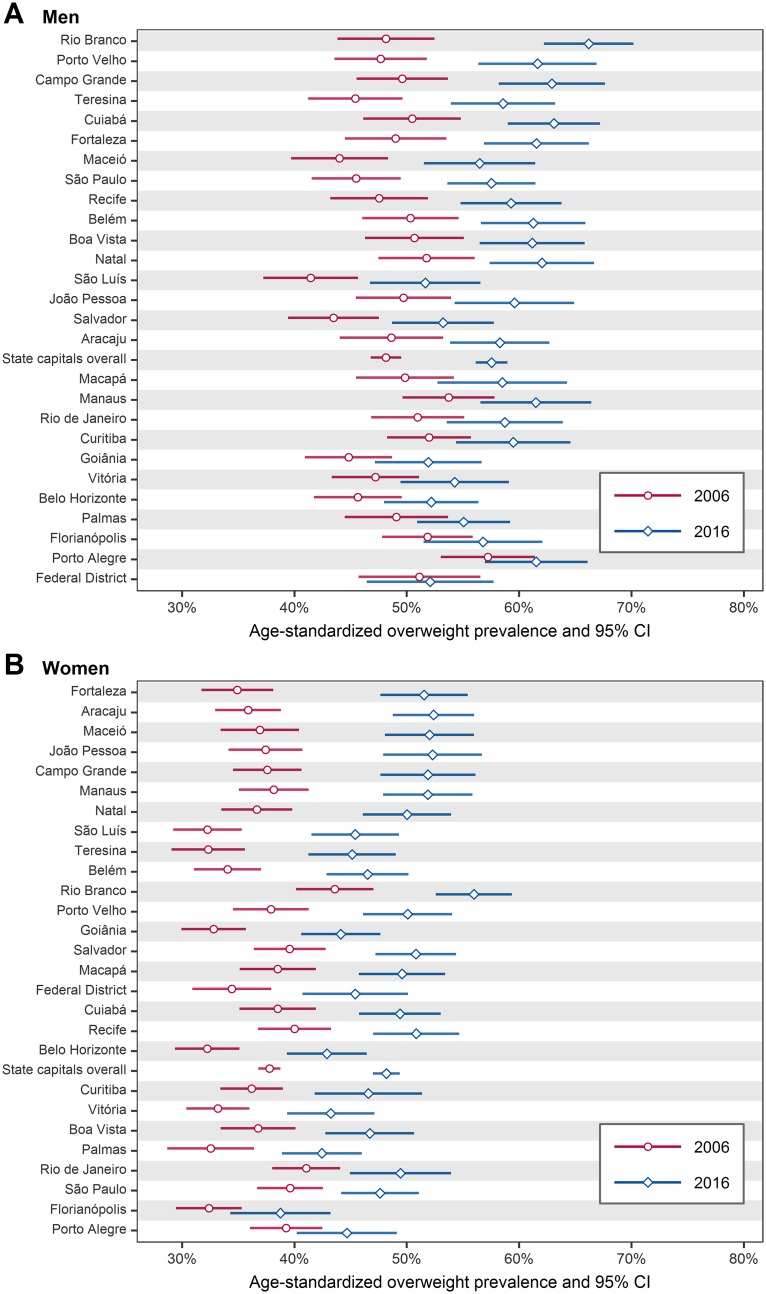
Age-standardized prevalence of overweight (BMI ≥ 25 kg/m^2^) in Brazil’s state capitals, in 2006 and 2016, among men (A) and women (B). The state capitals are ordered from bottom to top by increase in prevalence of overweight. For numerical results see S15 and S16 Tables. BMI = body mass index; CI = confidence interval.

The prevalence of overweight (BMI ≥ 25 kg/m^2^) in 2006 and 2016 are shown in Fig 5, where it can be noted an increase in all state capitals in both sexes (for numerical results see S15 and S16 Tables). The overall prevalence of overweight increased from 48.1% to 57.5% in men (a relative increase of 19.5%), and from 37.8% to 48.2% in women (a relative increase of 27.5%).

**Fig 6:**
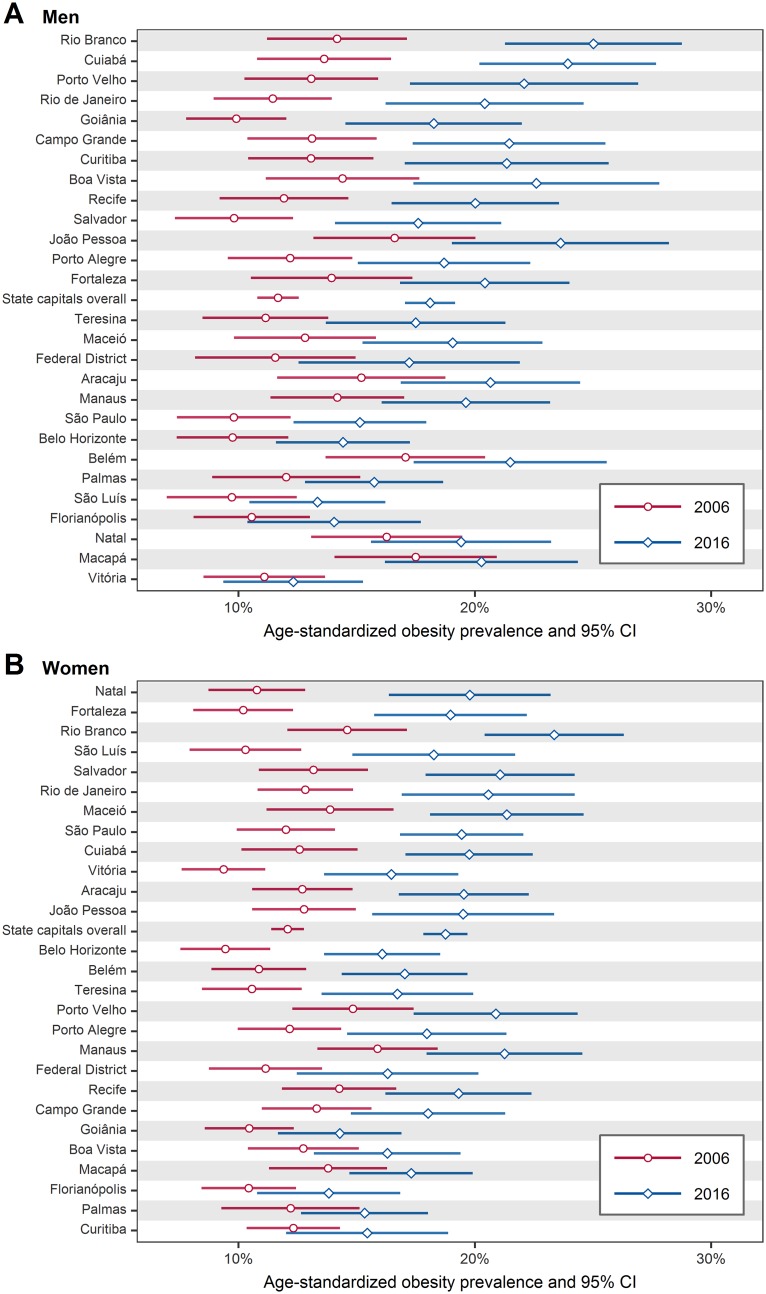
Age-standardized prevalence of obesity (BMI ≥ 30 kg/m^2^) in Brazil’s state capitals, in 2006 and 2016, among men (A) and women (B). The state capitals are ordered from bottom to top by increase in prevalence of obesity. For numerical results see S17 and S18 Tables. BMI = body mass index; CI = confidence interval.

The prevalence of obesity (BMI ≥ 30 kg/m^2^) in 2006 and 2016 are shown in Fig 6, where it can be noted an increase in all state capitals in both sexes (for numerical results see S17 and S18 Tables). The overall prevalence of obesity increased from 11.7% to 18.1% in men (a relative increase of 54.7%), and from 12.1% to 18.8% in women (a relative increase of 55.4%).

### Age-standardized distribution of BMI

**Fig 7:**
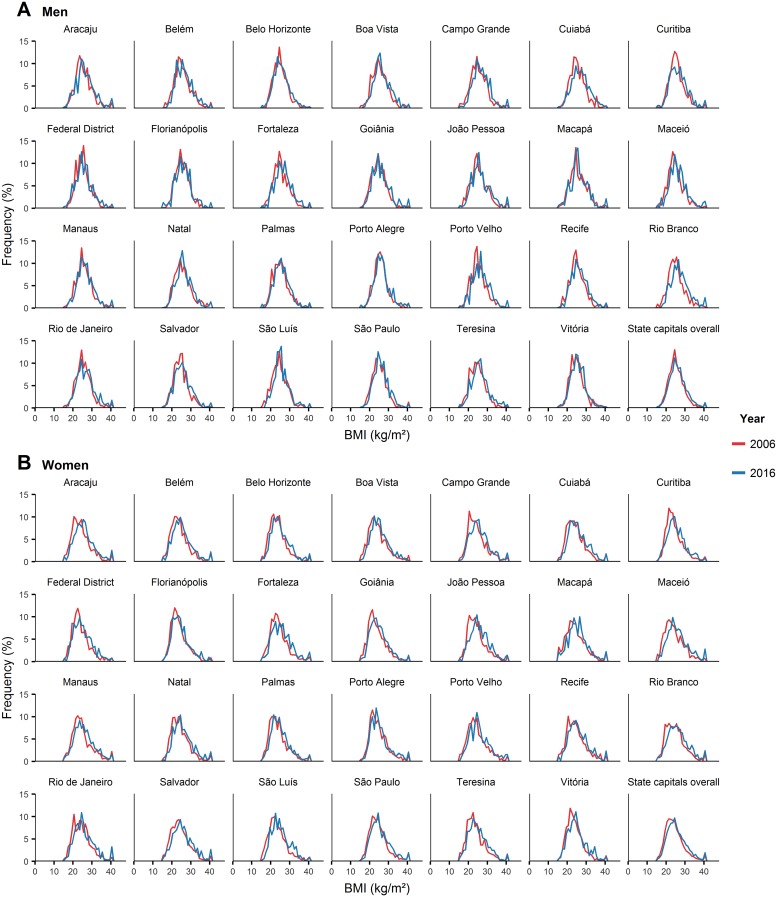
Age-standardized distribution of BMI in Brazil’s state capitals, in 2006 and 2016, among men (A) and women (B). BMI = body mass index.

Lastly, Fig 7 shows that from 2006 to 2016, the age-standardized distribution of BMI shifted towards the higher levels of the BMI scale in all state capitals in both sexes. This result summarizes and corroborates the variations from 2006 to 2016 in mean BMI (Figs 2 and 3) and BMI category prevalence (Figs 4–6).

## Discussion

In this study, we analyzed trends in body weight indicators of great clinical and public health importance. To our knowledge, this was the first study to estimate trends in body weight indicators that measured the entire range of the BMI distribution in Brazil’s state capitals. Furthermore, although trends in overweight and obesity prevalence had been estimated previously [8,9], this is the first time that trends in these indicators were estimated standardized by age and estimated up to 2016. Thus, the analysis of body weight trends presented in this study is not only more complete, but also more methodologically robust and updated.

Among novel results, we highlight the surpassing of severe obesity prevalence over underweight prevalence in 22 and 9 state capitals among men and women, respectively. This result draws attention because it evidences an advanced stage of the nutrition transition. We also highlight that despite the trend of increase in the mean BMI in all state capitals (Fig 2), in Vitória among men, the trend may be considered approximately stable (0.04 kg/m^2^ per year). A possible contributing factor to this result is the fact that over the past years, physical activity levels among men were, in general, higher in Vitória compared to the other state capitals: from 2012 to 2016, the proportion of adult men who engaged in moderate physical activity for at least 150 minutes per week, or vigorous physical activity for at least 75 minutes per week, was, on average, 51.3% in Vitória, while overall in the other state capitals was, on average, 45.1% [15–19].

It should also be highlighted that if we consider the mean heights in the study sample (mean height of men = 1.73 m; mean height of women = 1.61 m), the overall mean BMI trend of 0.10 kg/m^2^ per year in men is equivalent to saying that men became, on average, 0.30 kg heavier each year. In women, the overall mean BMI trend of 0.15 kg/m^2^ per year is equivalent to saying that women became, on average, 0.39 kg heavier each year. Both of these mean body weight trends are higher (at least double) than the global mean body weight trend between 1975 and 2014 of 0.15 kg per year [4].

Over the past years in Brazil, there were increases in various indicators related to sedentary behavior, which may help explain the increases in overweight and obesity prevalence in the state capitals. For example, the proportion of people who did not practice any physical activity increased from 20.0% in 2008 to 62.1% in 2015 [20,21]; the mean daily time spent on browsing the internet increased from 3h39min in 2013 to 4h44min in 2016 [22,23]; the number of households with television increased from 63.3 million in 2013 to 66.1 million in 2015 [24,25]; and the number of cars increased from 27.9 million in 2006 to 51.3 million in 2016 [26]. Furthermore, there was also an increase in the mean annual amount of prepared foods purchased per capita, from 2.6 kg in 2002–2003 to 3.5 kg in 2008–2009 [27]. Broadly speaking, these reported trends reflect the worldwide increase in the number of people living in urban areas, where the forms of labor and transportation are mostly sedentary, the availability of outdoor recreational spaces is limited, and the availability of processed foods and the exposure to processed foods’ media are high [7,28].

Although obesity is increasing worldwide, it is particularly increasing more in less economically developed countries. From 1980 to 2008, the number of overweight or obese people in less economically developed countries increased from 250 million to 904 million (261.6% relative increase), while in more economically developed countries, the increase was from 321 million to 557 million (73.5% relative increase [6]). In this study, we found a similar pattern considering the context of Brazil’s state capitals. As seen in Figs 5 and 6, the largest increases in overweight and obesity prevalence were experienced, in general, in state capitals located in the north, northeast, and central-west regions (Fig 1), which are the less economically developed regions of Brazil [29]. Essentially, this result illustrates the relationship between body weight and socioeconomic status, which is consistently being verified in obesity studies. Monteiro et al. [30], for example, found that in Brazil between 1989 and 2003, obesity prevalence rate increased by 150% among low income men, while among high income men increased by 45%; among low income women, obesity prevalence rate increased by 26%, while among high income women decreased by 10%. In summary, obesity is particularly increasing more among low socioeconomic-level populations.

Among our results, the increases in severe and morbid obesity prevalence are of special concern, since higher BMI levels are associated with greater risk of morbidity and greater expenditure on healthcare. Ndumele et al. [31], analyzing data from the Atherosclerosis Risk in Communities (ARIC) Study, found that the risk of cardiovascular disease among overweight, obese, and severely obese individuals was, respectively, 26%, 53%, and 100% higher compared to normal weight individuals. Andreyeva et al. [32], analyzing data from the Health and Retirement Study (HRS), found that healthcare expenditure among moderately obese, severely obese, and morbidly obese individuals was, respectively, 25%, 50%, and 100% higher compared to normal weight individuals (these estimates are approximate). Furthermore, due to a greater risk of morbidity, other relevant burdens may be expected, including a decrease in workforce productivity, national output, and tax revenue; and an increase in government expenditure on incapacity and unemployment benefits [33].

The decrease in underweight prevalence found in most state capitals should be analyzed with caution. Although poverty, a major contributing factor of underweight [2], decreased significantly in Brazil over the past years [34], it should be noted that the participants of this study can support a residential fixed telephone line. In other words, the participants of this study may not be considered poor, or at least not sufficiently poor to be at risk of being underweight. Therefore, the decrease in Brazil’s levels of poverty, and also levels of undernourishment and food insecurity [34,35], may have had little influence in the decrease in underweight prevalence among our study participants. Furthermore, it is important to highlight that poverty, undernourishment, and food insecurity are usually more prevalent in rural areas [34]. It should also be mentioned that although the increase in overweight prevalence may help explain the decrease in underweight prevalence (since an increase in overweight prevalence would necessarily have led to a decrease in normal or underweight prevalence), it is important that other contributing factors be uncovered, as this information may allow improving underweight treatment and prevention strategies. Thus, more research is necessary to better understand the decrease in underweight prevalence.

The strengths of this study include: the large representative sample spanning 11 years; the fact that the sampling, recruitment, and data collection methods remained unchanged throughout the study period; the use of post-stratification weights; and the use of the age-standardization method. Post-stratification weights where used to correct for deviances in the probability of selection of the study participants, thus improving the representativeness of the study sample. The age-standardization method was used to control for the variability of the state capitals’ age distribution, thus improving the comparability of the body weight indicators.

The use of self-reported weight and height data may be interpreted as a limitation since it is a common behavior for survey participants to underreport their weights and over-report their heights, thus leading to the underestimation of BMI and, by extension, the underestimation of overweight and obesity prevalence [36,37]. Furthermore, studies have identified that weight underreporting tends to be more common in women, while height over-reporting tends to be more common in men [38–42]. A widely accepted hypothesis to explain the under/over-reporting of weight and height lies in the social desirability concept: respondents tailor their answers in order to conform to perceived societal norms [38]. Although self-report assessment may be susceptible to misreporting bias, it is important to highlight that it is an advantageous way of collecting data due to its low cost, ease of data collection, and the ability to efficiently collect data from a large number of individuals [43]. In addition, it should be mentioned that there are validation studies with Brazilian populations showing that self-reported weight and height data can be valid and reliable [44,45].

For the purpose of studying obesity, the use of BMI may be interpreted as a limitation since BMI is not a direct measure of body fat it may, therefore, be a source of classification bias. However, studies have shown high correlations between BMI and body fat [46,47]. Moreover, it should be highlighted that BMI is the standard measure of body weight in epidemiologic studies [11]. Thus, the presented results are broadly comparable, which may be considered an additional strength of this study.

Considering that the VIGITEL survey includes only individuals with fixed telephone lines, which are becoming less common in Brazil, it is valid to suggest the use of mobile telephone lines in future VIGITEL surveys, as this will allow assessing a more representative sample of the state capitals’ population. It is also valid to suggest the objective measuring of weight and height in a subsample of VIGITEL participants, as this will allow evaluating and correcting errors in self-reported BMI estimates. We also point out a promising research topic for future studies to address: the inference of the ages at which VIGITEL participants most likely transition to overweight and obesity status, which may contribute to enhancing prevention and control efforts [48].

To halt the increase in obesity in Brazil, the Federal Government has implemented policies such as the promotion of physical activity and healthy eating habits in public schools [49], the construction of public outdoor gyms [50], the free distribution of manuals on healthy eating [51], the launch of campaigns promoting breastfeeding [52], and the reduction of the minimum age, from 18 to 16 years, required for performing bariatric surgery in the public health system [53]. Furthermore, in 2011, the Federal Government made an agreement with the food industry to cut down 28 thousand tons of sodium from processed foods by 2020 [54]. Our results showed, however, that these and other policies were not able to halt the increase in obesity prevalence in the state capitals. Thus, a revision of Brazil’s policies for preventing and treating obesity is warranted. Brazilian authorities should perhaps consider implementing more mass-reaching policies such as Mexico’s sugar-sweetened beverage and nonessential energy-dense food tax [55], and Chile’s food labeling and advertising law [56]. In addition, although policies are necessary in all state capitals, our results suggest that policies are especially necessary in the north, northeast, and central-west regions’ state capitals, where, in general, the largest increases in overweight and obesity prevalence were experienced.

Lastly, we call attention to the reduction in obesity prevalence in Brazil’s state capitals necessary to meet the WHO’s global target of halting, by 2025, the increase in obesity prevalence at levels found in 2010 [57]. Considering that in 2010 the overall obesity prevalence was 14.6% in men and 15.2% in women, and that in 2016 the overall obesity prevalence was 18.1% in men and 18.8% in women, to reach the 2010 levels, the overall obesity prevalence would have to be reduced by 3.5 and 3.6 percentage points in men and women, respectively. Reducing obesity prevalence by these magnitudes represents a significant challenge, especially considering that in 33 years of population-level body weight monitoring, no country in the world has successfully reduced obesity [5]. Nevertheless, the stabilization in the mean BMI trend in Vitória among men (Fig 2A) supports hope that the obesity epidemic may be halted sometime in the future.

## Supporting information

S1 Table Age-standardized mean BMI (kg/m^2^) in Brazil’s state capitals, from 2006 to 2016, among men. Numbers in brackets show 95% confidence intervals.
(PDF)

S2 Table Age-standardized mean BMI (kg/m^2^) in Brazil’s state capitals, from 2006 to 2016, among women. Numbers in brackets show 95% confidence intervals.
(PDF)

S3 Table Age-standardized prevalence (%) of underweight (BMI < 18.5 kg/m^2^) in Brazil’s state capitals, from 2006 to 2016, among men. Numbers in brackets show 95% confidence intervals.
(PDF)

S4 Table Age-standardized prevalence (%) of underweight (BMI < 18.5 kg/m^2^) in Brazil’s state capitals, from 2006 to 2016, among women. Numbers in brackets show 95% confidence intervals.
(PDF)

S5 Table Age-standardized prevalence (%) of normal weight (18.5 kg/m^2^ ≤ BMI < 25 kg/m^2^) in Brazil’s state capitals, from 2006 to 2016, among men. Numbers in brackets show 95% confidence intervals.
(PDF)

S6 Table Age-standardized prevalence (%) of normal weight (18.5 kg/m^2^ ≤ BMI < 25 kg/m^2^) in Brazil’s state capitals, from 2006 to 2016, among women. Numbers in brackets show 95% confidence intervals.
(PDF)

S7 Table Age-standardized prevalence (%) of pre-obesity (25 kg/m^2^ ≤ BMI < 30 kg/m^2^) in Brazil’s state capitals, from 2006 to 2016, among men. Numbers in brackets show 95% confidence intervals.
(PDF)

S8 Table Age-standardized prevalence (%) of pre-obesity (25 kg/m^2^ ≤ BMI < 30 kg/m^2^) in Brazil’s state capitals, from 2006 to 2016, among women. Numbers in brackets show 95% confidence intervals.
(PDF)

S9 Table Age-standardized prevalence (%) of moderate obesity (30 kg/m^2^ ≤ BMI < 35 kg/m^2^) in Brazil’s state capitals, from 2006 to 2016, among men. Numbers in brackets show 95% confidence intervals.
(PDF)

S10 Table Age-standardized prevalence (%) of moderate obesity (30 kg/m^2^ ≤ BMI < 35 kg/m^2^) in Brazil’s state capitals, from 2006 to 2016, among women. Numbers in brackets show 95% confidence intervals.
(PDF)

S11 Table Age-standardized prevalence (%) of severe obesity (35 kg/m^2^ ≤ BMI < 40 kg/m^2^) in Brazil’s state capitals, from 2006 to 2016, among men. Numbers in brackets show 95% confidence intervals.
(PDF)

S12 Table Age-standardized prevalence (%) of severe obesity (35 kg/m^2^ ≤ BMI < 40 kg/m^2^) in Brazil’s state capitals, from 2006 to 2016, among women. Numbers in brackets show 95% confidence intervals.
(PDF)

S13 Table Age-standardized prevalence (%) of morbid obesity (BMI ≥ 40 kg/m^2^) in Brazil’s state capitals, from 2006 to 2016, among men. Numbers in brackets show 95% confidence intervals.
(PDF)

S14 Table Age-standardized prevalence (%) of morbid obesity (BMI ≥ 40 kg/m^2^) in Brazil’s state capitals, from 2006 to 2016, among women. Numbers in brackets show 95% confidence intervals.
(PDF)

S15 Table Age-standardized prevalence (%) of overweight (BMI ≥ 25 kg/m^2^) in Brazil’s state capitals, from 2006 to 2016, among men. Numbers in brackets show 95% confidence intervals.
(PDF)

S16 Table Age-standardized prevalence (%) of overweight (BMI ≥ 25 kg/m^2^) in Brazil’s state capitals, from 2006 to 2016, among women. Numbers in brackets show 95% confidence intervals.
(PDF)

S17 Table Age-standardized prevalence (%) of obesity (BMI ≥ 30 kg/m^2^) in Brazil’s state capitals, from 2006 to 2016, among men. Numbers in brackets show 95% confidence intervals.
(PDF)

S18 Table Age-standardized prevalence (%) of obesity (BMI ≥ 30 kg/m^2^) in Brazil’s state capitals, from 2006 to 2016, among women. Numbers in brackets show 95% confidence intervals.
(PDF)
