# Supplementary material for: Adult body weight trends in 27 urban populations of Brazil from 2006 to 2016: A population-based study
Source: PLoS One. 2019 Mar 6;14(3):e0213254. doi: 10.1371/journal.pone.0213254 (PMC6402686; doi:10.1371/journal.pone.0213254)
Supplement: S1 Table — Numbers in brackets show 95% confidence intervals. (PDF) [file pone.0213254.s001.pdf]

S1 Table. Age-standardized mean BMI (kg/m²) in Brazil's state capitals, from 2006 to 2016, among men. Numbers in brackets show 95% confidence intervals.

State capital	2006	2007	2008	2009	2010	2011	2012	2013	2014	2015	2016
Aracaju	25.7 (25.2-26.2)	25.1 (24.7-25.5)	25.3 (24.9-25.7)	26.0 (25.4-26.5)	25.9 (25.4-26.5)	25.6 (25.1-26.0)	26.7 (26.1-27.3)	25.7 (25.2-26.2)	26.1 (25.6-26.7)	26.1 (25.7-26.5)	26.5 (26.0-27.0)
Belém	25.9 (25.4-26.3)	25.6 (25.2-26.0)	25.8 (25.4-26.1)	25.7 (25.3-26.0)	25.9 (25.5-26.4)	25.7 (25.3-26.2)	26.1 (25.7-26.5)	26.0 (25.6-26.3)	26.6 (26.1-27.1)	26.4 (25.9-26.9)	26.7 (26.2-27.1)
Belo Horizonte	25.1 (24.8-25.4)	25.2 (24.9-25.6)	25.3 (24.9-25.6)	25.4 (25.0-25.7)	25.5 (25.1-25.8)	25.4 (25.1-25.7)	25.6 (25.2-26.0)	25.3 (24.9-25.7)	25.6 (25.1-26.1)	25.9 (25.6-26.3)	25.7 (25.3-26.0)
Boa Vista	25.6 (25.2-26.0)	25.5 (25.1-25.8)	25.8 (25.3-26.2)	26.0 (25.5-26.4)	25.7 (25.3-26.1)	26.1 (25.7-26.5)	25.8 (25.4-26.2)	26.4 (26.0-26.8)	26.8 (26.2-27.4)	27.2 (26.6-27.7)	26.8 (26.2-27.3)
Campo Grande	25.6 (25.1-26.0)	25.8 (25.4-26.2)	26.0 (25.6-26.4)	25.9 (25.5-26.2)	26.1 (25.7-26.5)	26.3 (26.0-26.7)	26.4 (26.0-26.9)	26.1 (25.7-26.5)	26.4 (25.9-26.9)	26.6 (26.2-27.0)	26.9 (26.4-27.4)
Cuiabá	25.7 (25.3-26.0)	26.0 (25.6-26.4)	25.9 (25.5-26.3)	25.7 (25.3-26.0)	26.0 (25.6-26.4)	26.3 (25.9-26.6)	26.5 (26.1-27.0)	26.7 (26.2-27.2)	26.7 (26.1-27.3)	26.3 (25.9-26.8)	27.0 (26.6-27.4)
Curitiba	25.7 (25.4-26.1)	25.7 (25.4-26.0)	25.7 (25.4-26.0)	25.8 (25.5-26.1)	26.1 (25.7-26.5)	26.1 (25.8-26.4)	26.1 (25.7-26.5)	26.4 (26.0-26.9)	26.1 (25.6-26.6)	26.2 (25.9-26.5)	26.8 (26.3-27.4)
Federal District	25.3 (24.9-25.7)	25.1 (24.8-25.4)	25.2 (24.9-25.6)	25.1 (24.6-25.5)	25.5 (25.0-26.0)	25.8 (25.5-26.1)	25.6 (25.2-26.0)	26.0 (25.7-26.4)	26.1 (25.6-26.6)	25.8 (25.2-26.3)	25.8 (25.2-26.3)
Florianópolis	25.6 (25.3-25.8)	25.7 (25.4-26.0)	25.7 (25.3-26.1)	25.8 (25.5-26.1)	25.9 (25.6-26.3)	26.3 (25.9-26.7)	26.0 (25.6-26.5)	26.1 (25.7-26.5)	26.3 (25.7-27.0)	26.4 (26.0-26.8)	26.1 (25.6-26.7)
Fortaleza	25.4 (25.0-25.9)	25.6 (25.2-26.0)	25.9 (25.5-26.3)	25.7 (25.3-26.1)	26.4 (25.9-26.8)	26.5 (26.1-26.9)	26.1 (25.7-26.6)	26.2 (25.7-26.6)	26.7 (26.1-27.4)	26.9 (26.5-27.3)	26.7 (26.2-27.2)
Goiânia	24.9 (24.6-25.3)	25.2 (24.8-25.6)	25.2 (24.9-25.5)	25.5 (25.1-25.8)	25.5 (25.1-25.8)	25.8 (25.5-26.1)	25.7 (25.3-26.1)	25.8 (25.4-26.2)	25.5 (25.2-25.9)	25.8 (25.2-26.4)	26.1 (25.6-26.6)
João Pessoa	25.7 (25.2-26.1)	25.8 (25.4-26.2)	25.9 (25.4-26.4)	25.2 (24.8-25.5)	25.8 (25.3-26.3)	26.1 (25.7-26.6)	26.3 (25.7-26.8)	26.0 (25.6-26.4)	26.2 (25.8-26.7)	26.0 (25.6-26.5)	26.7 (26.1-27.3)
Macapá	25.9 (25.5-26.3)	26.1 (25.7-26.6)	26.0 (25.6-26.4)	25.7 (25.2-26.2)	26.2 (25.8-26.6)	26.4 (26.0-26.8)	25.9 (25.4-26.4)	26.8 (26.3-27.4)	26.0 (25.5-26.6)	26.8 (26.2-27.5)	26.5 (25.7-27.3)

Maceió	25.0 (24.7-25.4)	25.3 (24.9-25.7)	25.5 (25.0-26.0)	25.3 (24.6-25.9)	25.8 (25.2-26.4)	26.3 (25.8-26.8)	26.1 (25.7-26.6)	26.3 (25.8-26.8)	26.0 (25.5-26.5)	25.9 (25.5-26.3)	26.4 (25.9-26.9)
Manaus	26.0 (25.6-26.3)	25.8 (25.5-26.1)	25.4 (25.0-25.8)	25.9 (25.4-26.3)	26.1 (25.7-26.5)	26.5 (26.1-26.9)	25.8 (25.3-26.4)	26.4 (25.9-26.8)	26.8 (26.2-27.4)	27.8 (27.1-28.5)	26.7 (26.3-27.2)
Natal	26.0 (25.6-26.5)	26.0 (25.6-26.4)	25.5 (25.1-25.9)	25.9 (25.5-26.3)	25.7 (25.2-26.1)	26.5 (25.9-27.0)	26.4 (25.8-27.1)	26.6 (26.1-27.2)	25.9 (25.3-26.4)	26.4 (25.8-26.9)	26.5 (26.0-27.0)
Palmas	25.2 (24.8-25.7)	24.9 (24.5-25.4)	25.6 (25.2-26.0)	25.4 (25.0-25.8)	26.0 (25.3-26.7)	25.5 (25.2-25.9)	26.0 (25.5-26.5)	26.7 (26.0-27.4)	26.3 (25.8-26.9)	26.0 (25.7-26.4)	26.0 (25.6-26.4)
Porto Alegre	25.9 (25.6-26.3)	25.6 (25.3-25.9)	26.0 (25.6-26.3)	25.6 (25.3-25.9)	26.1 (25.7-26.5)	26.4 (26.1-26.8)	26.4 (26.0-26.8)	26.8 (26.3-27.3)	26.8 (26.2-27.5)	27.2 (26.7-27.7)	26.6 (26.2-27.0)
Porto Velho	25.4 (25.1-25.8)	25.7 (25.3-26.1)	25.9 (25.3-26.4)	26.4 (26.0-26.9)	25.9 (25.5-26.2)	26.1 (25.7-26.4)	26.1 (25.6-26.6)	26.6 (26.1-27.0)	26.9 (26.3-27.5)	26.3 (25.8-26.9)	26.7 (26.1-27.3)
Recife	25.3 (25.0-25.7)	25.4 (25.0-25.8)	25.4 (25.0-25.9)	25.9 (25.4-26.4)	26.2 (25.7-26.6)	25.9 (25.5-26.3)	25.9 (25.5-26.3)	25.9 (25.4-26.3)	25.5 (25.0-26.0)	25.8 (25.4-26.2)	26.5 (26.1-26.9)
Rio Branco	25.3 (24.9-25.7)	25.7 (25.1-26.4)	26.0 (25.5-26.5)	25.8 (25.2-26.3)	26.5 (25.8-27.2)	26.4 (25.9-26.8)	26.3 (25.8-26.8)	26.4 (26.0-26.9)	27.0 (26.1-28.0)	26.7 (26.2-27.2)	27.5 (27.0-27.9)
Rio de Janeiro	25.6 (25.3-26.0)	25.8 (25.4-26.2)	25.4 (25.0-25.7)	25.9 (25.5-26.3)	25.8 (25.5-26.2)	26.3 (25.9-26.7)	26.3 (25.8-26.8)	26.3 (25.9-26.8)	26.4 (25.9-26.9)	26.2 (25.7-26.6)	26.8 (26.2-27.3)
Salvador	24.9 (24.5-25.2)	25.2 (24.4-26.0)	25.2 (24.8-25.5)	25.1 (24.8-25.5)	25.0 (24.6-25.4)	25.4 (25.0-25.7)	25.3 (24.9-25.6)	25.4 (25.0-25.8)	26.0 (25.5-26.4)	25.7 (25.3-26.1)	25.9 (25.4-26.4)
São Luís	24.8 (24.4-25.1)	24.9 (24.5-25.3)	25.1 (24.7-25.5)	25.3 (24.8-25.7)	25.4 (25.0-25.8)	25.3 (24.9-25.7)	25.8 (25.4-26.3)	25.4 (25.0-25.8)	25.4 (25.0-25.9)	26.0 (25.6-26.5)	25.7 (25.3-26.1)
São Paulo	25.3 (24.9-25.7)	25.8 (25.4-26.1)	25.9 (25.6-26.2)	25.6 (25.3-26.0)	25.8 (25.4-26.1)	25.8 (25.4-26.1)	26.1 (25.7-26.4)	26.2 (25.8-26.6)	26.1 (25.7-26.6)	26.4 (26.0-26.9)	26.2 (25.8-26.6)
Teresina	24.9 (24.5-25.2)	24.9 (24.5-25.3)	25.0 (24.6-25.4)	25.0 (24.6-25.4)	25.5 (25.1-25.9)	25.6 (25.2-25.9)	25.8 (25.3-26.3)	26.2 (25.7-26.6)	26.0 (25.5-26.4)	25.9 (25.5-26.3)	26.3 (25.8-26.8)
Vitória	25.3 (24.9-25.7)	25.7 (25.3-26.0)	25.4 (25.1-25.7)	25.5 (25.2-25.8)	25.9 (25.6-26.3)	25.7 (25.3-26.1)	26.1 (25.7-26.5)	26.1 (25.7-26.5)	26.0 (25.5-26.5)	25.6 (25.2-26.0)	25.7 (25.3-26.1)
State capitals overall	25.4 (25.3-25.5)	25.6 (25.5-25.7)	25.6 (25.5-25.7)	25.6 (25.5-25.7)	25.8 (25.7-25.9)	25.9 (25.8-26.1)	26.0 (25.9-26.1)	26.1 (26.0-26.3)	26.2 (26.1-26.4)	26.3 (26.2-26.5)	26.3 (26.2-26.5)
