# Supplementary material for: Adult body weight trends in 27 urban populations of Brazil from 2006 to 2016: A population-based study
Source: PLoS One. 2019 Mar 6;14(3):e0213254. doi: 10.1371/journal.pone.0213254 (PMC6402686; doi:10.1371/journal.pone.0213254)
Supplement: S2 Table — Numbers in brackets show 95% confidence intervals. (PDF) [file pone.0213254.s002.pdf]

S2 Table. Age-standardized mean BMI (kg/m²) in Brazil's state capitals, from 2006 to 2016, among women. Numbers in brackets show 95% confidence intervals.

State capital	2006	2007	2008	2009	2010	2011	2012	2013	2014	2015	2016
Aracaju	24.2 (23.9-24.6)	24.6 (24.2-25.0)	24.8 (24.4-25.2)	25.0 (24.6-25.4)	25.3 (24.9-25.6)	25.3 (24.9-25.6)	25.4 (25.0-25.8)	25.5 (25.1-25.9)	25.4 (25.1-25.8)	25.6 (25.2-26.0)	26.1 (25.6-26.5)
Belém	24.2 (23.9-24.6)	24.3 (24.0-24.7)	24.8 (24.4-25.2)	24.8 (24.4-25.2)	24.7 (24.4-25.0)	25.0 (24.6-25.3)	25.4 (24.9-25.9)	25.4 (25.0-25.7)	25.7 (25.2-26.2)	25.7 (25.3-26.1)	25.6 (25.2-26.0)
Belo Horizonte	23.9 (23.6-24.2)	24.1 (23.8-24.4)	24.4 (24.1-24.8)	24.5 (24.2-24.8)	24.5 (24.2-24.9)	25.0 (24.6-25.3)	25.1 (24.8-25.4)	25.1 (24.8-25.5)	25.4 (24.9-25.8)	25.7 (25.3-26.1)	25.2 (24.9-25.6)
Boa Vista	24.4 (24.0-24.8)	24.6 (24.2-24.9)	24.9 (24.5-25.3)	25.1 (24.7-25.5)	25.6 (25.2-26.0)	25.3 (24.9-25.6)	25.5 (25.2-25.9)	25.6 (25.2-26.0)	25.4 (24.9-25.8)	26.2 (25.8-26.7)	25.4 (25.0-25.8)
Campo Grande	24.6 (24.3-25.0)	24.9 (24.5-25.2)	24.9 (24.6-25.3)	25.5 (25.2-25.8)	25.6 (25.3-26.0)	25.5 (25.1-25.8)	26.1 (25.7-26.6)	25.8 (25.3-26.2)	26.1 (25.6-26.7)	26.5 (26.2-26.9)	26.0 (25.5-26.4)
Cuiabá	24.5 (24.1-24.9)	25.0 (24.7-25.4)	25.0 (24.6-25.3)	25.0 (24.6-25.3)	25.5 (25.1-25.9)	25.5 (25.1-25.9)	25.6 (25.0-26.1)	26.0 (25.6-26.4)	26.3 (25.8-26.8)	25.6 (25.1-26.1)	25.8 (25.5-26.2)
Curitiba	24.5 (24.3-24.8)	24.6 (24.3-25.0)	24.9 (24.6-25.3)	24.9 (24.6-25.1)	25.2 (24.9-25.5)	25.1 (24.8-25.5)	25.4 (25.0-25.8)	25.4 (25.0-25.8)	26.0 (25.5-26.5)	25.7 (25.3-26.0)	25.4 (25.0-25.9)
Federal District	24.1 (23.8-24.5)	24.5 (24.2-24.8)	24.7 (24.4-25.0)	24.5 (24.0-25.0)	24.4 (23.9-25.0)	25.2 (24.9-25.5)	25.2 (24.9-25.6)	25.2 (24.9-25.6)	25.6 (25.1-26.0)	24.9 (24.4-25.3)	25.3 (24.8-25.7)
Florianópolis	24.0 (23.7-24.3)	24.1 (23.7-24.5)	24.2 (23.9-24.5)	24.7 (24.3-25.0)	24.8 (24.5-25.1)	24.6 (24.3-24.9)	25.2 (24.7-25.6)	24.8 (24.5-25.1)	24.9 (24.5-25.3)	25.0 (24.6-25.4)	24.8 (24.3-25.2)
Fortaleza	24.2 (23.9-24.6)	24.6 (24.2-25.0)	24.7 (24.4-25.1)	25.3 (24.9-25.7)	25.6 (25.2-26.0)	25.5 (25.1-25.9)	25.6 (25.2-26.0)	25.6 (25.2-26.0)	25.9 (25.3-26.5)	25.8 (25.5-26.1)	25.9 (25.5-26.4)
Goiânia	23.9 (23.6-24.2)	24.2 (23.9-24.4)	24.2 (23.9-24.5)	24.6 (24.3-24.9)	24.4 (24.1-24.7)	24.8 (24.5-25.1)	25.2 (24.9-25.6)	25.0 (24.7-25.3)	25.4 (24.8-25.9)	24.7 (24.2-25.2)	25.1 (24.7-25.4)
João Pessoa	24.4 (24.1-24.8)	24.5 (24.2-24.9)	25.1 (24.6-25.5)	25.2 (24.8-25.6)	24.9 (24.5-25.3)	25.2 (24.7-25.6)	25.6 (25.1-26.0)	25.4 (24.9-25.8)	25.3 (24.9-25.7)	25.9 (25.5-26.3)	26.1 (25.7-26.6)
Macapá	24.5 (24.1-25.0)	25.0 (24.6-25.4)	25.3 (24.9-25.7)	25.4 (24.9-25.9)	25.7 (25.0-26.3)	25.7 (25.2-26.1)	26.1 (25.6-26.6)	25.2 (24.8-25.6)	26.2 (25.6-26.7)	26.1 (25.6-26.6)	25.7 (25.2-26.1)

Maceió	24.3 (23.9-24.6)	24.6 (24.2-25.0)	25.2 (24.7-25.7)	25.1 (24.6-25.5)	24.9 (24.5-25.3)	25.3 (24.8-25.7)	25.5 (25.0-26.0)	25.5 (25.1-26.0)	26.0 (25.5-26.5)	26.1 (25.6-26.5)	26.3 (25.8-26.8)
Manaus	25.0 (24.6-25.3)	24.9 (24.5-25.3)	25.2 (24.8-25.6)	25.4 (25.0-25.8)	25.7 (25.3-26.1)	26.1 (25.6-26.5)	26.1 (25.6-26.6)	26.0 (25.6-26.4)	26.2 (25.7-26.8)	26.9 (26.3-27.5)	26.0 (25.6-26.4)
Natal	24.5 (24.1-24.8)	24.9 (24.5-25.3)	24.9 (24.5-25.2)	25.0 (24.6-25.4)	25.6 (25.3-26.0)	25.6 (25.2-26.0)	26.1 (25.6-26.5)	25.3 (24.9-25.7)	26.0 (25.5-26.4)	26.0 (25.5-26.4)	26.1 (25.7-26.6)
Palmas	24.0 (23.6-24.4)	24.1 (23.7-24.5)	24.3 (23.8-24.7)	23.9 (23.6-24.3)	24.6 (24.2-25.0)	24.8 (24.4-25.2)	25.3 (24.7-25.9)	25.1 (24.7-25.5)	25.4 (25.0-25.9)	24.9 (24.6-25.3)	25.2 (24.7-25.6)
Porto Alegre	24.8 (24.4-25.1)	24.6 (24.3-25.0)	25.0 (24.6-25.3)	25.3 (24.9-25.7)	25.5 (25.1-25.8)	25.8 (25.4-26.1)	25.6 (25.2-26.0)	25.3 (24.9-25.7)	25.9 (25.4-26.4)	25.7 (25.2-26.2)	25.8 (25.2-26.5)
Porto Velho	24.6 (24.2-24.9)	25.0 (24.5-25.4)	25.1 (24.8-25.4)	25.3 (24.9-25.8)	25.8 (25.3-26.2)	25.5 (25.0-26.0)	25.8 (25.3-26.2)	25.6 (25.2-26.1)	26.1 (25.3-26.9)	26.5 (26.0-27.0)	26.0 (25.6-26.5)
Recife	24.7 (24.4-25.1)	24.8 (24.4-25.2)	24.7 (24.3-25.0)	24.7 (24.4-25.1)	25.3 (24.9-25.7)	25.2 (24.9-25.5)	25.7 (25.1-26.2)	25.6 (25.2-26.0)	26.1 (25.5-26.6)	26.0 (25.6-26.4)	26.1 (25.6-26.5)
Rio Branco	24.9 (24.5-25.3)	25.0 (24.7-25.4)	25.7 (25.1-26.3)	25.5 (25.0-25.9)	25.7 (25.2-26.2)	25.9 (25.5-26.3)	26.2 (25.7-26.7)	26.1 (25.7-26.6)	25.9 (25.4-26.4)	26.6 (26.1-27.2)	26.6 (26.2-27.0)
Rio de Janeiro	24.6 (24.3-24.9)	24.7 (24.3-25.0)	24.9 (24.5-25.2)	25.1 (24.7-25.4)	25.4 (25.0-25.7)	25.2 (24.9-25.6)	25.8 (25.3-26.3)	25.5 (25.1-25.9)	25.9 (25.4-26.5)	26.3 (25.6-26.9)	26.1 (25.5-26.6)
Salvador	24.5 (24.2-24.8)	24.6 (24.3-25.0)	24.9 (24.4-25.3)	25.1 (24.8-25.5)	25.1 (24.8-25.5)	25.3 (24.9-25.6)	25.6 (25.1-26.0)	25.4 (25.0-25.7)	25.6 (25.1-26.1)	25.5 (25.2-25.9)	26.1 (25.6-26.5)
São Luís	23.7 (23.3-24.1)	24.1 (23.6-24.5)	23.8 (23.5-24.2)	24.4 (24.1-24.8)	24.6 (24.2-24.9)	24.9 (24.5-25.3)	24.8 (24.4-25.2)	24.7 (24.3-25.1)	25.4 (24.9-25.9)	24.9 (24.6-25.3)	25.3 (24.9-25.8)
São Paulo	24.7 (24.4-25.0)	24.5 (24.2-24.8)	24.6 (24.3-24.9)	25.0 (24.6-25.3)	25.1 (24.8-25.5)	25.3 (25.0-25.6)	25.5 (25.1-25.9)	25.4 (25.1-25.8)	25.3 (25.0-25.7)	26.4 (25.6-27.3)	25.8 (25.5-26.2)
Teresina	23.9 (23.5-24.2)	24.3 (23.9-24.7)	24.4 (24.0-24.7)	24.4 (24.1-24.7)	24.4 (24.0-24.8)	24.7 (24.3-25.1)	24.8 (24.4-25.3)	25.2 (24.8-25.6)	25.2 (24.6-25.8)	25.4 (25.0-25.8)	25.2 (24.8-25.7)
Vitória	24.0 (23.7-24.3)	24.0 (23.7-24.3)	24.5 (24.2-24.9)	24.8 (24.4-25.1)	24.9 (24.5-25.2)	25.1 (24.7-25.4)	24.9 (24.5-25.2)	25.3 (24.9-25.7)	25.4 (25.0-25.8)	25.5 (25.0-25.9)	25.2 (24.8-25.6)
State capitals overall	24.5 (24.4-24.6)	24.5 (24.4-24.6)	24.7 (24.6-24.8)	24.9 (24.8-25.1)	25.1 (25.0-25.2)	25.3 (25.2-25.4)	25.5 (25.4-25.7)	25.4 (25.3-25.6)	25.6 (25.5-25.8)	25.9 (25.7-26.1)	25.8 (25.6-25.9)
