# Supplementary material for: Adult body weight trends in 27 urban populations of Brazil from 2006 to 2016: A population-based study
Source: PLoS One. 2019 Mar 6;14(3):e0213254. doi: 10.1371/journal.pone.0213254 (PMC6402686; doi:10.1371/journal.pone.0213254)
Supplement: S3 Table — Numbers in brackets show 95% confidence intervals. (PDF) [file pone.0213254.s003.pdf]

**S3 Table. Age-standardized prevalence (%) of underweight (BMI < 18.5 kg/m²) in Brazil's state capitals, from 2006 to 2016, among men.** Numbers in brackets show 95% confidence intervals.

| State capital | 2006 | 2007 | 2008 | 2009 | 2010 | 2011 | 2012 | 2013 | 2014 | 2015 | 2016 |
|---|---|---|---|---|---|---|---|---|---|---|---|
| Aracaju | 2.9 (1.3-4.5) | 2.1 (0.9-3.3) | 4.4 (2.1-6.7) | 3.7 (1.5-6.0) | 4.0 (2.1-6.0) | 3.3 (1.4-5.1) | 1.8 (0.4-3.3) | 3.3 (1.1-5.4) | 3.9 (1.4-6.3) | 1.5 (0.3-2.6) | 3.0 (1.4-4.5) |
| Belém | 2.6 (1.1-4.1) | 4.3 (2.5-6.1) | 1.8 (0.7-3.0) | 2.5 (0.6-4.4) | 4.8 (2.0-7.6) | 3.6 (1.7-5.5) | 2.2 (0.9-3.5) | 2.9 (1.2-4.6) | 0.8 (-0.1-1.7) | 5.2 (2.6-7.8) | 1.0 (0.2-1.9) |
| Belo Horizonte | 1.6 (0.6-2.6) | 2.8 (1.2-4.3) | 2.6 (1.0-4.1) | 3.4 (1.7-5.2) | 1.5 (0.5-2.6) | 3.1 (1.5-4.6) | 1.6 (-0.1-3.4) | 3.6 (1.8-5.4) | 3.1 (1.2-4.9) | 1.6 (0.5-2.7) | 2.7 (1.2-4.2) |
| Boa Vista | 2.6 (0.9-4.3) | 2.7 (1.2-4.3) | 1.0 (0.3-1.7) | 2.1 (0.5-3.7) | 2.9 (0.8-5.0) | 2.1 (0.6-3.7) | 3.7 (1.9-5.5) | 1.9 (0.6-3.1) | 2.3 (0.7-3.9) | 1.4 (0.4-2.3) | 1.0 (0.4-1.7) |
| Campo Grande | 4.1 (2.1-6.0) | 1.7 (0.8-2.6) | 0.7 (0.1-1.4) | 1.8 (0.8-2.7) | 1.6 (0.6-2.5) | 1.1 (0.3-1.8) | 3.6 (1.4-5.7) | 1.6 (0.5-2.7) | 1.1 (0.0-2.3) | 1.8 (0.7-2.9) | 1.7 (0.7-2.7) |
| Cuiabá | 2.2 (0.7-3.6) | 2.3 (0.8-3.8) | 2.3 (1.0-3.7) | 2.5 (1.2-3.7) | 3.7 (2.0-5.3) | 4.1 (2.3-5.9) | 2.5 (0.7-4.4) | 1.9 (0.5-3.3) | 2.5 (0.8-4.1) | 1.5 (0.1-3.0) | 2.1 (1.0-3.3) |
| Curitiba | 1.1 (0.1-2.2) | 0.5 (-0.1-1.0) | 1.8 (0.6-2.9) | 1.0 (0.3-1.7) | 1.4 (0.4-2.4) | 1.4 (0.3-2.5) | 2.3 (0.7-3.9) | 1.2 (0.4-2.0) | 3.7 (1.2-6.2) | 1.1 (0.2-1.9) | 1.8 (0.4-3.1) |
| Federal District | 1.4 (0.2-2.5) | 1.0 (0.1-1.9) | 2.1 (0.8-3.4) | 0.9 (0.0-1.8) | 6.2 (1.7-10.7) | 1.8 (0.4-3.1) | 3.3 (1.6-5.0) | 2.1 (0.8-3.3) | 2.9 (0.9-4.8) | 4.9 (0.9-8.9) | 3.1 (1.2-5.0) |
| Florianópolis | 1.3 (0.4-2.3) | 1.0 (0.2-1.7) | 1.3 (0.3-2.3) | 1.2 (0.3-2.1) | 1.7 (0.5-2.9) | 1.3 (0.2-2.4) | 1.7 (0.3-3.1) | 1.8 (0.5-3.1) | 2.1 (0.4-3.9) | 2.8 (1.2-4.5) | 2.9 (0.2-5.6) |
| Fortaleza | 3.3 (1.4-5.3) | 2.8 (1.1-4.4) | 2.7 (1.0-4.5) | 2.8 (0.6-5.0) | 3.2 (1.2-5.1) | 1.3 (0.3-2.4) | 2.2 (0.9-3.6) | 3.2 (1.2-5.3) | 1.8 (0.3-3.3) | 1.5 (0.6-2.5) | 1.6 (0.4-2.8) |
| Goiânia | 3.5 (2.0-5.0) | 2.3 (1.2-3.5) | 2.4 (1.3-3.6) | 1.7 (0.7-2.7) | 3.3 (1.7-4.9) | 1.7 (0.6-2.8) | 2.9 (1.5-4.3) | 2.7 (1.0-4.4) | 4.1 (2.2-6.0) | 3.0 (0.1-5.9) | 2.4 (0.8-4.0) |
| João Pessoa | 3.7 (2.0-5.4) | 2.0 (0.6-3.5) | 3.0 (1.1-5.0) | 3.5 (1.7-5.3) | 1.7 (0.6-2.7) | 2.9 (1.3-4.5) | 3.6 (0.3-7.0) | 3.2 (1.4-5.0) | 2.7 (0.7-4.6) | 3.0 (1.3-4.8) | 1.6 (0.4-2.8) |
| Macapá | 2.9 (1.4-4.3) | 2.5 (0.8-4.3) | 2.2 (1.2-3.2) | 3.6 (1.1-6.2) | 1.7 (0.4-3.0) | 2.7 (1.5-4.0) | 2.6 (0.8-4.4) | 1.2 (0.0-2.4) | 3.6 (1.2-5.9) | 2.6 (0.7-4.5) | 3.3 (1.5-5.2) |

| | | | | | | | | | | | |
|---|---|---|---|---|---|---|---|---|---|---|---|
| Maceió | 3.5 (1.7-5.3) | 3.3 (1.5-5.2) | 2.2 (1.0-3.3) | 4.0 (1.3-6.7) | 3.5 (1.4-5.6) | 2.1 (0.6-3.6) | 1.5 (0.2-2.8) | 3.5 (1.0-6.1) | 1.5 (0.4-2.6) | 2.8 (1.2-4.3) | 3.4 (0.7-6.0) |
| Manaus | 3.0 (1.2-4.7) | 2.0 (0.8-3.1) | 3.7 (1.9-5.6) | 2.6 (0.8-4.4) | 2.8 (1.0-4.7) | 2.1 (0.9-3.3) | 3.0 (1.4-4.7) | 4.1 (1.8-6.4) | 1.5 (0.4-2.6) | 1.6 (0.3-2.8) | 1.6 (0.5-2.7) |
| Natal | 0.9 (0.2-1.7) | 1.7 (0.6-2.8) | 2.2 (0.8-3.7) | 1.4 (0.4-2.3) | 2.4 (0.9-3.9) | 1.5 (0.5-2.4) | 3.7 (1.9-5.6) | 2.1 (0.5-3.8) | 4.4 (1.2-7.6) | 3.1 (1.5-4.8) | 1.5 (0.3-2.7) |
| Palmas | 2.6 (1.2-3.9) | 4.5 (2.1-6.9) | 1.9 (0.5-3.3) | 2.7 (1.3-4.1) | 3.7 (1.1-6.3) | 3.5 (1.5-5.5) | 2.1 (0.9-3.4) | 1.8 (0.4-3.2) | 1.9 (0.5-3.3) | 1.7 (0.5-3.0) | 1.8 (0.7-3.0) |
| Porto Alegre | 1.3 (-0.2-2.7) | 0.9 (0.1-1.7) | 1.1 (0.3-2.0) | 0.1 (-0.0-0.3) | 2.2 (0.0-4.4) | 0.9 (0.1-1.7) | 1.5 (0.4-2.5) | 1.2 (0.5-1.9) | 3.6 (0.3-6.9) | 1.1 (0.3-1.9) | 2.2 (0.6-3.7) |
| Porto Velho | 2.6 (1.1-4.1) | 2.6 (1.1-4.0) | 1.8 (0.6-2.9) | 3.0 (1.3-4.7) | 1.7 (0.6-2.9) | 3.7 (1.8-5.6) | 2.6 (0.6-4.6) | 2.7 (0.6-4.8) | 1.2 (0.1-2.3) | 2.1 (0.8-3.4) | 1.5 (0.5-2.5) |
| Recife | 3.9 (1.6-6.2) | 3.4 (1.8-5.1) | 3.4 (1.7-5.1) | 3.8 (1.9-5.8) | 3.0 (1.3-4.7) | 2.9 (1.2-4.6) | 2.9 (0.9-4.9) | 3.9 (2.1-5.8) | 4.6 (1.6-7.6) | 2.2 (0.7-3.7) | 1.5 (0.6-2.5) |
| Rio Branco | 3.2 (1.7-4.7) | 2.2 (0.8-3.5) | 2.3 (1.0-3.7) | 4.1 (0.8-7.4) | 1.8 (0.5-3.1) | 1.7 (0.8-2.5) | 2.8 (1.0-4.5) | 1.3 (0.3-2.3) | 1.4 (-0.0-2.9) | 1.3 (0.4-2.3) | 0.9 (0.2-1.7) |
| Rio de Janeiro | 2.0 (0.8-3.2) | 1.7 (0.6-2.8) | 3.3 (1.7-5.0) | 1.6 (0.4-2.8) | 2.5 (0.9-4.1) | 2.7 (1.3-4.1) | 1.5 (0.3-2.7) | 2.7 (1.2-4.2) | 1.7 (0.4-3.0) | 2.3 (0.2-4.5) | 1.8 (0.5-3.0) |
| Salvador | 3.0 (1.6-4.4) | 5.7 (3.4-8.0) | 2.3 (1.0-3.7) | 4.6 (2.8-6.5) | 2.5 (1.2-3.9) | 1.3 (0.4-2.1) | 1.9 (0.8-3.1) | 3.7 (2.0-5.4) | 2.1 (0.6-3.6) | 4.2 (1.7-6.6) | 2.7 (1.3-4.2) |
| São Luís | 4.6 (2.9-6.4) | 4.6 (2.3-6.9) | 3.7 (1.9-5.4) | 4.5 (2.8-6.2) | 2.8 (1.2-4.3) | 4.7 (2.3-7.0) | 3.3 (1.6-5.0) | 4.1 (2.2-6.1) | 3.0 (1.0-4.9) | 2.7 (1.4-4.1) | 3.0 (1.2-4.9) |
| São Paulo | 1.5 (0.6-2.5) | 1.3 (0.4-2.2) | 0.4 (-0.0-0.8) | 1.9 (0.7-3.0) | 1.8 (0.6-3.0) | 2.5 (1.2-3.7) | 1.4 (0.5-2.3) | 3.4 (1.7-5.0) | 2.4 (0.5-4.3) | 2.7 (1.3-4.1) | 1.5 (0.5-2.6) |
| Teresina | 4.3 (2.3-6.2) | 5.1 (3.1-7.2) | 3.3 (1.7-4.9) | 3.5 (1.4-5.5) | 3.0 (1.5-4.4) | 4.8 (2.6-7.0) | 4.9 (2.1-7.7) | 1.9 (0.3-3.5) | 3.1 (0.7-5.6) | 3.0 (1.2-4.8) | 3.3 (1.7-4.9) |
| Vitória | 2.1 (1.1-3.1) | 2.3 (1.1-3.5) | 2.5 (1.1-3.9) | 1.5 (0.6-2.5) | 1.9 (0.7-3.2) | 3.5 (1.2-5.9) | 2.1 (0.5-3.7) | 0.9 (0.2-1.7) | 3.3 (1.4-5.2) | 3.0 (0.9-5.1) | 2.2 (0.5-3.9) |
| State capitals overall | 2.3 (1.9-2.7) | 2.2 (1.9-2.6) | 2.0 (1.7-2.4) | 2.3 (1.9-2.7) | 2.6 (2.1-3.1) | 2.4 (2.0-2.8) | 2.1 (1.8-2.5) | 2.9 (2.4-3.4) | 2.4 (1.9-3.0) | 2.5 (2.0-3.1) | 2.0 (1.6-2.4) |
