# Supplementary material for: Adult body weight trends in 27 urban populations of Brazil from 2006 to 2016: A population-based study
Source: PLoS One. 2019 Mar 6;14(3):e0213254. doi: 10.1371/journal.pone.0213254 (PMC6402686; doi:10.1371/journal.pone.0213254)
Supplement: S4 Table — Numbers in brackets show 95% confidence intervals. (PDF) [file pone.0213254.s004.pdf]

S4 Table. Age-standardized prevalence (%) of underweight (BMI < 18.5 kg/m²) in Brazil's state capitals, from 2006 to 2016, among women. Numbers in brackets show 95% confidence intervals.

State capital	2006	2007	2008	2009	2010	2011	2012	2013	2014	2015	2016
Aracaju	7.7 (5.9-9.5)	6.7 (5.0-8.5)	8.0 (6.0-10.1)	6.8 (4.9-8.6)	8.5 (6.6-10.5)	6.2 (4.5-7.9)	5.1 (3.3-6.9)	5.7 (3.9-7.6)	4.3 (2.8-5.7)	4.9 (3.3-6.5)	4.2 (2.6-5.8)
Belém	7.7 (5.9-9.5)	8.2 (6.3-10.1)	6.3 (4.5-8.0)	7.0 (5.1-9.0)	7.0 (5.2-8.8)	5.4 (3.9-6.8)	6.0 (4.1-7.9)	4.8 (3.2-6.4)	4.9 (3.2-6.7)	5.5 (3.9-7.1)	4.5 (2.9-6.0)
Belo Horizonte	8.0 (6.2-9.8)	8.9 (6.8-11.0)	6.3 (4.6-8.0)	6.7 (5.0-8.5)	6.5 (4.8-8.3)	5.6 (3.9-7.4)	5.1 (3.4-6.7)	4.4 (2.7-6.0)	5.9 (3.8-8.0)	3.9 (2.3-5.5)	4.5 (2.6-6.3)
Boa Vista	8.9 (6.8-11.0)	7.1 (5.3-8.8)	5.1 (3.3-7.0)	5.8 (4.1-7.5)	5.0 (3.6-6.5)	5.0 (3.1-7.0)	4.5 (2.8-6.1)	4.3 (2.9-5.7)	4.3 (2.5-6.2)	5.8 (3.2-8.3)	4.2 (2.7-5.7)
Campo Grande	5.7 (4.0-7.4)	7.1 (4.8-9.3)	4.8 (3.3-6.3)	4.9 (3.4-6.5)	4.8 (3.4-6.2)	4.8 (3.3-6.3)	4.3 (2.7-6.0)	3.3 (1.9-4.7)	4.2 (2.3-6.0)	3.9 (2.4-5.4)	3.0 (1.5-4.5)
Cuiabá	5.6 (4.1-7.2)	6.3 (4.6-8.0)	6.0 (4.2-7.7)	6.0 (4.2-7.9)	6.7 (4.8-8.5)	6.0 (4.3-7.7)	4.8 (2.6-7.0)	7.5 (5.3-9.7)	4.8 (2.8-6.9)	5.0 (3.0-7.0)	3.8 (2.2-5.3)
Curitiba	3.4 (2.2-4.7)	5.6 (4.0-7.2)	4.2 (2.8-5.5)	3.4 (2.0-4.7)	4.6 (3.0-6.2)	3.6 (2.1-5.0)	5.7 (3.5-7.8)	3.1 (1.6-4.6)	2.6 (1.0-4.2)	3.4 (1.8-4.9)	4.8 (2.5-7.0)
Federal District	7.0 (5.1-9.0)	5.8 (4.2-7.4)	6.5 (4.8-8.2)	5.5 (3.1-7.8)	4.6 (2.3-6.9)	4.5 (3.0-5.9)	4.6 (3.1-6.2)	4.9 (3.4-6.5)	4.0 (2.4-5.6)	5.2 (2.6-7.8)	5.2 (2.7-7.8)
Florianópolis	5.2 (3.7-6.7)	7.0 (5.0-9.0)	5.5 (3.9-7.1)	3.7 (2.3-5.0)	3.6 (2.2-5.0)	3.6 (2.1-5.1)	3.8 (2.0-5.6)	3.0 (1.6-4.5)	3.9 (2.0-5.8)	4.2 (1.9-6.6)	5.4 (2.9-7.9)
Fortaleza	7.3 (5.5-9.1)	7.3 (4.9-9.6)	8.2 (6.0-10.5)	5.2 (3.4-7.1)	5.2 (3.5-6.8)	4.4 (2.7-6.2)	5.6 (3.7-7.5)	3.6 (2.3-4.9)	5.0 (3.2-6.7)	3.6 (2.2-5.0)	4.1 (2.3-5.9)
Goiânia	8.9 (6.9-10.9)	7.2 (5.5-8.9)	8.3 (6.3-10.3)	6.4 (4.7-8.2)	7.8 (6.0-9.6)	6.6 (4.9-8.3)	3.9 (2.4-5.3)	5.1 (3.5-6.7)	5.1 (2.9-7.2)	3.4 (1.7-5.1)	3.9 (2.3-5.4)
João Pessoa	6.1 (4.3-7.8)	7.1 (5.3-9.0)	5.4 (3.7-7.1)	5.1 (3.5-6.7)	6.3 (4.5-8.1)	5.2 (3.3-7.0)	3.9 (2.5-5.3)	5.9 (4.0-7.8)	4.1 (2.4-5.7)	4.4 (2.7-6.2)	4.6 (2.8-6.4)
Macapá	10.4 (7.8-12.9)	8.3 (6.3-10.3)	7.1 (5.3-8.9)	8.3 (6.2-10.3)	9.5 (6.8-12.2)	7.9 (5.7-10.1)	6.9 (5.0-8.9)	6.5 (4.4-8.6)	6.0 (3.9-8.0)	5.2 (3.6-6.8)	7.0 (4.0-10.0)

Maceió	10.5 (8.2-12.8)	8.7 (6.6-10.9)	6.1 (4.4-7.8)	8.3 (6.1-10.5)	8.2 (6.0-10.4)	7.6 (5.6-9.6)	6.6 (3.8-9.3)	8.8 (6.3-11.3)	3.4 (1.6-5.3)	4.8 (3.0-6.6)	3.7 (2.2-5.2)
Manaus	6.4 (4.7-8.1)	7.7 (5.7-9.7)	6.9 (5.0-8.8)	5.9 (4.3-7.5)	6.6 (4.7-8.4)	5.4 (3.7-7.2)	5.9 (3.5-8.3)	4.5 (2.7-6.2)	4.0 (2.2-5.8)	3.6 (2.1-5.1)	5.0 (3.0-7.0)
Natal	5.4 (3.9-6.9)	5.5 (3.7-7.3)	5.6 (3.9-7.4)	6.0 (4.2-7.8)	6.1 (4.4-7.8)	5.0 (3.4-6.7)	4.2 (2.7-5.7)	4.7 (3.0-6.3)	2.7 (1.3-4.1)	3.9 (2.5-5.3)	3.7 (2.2-5.2)
Palmas	7.6 (5.3-9.8)	11.4 (8.5-14.3)	6.1 (4.3-7.8)	9.6 (7.4-11.9)	6.9 (5.0-8.8)	6.5 (4.7-8.2)	5.7 (3.9-7.5)	4.1 (2.6-5.6)	6.9 (4.8-8.9)	4.7 (3.0-6.3)	5.0 (3.2-6.9)
Porto Alegre	4.0 (2.4-5.5)	3.2 (1.7-4.6)	2.6 (1.6-3.6)	3.3 (1.6-5.0)	2.7 (1.5-3.9)	3.5 (2.0-4.9)	3.4 (1.8-5.0)	2.6 (1.3-3.9)	2.9 (1.1-4.8)	2.4 (0.4-4.5)	3.3 (1.6-5.0)
Porto Velho	8.2 (6.1-10.3)	7.6 (5.6-9.6)	4.8 (3.3-6.3)	7.4 (5.3-9.6)	6.8 (4.7-8.9)	6.7 (4.8-8.6)	5.8 (3.9-7.7)	4.5 (3.0-6.0)	5.5 (3.4-7.5)	3.4 (1.9-4.9)	5.4 (3.5-7.2)
Recife	7.5 (5.6-9.3)	6.4 (4.6-8.1)	7.0 (5.1-8.8)	8.8 (6.4-11.2)	7.0 (5.1-8.8)	5.3 (3.8-6.9)	5.5 (3.6-7.5)	4.1 (2.6-5.6)	6.2 (4.2-8.3)	4.2 (2.7-5.8)	3.8 (2.4-5.3)
Rio Branco	5.7 (4.1-7.3)	6.5 (4.6-8.4)	5.4 (3.8-7.0)	6.5 (4.7-8.3)	5.6 (4.0-7.2)	5.9 (4.1-7.6)	6.6 (4.6-8.7)	4.1 (2.7-5.6)	4.4 (2.6-6.2)	4.5 (2.4-6.6)	3.8 (2.4-5.1)
Rio de Janeiro	6.6 (4.8-8.4)	7.6 (5.6-9.6)	5.6 (3.9-7.3)	5.3 (3.5-7.1)	6.1 (4.3-8.0)	3.2 (2.0-4.4)	4.5 (2.5-6.5)	5.6 (3.6-7.5)	3.8 (1.9-5.7)	4.3 (2.3-6.3)	4.5 (2.4-6.6)
Salvador	8.3 (6.4-10.1)	8.5 (6.4-10.6)	9.8 (7.7-12.0)	8.1 (6.2-9.9)	6.9 (5.2-8.6)	5.7 (4.0-7.4)	6.2 (4.4-8.1)	6.0 (4.0-7.9)	7.1 (5.0-9.3)	4.2 (2.4-6.0)	5.5 (3.6-7.4)
São Luís	12.3 (10.1-14.5)	10.2 (8.2-12.2)	10.5 (8.3-12.6)	7.7 (6.0-9.4)	6.6 (4.9-8.3)	7.0 (5.2-8.8)	8.3 (6.3-10.4)	7.6 (5.7-9.5)	6.8 (4.8-8.9)	6.4 (4.6-8.2)	4.0 (2.6-5.4)
São Paulo	5.4 (3.8-7.0)	5.6 (4.0-7.2)	6.3 (4.5-8.1)	5.5 (3.9-7.1)	4.2 (2.9-5.6)	4.2 (2.8-5.5)	4.6 (3.0-6.3)	5.2 (3.4-7.0)	4.6 (2.8-6.3)	2.4 (1.3-3.5)	3.4 (2.1-4.7)
Teresina	9.6 (7.6-11.5)	9.1 (7.0-11.3)	8.5 (6.6-10.5)	7.9 (6.1-9.8)	7.9 (5.9-9.9)	6.7 (4.8-8.5)	7.5 (5.0-10.0)	6.1 (4.3-8.0)	5.4 (3.5-7.3)	5.4 (3.8-7.0)	6.3 (4.2-8.3)
Vitória	6.5 (4.9-8.2)	5.4 (3.8-7.0)	3.8 (2.6-5.0)	5.7 (4.0-7.3)	4.5 (3.1-5.9)	4.6 (3.1-6.1)	5.4 (3.5-7.2)	3.2 (1.8-4.5)	5.4 (3.3-7.6)	3.7 (1.9-5.6)	5.7 (3.6-7.7)
State capitals overall	6.7 (6.1-7.2)	6.8 (6.3-7.4)	6.6 (6.0-7.1)	5.9 (5.4-6.5)	5.6 (5.1-6.1)	4.8 (4.3-5.2)	5.1 (4.5-5.6)	5.0 (4.4-5.6)	4.7 (4.1-5.2)	3.8 (3.4-4.3)	4.2 (3.7-4.7)
