# Supplementary material for: Adult body weight trends in 27 urban populations of Brazil from 2006 to 2016: A population-based study
Source: PLoS One. 2019 Mar 6;14(3):e0213254. doi: 10.1371/journal.pone.0213254 (PMC6402686; doi:10.1371/journal.pone.0213254)
Supplement: S5 Table — Numbers in brackets show 95% confidence intervals. (PDF) [file pone.0213254.s005.pdf]

S5 Table. Age-standardized prevalence (%) of normal weight (18.5 kg/m² ≤ BMI < 25 kg/m²) in Brazil's state capitals, from 2006 to 2016, among men. Numbers in brackets show 95% confidence intervals.

State capital	2006	2007	2008	2009	2010	2011	2012	2013	2014	2015	2016
Aracaju	48.5 (43.9-53.1)	54.2 (49.6-58.8)	47.1 (42.4-51.8)	42.8 (38.2-47.3)	43.7 (38.9-48.5)	48.1 (43.6-52.6)	38.2 (33.5-42.9)	45.8 (40.8-50.8)	41.4 (36.4-46.4)	42.6 (38.0-47.2)	38.8 (34.3-43.2)
Belém	47.1 (42.7-51.4)	43.7 (39.5-47.9)	44.6 (40.4-48.8)	45.3 (40.9-49.7)	42.3 (37.7-47.0)	44.2 (39.6-48.9)	40.3 (35.7-44.9)	41.9 (37.3-46.5)	36.6 (31.1-42.1)	36.4 (32.3-40.4)	37.7 (33.1-42.3)
Belo Horizonte	52.7 (48.8-56.6)	49.9 (45.8-54.1)	50.4 (46.4-54.4)	48.8 (44.7-52.8)	49.9 (45.8-54.0)	47.0 (43.1-50.9)	46.0 (41.7-50.3)	48.8 (44.6-53.0)	48.2 (43.3-53.1)	43.9 (39.6-48.2)	45.1 (40.9-49.4)
Boa Vista	46.7 (42.3-51.1)	48.0 (43.2-52.7)	50.0 (45.2-54.8)	42.0 (37.2-46.7)	44.4 (40.1-48.8)	39.4 (34.7-44.1)	41.8 (36.9-46.7)	42.3 (37.2-47.4)	38.2 (33.1-43.2)	34.2 (29.0-39.5)	37.8 (33.1-42.4)
Campo Grande	46.3 (42.2-50.4)	46.5 (42.2-50.8)	45.0 (40.7-49.4)	45.9 (41.7-50.2)	43.6 (39.6-47.6)	42.2 (38.3-46.1)	35.2 (30.9-39.4)	42.9 (37.9-47.9)	40.4 (35.0-45.8)	38.8 (34.5-43.0)	35.4 (30.7-40.1)
Cuiabá	47.4 (43.0-51.8)	41.5 (37.5-45.6)	45.3 (41.0-49.6)	44.4 (40.3-48.5)	43.0 (38.8-47.2)	39.2 (35.2-43.3)	39.3 (34.8-43.8)	39.2 (34.6-43.7)	38.7 (33.8-43.7)	38.3 (32.5-44.1)	34.8 (30.7-38.8)
Curitiba	46.9 (43.1-50.7)	49.7 (45.9-53.6)	46.7 (43.0-50.4)	46.3 (42.3-50.3)	42.5 (38.6-46.4)	41.6 (37.9-45.4)	42.1 (37.7-46.5)	41.7 (37.1-46.3)	40.6 (35.3-45.9)	41.5 (37.5-45.5)	38.8 (33.7-43.9)
Federal District	47.5 (42.1-52.9)	54.4 (50.3-58.6)	49.4 (45.2-53.5)	55.6 (49.5-61.7)	40.5 (33.3-47.7)	44.4 (40.4-48.4)	46.4 (42.1-50.7)	42.9 (38.6-47.2)	40.7 (35.7-45.7)	41.9 (35.6-48.3)	44.8 (39.1-50.6)
Florianópolis	46.8 (42.8-50.9)	46.7 (42.6-50.8)	49.7 (45.7-53.6)	45.1 (41.3-49.0)	46.8 (42.7-50.8)	42.0 (37.9-46.0)	47.3 (42.8-51.7)	41.3 (36.9-45.8)	39.8 (34.7-44.8)	35.4 (30.9-39.9)	40.3 (35.0-45.7)
Fortaleza	47.7 (43.2-52.2)	47.0 (42.4-51.6)	45.4 (40.8-50.0)	46.8 (41.8-51.7)	40.6 (36.1-45.1)	40.5 (36.3-44.8)	41.3 (36.4-46.1)	42.1 (37.7-46.4)	34.8 (29.9-39.8)	35.4 (31.3-39.4)	36.9 (32.2-41.5)
Goiânia	51.7 (47.8-55.7)	51.8 (47.9-55.7)	48.8 (44.9-52.8)	49.1 (45.0-53.1)	44.1 (40.2-48.0)	45.4 (41.5-49.4)	44.5 (40.3-48.6)	45.6 (41.4-49.7)	43.3 (38.8-47.8)	42.0 (35.0-48.9)	45.7 (40.9-50.5)
João Pessoa	46.6 (42.4-50.9)	45.0 (40.4-49.6)	45.5 (40.7-50.4)	49.9 (44.8-54.9)	45.5 (40.1-50.8)	39.7 (35.2-44.2)	40.4 (35.0-45.8)	37.4 (32.4-42.4)	40.3 (35.2-45.4)	43.6 (39.0-48.2)	38.8 (33.5-44.1)
Macapá	47.3 (42.9-51.7)	46.7 (42.0-51.3)	42.1 (37.3-46.9)	45.4 (40.3-50.6)	41.6 (36.8-46.3)	40.5 (35.7-45.3)	43.3 (38.1-48.5)	37.2 (31.5-43.0)	40.8 (35.3-46.4)	40.5 (35.4-45.6)	38.2 (32.4-43.9)

Maceió	52.5 (48.0-56.9)	47.7 (42.5-53.0)	47.3 (42.1-52.5)	49.0 (44.0-54.1)	43.5 (38.5-48.5)	39.6 (35.1-44.2)	41.3 (36.1-46.6)	37.9 (33.1-42.6)	45.4 (39.8-51.0)	42.9 (38.4-47.5)	40.1 (35.3-45.0)
Manaus	43.3 (39.1-47.5)	43.9 (39.8-48.1)	48.7 (44.4-53.0)	45.5 (40.8-50.2)	42.5 (38.3-46.7)	41.4 (37.2-45.6)	42.7 (37.5-48.0)	40.2 (35.5-44.9)	37.2 (32.2-42.2)	30.0 (25.2-34.9)	36.9 (32.0-41.8)
Natal	47.3 (43.0-51.6)	47.2 (42.7-51.8)	48.6 (43.9-53.3)	46.9 (42.4-51.3)	48.3 (43.6-53.1)	42.5 (38.0-47.0)	40.6 (35.7-45.4)	40.9 (36.3-45.5)	42.6 (36.9-48.3)	39.3 (34.6-43.9)	36.5 (31.9-41.1)
Palmas	48.4 (43.8-52.9)	53.4 (48.5-58.3)	49.1 (44.2-54.1)	47.7 (43.3-52.1)	45.8 (41.1-50.5)	48.3 (43.8-52.8)	42.4 (37.5-47.3)	39.4 (33.8-45.0)	41.7 (36.8-46.6)	41.6 (37.0-46.2)	43.1 (39.0-47.2)
Porto Alegre	41.5 (37.4-45.7)	49.0 (44.7-53.4)	43.6 (39.5-47.7)	50.4 (46.3-54.4)	42.5 (38.1-46.9)	40.0 (35.8-44.3)	38.6 (33.8-43.5)	37.0 (32.4-41.6)	34.3 (29.1-39.6)	34.4 (30.0-38.7)	36.3 (31.8-40.9)
Porto Velho	49.7 (45.5-53.9)	47.0 (42.5-51.6)	48.1 (43.6-52.6)	39.5 (35.2-43.7)	44.4 (40.2-48.7)	44.2 (39.8-48.5)	40.6 (35.9-45.4)	38.8 (34.4-43.2)	34.7 (29.5-39.9)	42.8 (37.6-48.0)	36.9 (31.6-42.2)
Recife	48.5 (44.0-53.1)	49.0 (44.4-53.5)	47.3 (42.5-52.0)	43.8 (39.5-48.2)	41.3 (36.9-45.7)	43.7 (39.2-48.3)	42.4 (37.8-47.0)	43.5 (38.8-48.2)	43.0 (37.9-48.1)	44.9 (40.5-49.2)	39.2 (34.7-43.7)
Rio Branco	48.6 (44.3-53.0)	50.0 (44.9-55.1)	41.9 (36.9-47.0)	43.7 (38.7-48.7)	40.8 (36.1-45.5)	42.1 (37.7-46.6)	39.6 (34.3-44.8)	40.6 (35.2-46.0)	37.1 (31.3-43.0)	39.4 (34.3-44.5)	32.9 (28.9-36.8)
Rio de Janeiro	47.1 (42.9-51.2)	46.2 (42.0-50.3)	46.8 (42.6-51.1)	45.3 (40.9-49.8)	43.8 (39.6-48.1)	40.6 (36.3-44.9)	44.0 (39.2-48.7)	39.3 (35.0-43.7)	39.5 (34.4-44.7)	40.5 (35.1-45.9)	39.5 (34.4-44.7)
Salvador	53.5 (49.5-57.6)	50.1 (45.8-54.4)	53.8 (49.6-58.0)	48.1 (43.8-52.3)	55.1 (50.9-59.3)	52.1 (48.0-56.2)	51.7 (47.1-56.3)	47.0 (42.8-51.3)	42.8 (38.0-47.6)	40.9 (36.3-45.5)	44.0 (39.5-48.6)
São Luís	53.9 (49.6-58.2)	49.9 (45.4-54.4)	48.0 (43.2-52.7)	47.8 (43.5-52.1)	49.8 (45.5-54.1)	50.6 (46.0-55.2)	44.5 (39.4-49.6)	49.0 (44.2-53.8)	44.4 (39.2-49.5)	42.5 (37.8-47.3)	45.3 (40.4-50.3)
São Paulo	53.0 (49.0-56.9)	49.4 (45.4-53.5)	46.7 (42.6-50.7)	47.2 (42.9-51.5)	45.0 (40.9-49.0)	45.8 (41.9-49.7)	43.6 (39.2-48.0)	42.3 (38.3-46.2)	42.3 (37.5-47.1)	38.8 (34.8-42.9)	41.0 (37.0-44.9)
Teresina	50.3 (46.0-54.6)	49.7 (45.2-54.3)	54.2 (49.7-58.6)	51.4 (46.6-56.1)	42.9 (37.7-48.1)	43.6 (39.1-48.1)	41.5 (36.2-46.9)	43.3 (38.3-48.2)	44.4 (38.8-50.0)	42.4 (37.8-46.9)	38.1 (33.5-42.8)
Vitória	50.7 (46.7-54.6)	45.3 (41.4-49.3)	47.3 (43.3-51.3)	48.3 (44.2-52.3)	43.3 (39.1-47.4)	45.6 (41.5-49.6)	42.4 (38.0-46.8)	46.1 (41.7-50.6)	42.3 (37.5-47.1)	43.7 (38.5-49.0)	43.5 (38.7-48.3)
State capitals overall	49.6 (48.2-50.9)	48.4 (47.1-49.8)	47.6 (46.2-48.9)	47.3 (45.8-48.7)	44.7 (43.3-46.1)	44.0 (42.7-45.3)	43.5 (42.0-44.9)	42.3 (40.9-43.6)	41.1 (39.5-42.6)	39.8 (38.3-41.2)	40.5 (39.0-41.9)
