# Supplementary material for: Adult body weight trends in 27 urban populations of Brazil from 2006 to 2016: A population-based study
Source: PLoS One. 2019 Mar 6;14(3):e0213254. doi: 10.1371/journal.pone.0213254 (PMC6402686; doi:10.1371/journal.pone.0213254)
Supplement: S6 Table — Numbers in brackets show 95% confidence intervals. (PDF) [file pone.0213254.s006.pdf]

**S6 Table. Age-standardized prevalence (%) of normal weight (18.5 kg/m² ≤ BMI < 25 kg/m²) in Brazil's state capitals, from 2006 to 2016, among women.** Numbers in brackets show 95% confidence intervals.

| State capital | 2006 | 2007 | 2008 | 2009 | 2010 | 2011 | 2012 | 2013 | 2014 | 2015 | 2016 |
|---|---|---|---|---|---|---|---|---|---|---|---|
| Aracaju | 56.4 (53.3-59.5) | 57.0 (53.7-60.3) | 50.6 (47.1-54.1) | 52.5 (49.0-56.0) | 45.5 (42.1-48.9) | 50.6 (47.3-53.9) | 51.1 (47.4-54.8) | 47.9 (44.3-51.4) | 49.7 (45.9-53.4) | 48.3 (45.0-51.6) | 43.4 (39.7-47.0) |
| Belém | 58.3 (55.1-61.4) | 56.6 (53.1-60.1) | 54.1 (50.5-57.7) | 56.1 (52.6-59.5) | 52.8 (49.4-56.1) | 51.5 (48.1-54.8) | 50.5 (46.6-54.3) | 48.8 (45.2-52.4) | 48.6 (44.5-52.8) | 46.5 (43.1-49.9) | 49.0 (45.3-52.7) |
| Belo Horizonte | 59.7 (56.6-62.9) | 56.9 (53.6-60.1) | 56.2 (52.9-59.4) | 54.7 (51.4-57.9) | 55.4 (52.1-58.6) | 54.2 (50.8-57.6) | 52.1 (48.7-55.6) | 51.8 (48.2-55.4) | 48.6 (44.6-52.6) | 52.6 (49.1-56.0) | 52.7 (48.9-56.4) |
| Boa Vista | 54.4 (50.9-57.8) | 55.4 (51.9-58.8) | 52.2 (48.5-55.9) | 50.1 (46.4-53.7) | 47.5 (44.0-51.1) | 50.7 (46.9-54.5) | 51.3 (47.4-55.2) | 48.4 (44.7-52.1) | 50.4 (46.0-54.8) | 40.2 (36.2-44.3) | 49.1 (45.2-53.1) |
| Campo Grande | 56.7 (53.5-59.9) | 53.5 (50.0-57.0) | 55.8 (52.5-59.0) | 50.0 (46.7-53.3) | 47.7 (44.6-50.9) | 50.5 (47.2-53.8) | 44.9 (41.2-48.7) | 48.2 (44.4-52.1) | 47.3 (42.7-51.9) | 40.0 (36.3-43.7) | 45.1 (40.8-49.3) |
| Cuiabá | 55.9 (52.4-59.3) | 51.8 (48.4-55.2) | 48.9 (45.5-52.3) | 52.3 (48.8-55.8) | 47.2 (43.9-50.5) | 48.5 (45.2-51.9) | 49.9 (46.1-53.8) | 42.4 (38.9-46.0) | 44.4 (40.3-48.4) | 47.9 (43.3-52.5) | 46.9 (43.2-50.5) |
| Curitiba | 60.4 (57.5-63.3) | 55.3 (52.2-58.5) | 54.5 (51.4-57.7) | 56.7 (53.5-59.9) | 54.1 (50.8-57.3) | 54.1 (50.8-57.3) | 48.4 (44.6-52.2) | 51.3 (47.4-55.2) | 47.4 (42.6-52.1) | 48.2 (44.4-52.0) | 48.6 (43.8-53.4) |
| Federal District | 58.6 (54.9-62.3) | 55.7 (52.4-59.0) | 56.1 (53.0-59.3) | 59.4 (54.9-64.0) | 56.4 (49.8-62.9) | 51.3 (48.0-54.6) | 51.0 (47.5-54.4) | 51.8 (48.4-55.3) | 52.3 (48.3-56.3) | 52.0 (46.9-57.1) | 49.4 (44.5-54.2) |
| Florianópolis | 62.4 (59.3-65.5) | 59.6 (56.2-63.0) | 59.4 (56.1-62.6) | 58.2 (54.8-61.6) | 57.9 (54.7-61.1) | 59.0 (55.6-62.4) | 50.9 (46.7-55.1) | 58.4 (54.6-62.2) | 55.2 (50.8-59.6) | 56.0 (51.7-60.3) | 55.8 (51.2-60.5) |
| Fortaleza | 57.8 (54.4-61.2) | 54.0 (50.4-57.7) | 50.9 (47.3-54.5) | 50.2 (46.8-53.7) | 45.7 (42.2-49.1) | 49.4 (46.0-52.8) | 45.1 (41.3-48.9) | 49.4 (45.9-52.9) | 46.2 (42.2-50.1) | 48.1 (44.5-51.6) | 44.4 (40.5-48.3) |
| Goiânia | 58.3 (55.2-61.5) | 58.6 (55.5-61.7) | 55.1 (51.8-58.4) | 54.6 (51.3-57.9) | 54.3 (51.1-57.6) | 52.9 (49.7-56.1) | 50.4 (46.9-53.8) | 52.0 (48.5-55.5) | 49.4 (45.3-53.6) | 58.6 (53.7-63.5) | 52.0 (48.4-55.6) |
| João Pessoa | 56.5 (53.1-59.9) | 52.9 (49.3-56.5) | 50.3 (46.5-54.1) | 52.1 (48.6-55.5) | 52.7 (48.9-56.5) | 51.6 (47.9-55.3) | 50.1 (46.1-54.0) | 50.1 (46.4-53.8) | 50.6 (46.5-54.7) | 42.7 (38.8-46.5) | 43.1 (38.8-47.4) |
| Macapá | 51.1 (47.6-54.6) | 51.1 (47.4-54.8) | 47.7 (44.1-51.2) | 47.1 (43.3-50.9) | 45.8 (41.9-49.8) | 43.6 (39.8-47.3) | 42.2 (38.3-46.0) | 48.7 (44.8-52.6) | 45.1 (40.9-49.3) | 46.2 (42.3-50.1) | 43.5 (39.3-47.6) |

| | | | | | | | | | | | |
|---|---|---|---|---|---|---|---|---|---|---|---|
| Maceió | 52.6 (49.0-56.1) | 55.1 (51.4-58.7) | 51.5 (47.8-55.3) | 51.5 (47.7-55.2) | 48.7 (45.0-52.4) | 48.0 (44.3-51.6) | 45.8 (41.6-50.0) | 44.9 (41.0-48.8) | 45.0 (40.6-49.5) | 43.9 (40.1-47.7) | 44.3 (40.3-48.3) |
| Manaus | 55.4 (52.2-58.7) | 50.6 (47.1-54.0) | 51.5 (48.0-55.0) | 49.3 (46.0-52.6) | 45.1 (41.8-48.5) | 43.1 (39.8-46.4) | 42.1 (38.1-46.1) | 44.1 (40.3-47.8) | 42.8 (38.4-47.1) | 41.7 (37.5-45.9) | 43.1 (39.2-47.1) |
| Natal | 57.9 (54.6-61.2) | 53.2 (49.8-56.7) | 53.5 (49.9-57.1) | 51.7 (48.3-55.0) | 45.3 (41.9-48.7) | 46.0 (42.6-49.4) | 47.6 (43.8-51.4) | 48.3 (44.5-52.0) | 48.0 (44.0-52.1) | 45.0 (41.2-48.8) | 46.3 (42.4-50.3) |
| Palmas | 59.9 (55.9-63.9) | 54.7 (50.9-58.5) | 61.6 (57.6-65.6) | 55.0 (51.0-58.9) | 54.7 (51.3-58.1) | 54.0 (50.3-57.6) | 53.2 (49.3-57.1) | 52.5 (48.5-56.5) | 47.2 (43.1-51.2) | 51.2 (47.3-55.1) | 52.5 (48.8-56.3) |
| Porto Alegre | 56.8 (53.4-60.1) | 59.4 (56.1-62.7) | 57.3 (53.8-60.8) | 54.0 (50.3-57.7) | 50.1 (46.6-53.6) | 48.8 (45.2-52.5) | 48.9 (44.8-53.1) | 53.1 (48.8-57.4) | 50.5 (45.3-55.6) | 50.1 (45.4-54.9) | 52.0 (47.5-56.6) |
| Porto Velho | 53.9 (50.3-57.5) | 52.4 (48.8-56.1) | 52.1 (48.6-55.6) | 49.2 (45.5-52.8) | 45.6 (42.0-49.1) | 48.1 (44.6-51.6) | 45.8 (42.0-49.6) | 46.7 (42.6-50.7) | 43.9 (39.2-48.6) | 43.5 (39.4-47.6) | 44.6 (40.5-48.6) |
| Recife | 52.5 (49.2-55.9) | 52.6 (49.1-56.2) | 53.3 (49.9-56.8) | 49.6 (45.9-53.3) | 48.9 (45.4-52.3) | 50.7 (47.3-54.1) | 44.2 (40.2-48.2) | 49.0 (45.2-52.7) | 40.8 (36.8-44.7) | 44.5 (40.9-48.0) | 45.3 (41.5-49.2) |
| Rio Branco | 50.7 (47.2-54.2) | 52.8 (49.2-56.4) | 50.1 (46.2-53.9) | 47.7 (44.0-51.4) | 47.4 (43.8-50.9) | 45.6 (42.1-49.1) | 42.6 (38.7-46.6) | 45.7 (41.6-49.8) | 45.4 (40.9-49.9) | 41.2 (37.1-45.3) | 40.3 (36.8-43.7) |
| Rio de Janeiro | 52.4 (49.2-55.6) | 52.6 (49.1-56.0) | 53.9 (50.5-57.3) | 52.1 (48.7-55.6) | 47.6 (44.0-51.1) | 54.4 (51.0-57.8) | 48.6 (44.6-52.6) | 50.3 (46.6-53.9) | 48.5 (44.1-53.0) | 43.6 (38.9-48.3) | 46.1 (41.5-50.6) |
| Salvador | 52.1 (48.8-55.4) | 52.4 (49.1-55.8) | 49.2 (45.9-52.6) | 49.1 (45.9-52.3) | 51.4 (48.3-54.6) | 50.0 (46.6-53.4) | 45.8 (42.2-49.4) | 50.4 (46.9-53.9) | 45.4 (41.4-49.3) | 46.9 (43.1-50.7) | 43.7 (40.0-47.4) |
| São Luís | 55.4 (52.1-58.7) | 56.3 (52.9-59.7) | 55.1 (51.8-58.5) | 56.5 (53.1-59.9) | 55.4 (52.2-58.7) | 52.0 (48.6-55.4) | 51.7 (48.1-55.3) | 53.0 (49.5-56.5) | 50.9 (46.9-55.0) | 51.1 (47.7-54.6) | 50.6 (46.7-54.5) |
| São Paulo | 55.0 (51.9-58.1) | 57.2 (54.1-60.4) | 54.4 (51.2-57.5) | 52.8 (49.5-56.2) | 51.9 (48.6-55.2) | 53.6 (50.3-56.8) | 49.6 (46.1-53.2) | 49.3 (45.9-52.6) | 48.7 (44.8-52.5) | 47.4 (43.8-51.0) | 49.0 (45.5-52.5) |
| Teresina | 58.1 (54.6-61.6) | 55.6 (52.1-59.1) | 54.7 (51.1-58.4) | 54.3 (50.9-57.7) | 55.9 (52.4-59.3) | 52.6 (49.0-56.1) | 51.7 (47.7-55.7) | 49.1 (45.4-52.9) | 50.8 (46.6-55.0) | 49.2 (45.4-53.0) | 48.6 (44.7-52.5) |
| Vitória | 60.3 (57.3-63.3) | 62.2 (59.1-65.2) | 58.4 (55.3-61.6) | 55.4 (52.1-58.7) | 57.0 (53.7-60.3) | 53.7 (50.3-57.1) | 54.3 (50.5-58.1) | 53.4 (49.8-57.1) | 47.9 (43.7-52.0) | 51.2 (47.0-55.4) | 51.1 (47.1-55.1) |
| State capitals overall | 55.6 (54.5-56.6) | 55.3 (54.2-56.3) | 53.7 (52.6-54.7) | 52.9 (51.8-54.0) | 51.0 (49.9-52.1) | 51.8 (50.7-52.9) | 48.6 (47.5-49.8) | 49.6 (48.5-50.7) | 48.1 (46.8-49.3) | 47.5 (46.3-48.8) | 47.6 (46.4-48.8) |
