# Supplementary material for: Adult body weight trends in 27 urban populations of Brazil from 2006 to 2016: A population-based study
Source: PLoS One. 2019 Mar 6;14(3):e0213254. doi: 10.1371/journal.pone.0213254 (PMC6402686; doi:10.1371/journal.pone.0213254)
Supplement: S7 Table — Numbers in brackets show 95% confidence intervals. (PDF) [file pone.0213254.s007.pdf]

S7 Table. Age-standardized prevalence (%) of pre-obesity (25 kg/m² ≤ BMI < 30 kg/m²) in Brazil's state capitals, from 2006 to 2016, among men. Numbers in brackets show 95% confidence intervals.

State capital	2006	2007	2008	2009	2010	2011	2012	2013	2014	2015	2016
Aracaju	33.4 (29.1-37.8)	32.9 (28.4-37.3)	35.1 (30.7-39.5)	34.6 (30.3-38.9)	35.6 (31.2-40.1)	34.0 (29.8-38.2)	40.3 (35.6-45.0)	35.2 (30.6-39.7)	36.0 (31.1-40.9)	38.4 (33.8-43.0)	37.6 (33.2-42.0)
Belém	33.3 (29.3-37.2)	35.2 (31.2-39.3)	39.0 (34.8-43.2)	39.6 (35.2-44.0)	35.4 (31.0-39.8)	36.2 (32.1-40.4)	40.1 (35.2-45.1)	39.0 (34.5-43.5)	40.6 (35.3-46.0)	38.5 (34.4-42.6)	39.8 (35.2-44.4)
Belo Horizonte	35.9 (32.1-39.7)	36.6 (32.6-40.5)	35.9 (32.0-39.9)	37.6 (33.6-41.5)	36.5 (32.6-40.3)	37.0 (33.3-40.7)	39.0 (34.8-43.3)	34.1 (30.2-38.0)	33.9 (29.3-38.4)	39.3 (35.0-43.5)	37.8 (33.7-41.8)
Boa Vista	36.3 (32.1-40.5)	35.7 (31.2-40.1)	32.9 (28.5-37.2)	40.1 (35.4-44.9)	38.4 (34.2-42.7)	41.7 (36.7-46.7)	38.8 (33.9-43.7)	35.6 (30.7-40.5)	38.4 (33.3-43.4)	39.6 (34.2-45.1)	38.6 (33.7-43.4)
Campo Grande	36.5 (32.5-40.4)	35.1 (31.0-39.1)	40.0 (35.7-44.3)	36.3 (32.2-40.3)	38.9 (35.1-42.8)	37.9 (34.0-41.8)	41.6 (37.2-46.0)	40.4 (35.6-45.2)	39.6 (34.5-44.7)	39.5 (35.4-43.7)	41.5 (36.5-46.4)
Cuiabá	36.9 (32.7-41.0)	40.9 (36.7-45.1)	37.0 (32.8-41.1)	39.0 (34.9-43.1)	34.8 (30.7-38.9)	37.3 (33.3-41.2)	38.4 (34.1-42.8)	37.2 (32.9-41.6)	39.2 (34.3-44.0)	43.4 (37.3-49.5)	39.2 (34.9-43.4)
Curitiba	38.9 (35.2-42.6)	36.3 (32.5-40.1)	37.0 (33.4-40.7)	39.1 (35.1-43.0)	39.3 (35.4-43.2)	41.4 (37.7-45.2)	39.5 (35.2-43.8)	38.4 (34.0-42.8)	38.3 (33.1-43.5)	43.6 (39.6-47.6)	38.1 (33.3-43.0)
Federal District	39.6 (34.2-45.0)	33.9 (30.0-37.9)	37.9 (34.0-41.8)	34.7 (29.0-40.4)	41.9 (34.6-49.2)	40.0 (36.2-43.8)	36.5 (32.5-40.5)	38.9 (34.7-43.2)	40.8 (35.8-45.8)	39.0 (33.2-44.9)	34.9 (29.4-40.3)
Florianópolis	41.3 (37.2-45.3)	41.4 (37.3-45.4)	36.3 (32.5-40.1)	40.0 (36.2-43.9)	36.3 (32.5-40.2)	39.4 (35.5-43.3)	34.7 (30.5-38.9)	40.4 (35.9-44.8)	43.6 (38.4-48.8)	44.8 (39.7-49.8)	42.7 (37.5-47.9)
Fortaleza	35.1 (30.9-39.2)	35.7 (31.4-40.0)	35.3 (30.9-39.7)	35.0 (30.5-39.6)	35.9 (31.4-40.3)	37.5 (33.3-41.8)	37.6 (33.0-42.2)	35.1 (31.0-39.2)	41.8 (36.9-46.7)	41.4 (37.1-45.6)	41.1 (36.4-45.8)
Goiânia	34.9 (31.1-38.7)	33.5 (29.8-37.2)	38.3 (34.5-42.2)	38.1 (34.2-42.1)	39.7 (35.8-43.5)	39.7 (35.8-43.5)	40.3 (36.2-44.4)	33.5 (29.6-37.5)	38.8 (34.5-43.2)	40.4 (33.8-47.1)	33.6 (29.3-38.0)
João Pessoa	33.1 (29.2-37.0)	38.0 (33.5-42.6)	35.0 (30.5-39.5)	35.9 (31.2-40.7)	37.0 (31.7-42.2)	39.8 (35.3-44.2)	34.5 (29.6-39.4)	43.8 (38.6-49.1)	40.4 (35.2-45.5)	35.8 (31.3-40.3)	36.0 (30.7-41.2)
Macapá	32.4 (28.3-36.4)	30.6 (26.4-34.8)	40.8 (36.1-45.5)	33.7 (29.1-38.4)	38.8 (34.0-43.6)	36.5 (31.9-41.1)	38.4 (33.3-43.5)	38.2 (33.0-43.3)	39.4 (34.0-44.8)	34.7 (29.9-39.5)	38.2 (32.9-43.5)

Maceió	31.2 (27.2-35.2)	35.9 (30.7-41.0)	34.9 (30.1-39.8)	34.5 (29.9-39.0)	37.0 (32.2-41.8)	40.8 (36.0-45.6)	38.3 (33.1-43.5)	39.7 (34.8-44.6)	33.1 (27.7-38.4)	36.5 (32.2-40.9)	37.4 (32.6-42.3)
Manaus	39.5 (35.4-43.6)	39.7 (35.7-43.8)	32.6 (28.7-36.5)	35.6 (31.2-40.0)	36.4 (32.4-40.5)	35.6 (31.5-39.8)	35.0 (29.9-40.1)	36.6 (32.0-41.2)	42.7 (37.6-47.7)	38.2 (33.0-43.5)	41.9 (37.0-46.7)
Natal	35.5 (31.5-39.5)	35.2 (30.9-39.5)	35.8 (31.3-40.3)	36.5 (32.1-40.8)	34.6 (30.2-38.9)	36.6 (32.3-41.0)	35.3 (30.7-40.0)	37.8 (33.2-42.5)	37.1 (31.7-42.4)	37.9 (33.3-42.4)	42.6 (38.0-47.3)
Palmas	37.1 (32.6-41.5)	31.5 (27.2-35.7)	35.2 (30.6-39.9)	38.2 (33.9-42.4)	33.9 (29.7-38.1)	33.6 (29.4-37.8)	39.6 (34.9-44.4)	37.1 (31.8-42.3)	37.7 (32.9-42.5)	41.9 (37.4-46.4)	39.3 (35.1-43.5)
Porto Alegre	45.0 (40.7-49.3)	36.4 (32.3-40.5)	40.0 (35.9-44.1)	37.7 (33.8-41.6)	39.9 (35.5-44.3)	42.6 (38.3-46.9)	42.1 (37.1-47.0)	43.4 (38.6-48.3)	38.2 (32.9-43.5)	41.1 (36.3-45.9)	42.8 (38.0-47.6)
Porto Velho	34.6 (30.6-38.6)	35.0 (30.7-39.2)	34.8 (30.6-39.1)	37.6 (33.5-41.8)	38.8 (34.5-43.0)	34.2 (30.2-38.1)	37.6 (32.8-42.4)	39.4 (35.1-43.8)	43.8 (38.3-49.2)	37.1 (32.4-41.7)	39.6 (34.2-44.9)
Recife	35.6 (31.4-39.8)	34.9 (30.5-39.2)	34.9 (30.5-39.2)	36.7 (32.3-41.1)	36.5 (32.3-40.7)	38.8 (34.4-43.2)	37.6 (32.8-42.3)	36.2 (31.7-40.7)	36.9 (32.1-41.6)	37.5 (33.3-41.7)	39.3 (34.8-43.7)
Rio Branco	34.0 (29.9-38.1)	32.8 (28.0-37.7)	41.5 (36.6-46.4)	36.8 (32.0-41.5)	40.5 (35.8-45.2)	38.5 (34.2-42.8)	39.2 (33.9-44.6)	40.8 (35.5-46.1)	38.0 (32.3-43.8)	36.9 (31.8-41.9)	41.2 (37.0-45.3)
Rio de Janeiro	39.5 (35.4-43.6)	37.8 (33.8-41.9)	37.1 (33.1-41.1)	35.3 (31.1-39.5)	39.8 (35.5-44.0)	39.5 (35.2-43.7)	37.4 (32.9-42.0)	36.8 (32.4-41.2)	38.8 (33.7-43.9)	42.6 (37.1-48.2)	38.3 (33.3-43.3)
Salvador	33.7 (29.8-37.5)	33.1 (29.1-37.0)	33.4 (29.5-37.3)	36.2 (32.1-40.3)	32.5 (28.6-36.3)	34.0 (30.1-37.8)	36.3 (31.9-40.7)	36.3 (32.3-40.4)	39.1 (34.3-43.8)	40.6 (36.0-45.1)	35.6 (31.4-39.9)
São Luís	31.7 (27.8-35.6)	34.7 (30.6-38.8)	37.5 (33.1-42.0)	37.1 (32.8-41.4)	33.5 (29.5-37.6)	31.9 (27.9-35.8)	37.2 (32.4-41.9)	34.3 (29.9-38.7)	39.7 (34.7-44.7)	39.7 (35.0-44.4)	38.3 (33.6-43.0)
São Paulo	35.7 (31.8-39.6)	34.0 (30.2-37.8)	37.9 (34.0-41.8)	36.1 (32.0-40.1)	39.6 (35.5-43.6)	37.7 (34.0-41.4)	37.7 (33.6-41.9)	37.3 (33.5-41.1)	38.8 (34.0-43.5)	37.8 (33.7-41.8)	42.4 (38.4-46.4)
Teresina	34.3 (30.3-38.3)	33.4 (29.3-37.5)	30.1 (26.2-34.1)	32.6 (28.2-37.0)	39.5 (34.3-44.7)	38.4 (34.0-42.8)	36.5 (31.3-41.6)	36.9 (31.9-41.8)	36.4 (30.9-41.8)	37.9 (33.5-42.3)	41.1 (36.3-45.8)
Vitória	36.1 (32.4-39.9)	38.5 (34.6-42.4)	38.2 (34.4-42.0)	39.1 (35.1-43.1)	39.7 (35.6-43.8)	38.0 (34.1-41.9)	38.2 (33.8-42.6)	36.8 (32.6-41.1)	37.7 (33.0-42.4)	40.7 (35.5-45.9)	41.9 (37.1-46.7)
State capitals overall	36.5 (35.2-37.8)	35.5 (34.2-36.8)	36.7 (35.4-38.0)	36.4 (35.0-37.7)	38.1 (36.7-39.5)	38.0 (36.8-39.3)	37.9 (36.5-39.3)	37.2 (35.9-38.5)	38.8 (37.3-40.4)	39.5 (38.0-40.9)	39.4 (38.0-40.8)
