# Supplementary material for: Adult body weight trends in 27 urban populations of Brazil from 2006 to 2016: A population-based study
Source: PLoS One. 2019 Mar 6;14(3):e0213254. doi: 10.1371/journal.pone.0213254 (PMC6402686; doi:10.1371/journal.pone.0213254)
Supplement: S8 Table — Numbers in brackets show 95% confidence intervals. (PDF) [file pone.0213254.s008.pdf]

S8 Table. Age-standardized prevalence (%) of pre-obesity (25 kg/m² ≤ BMI < 30 kg/m²) in Brazil's state capitals, from 2006 to 2016, among women. Numbers in brackets show 95% confidence intervals.

State capital	2006	2007	2008	2009	2010	2011	2012	2013	2014	2015	2016
Aracaju	23.2 (20.5-25.8)	23.1 (20.3-25.9)	26.6 (23.5-29.7)	26.3 (23.2-29.5)	30.6 (27.5-33.8)	26.1 (23.2-28.9)	27.3 (24.1-30.6)	28.5 (25.3-31.7)	29.7 (26.2-33.2)	29.6 (26.6-32.6)	32.8 (29.4-36.3)
Belém	23.2 (20.5-25.9)	22.0 (19.1-24.9)	26.2 (23.0-29.4)	22.3 (19.4-25.1)	25.8 (22.9-28.8)	30.1 (27.0-33.1)	28.3 (24.8-31.8)	31.2 (27.8-34.5)	26.5 (22.9-30.0)	30.0 (27.0-33.1)	29.5 (26.1-32.9)
Belo Horizonte	22.8 (20.1-25.5)	22.2 (19.7-24.8)	25.1 (22.4-27.9)	24.9 (22.0-27.8)	25.2 (22.4-28.0)	25.8 (22.9-28.8)	28.0 (24.9-31.1)	29.8 (26.5-33.1)	28.5 (24.9-32.0)	25.6 (22.7-28.6)	26.8 (23.6-30.0)
Boa Vista	24.0 (21.0-27.0)	24.7 (21.6-27.7)	27.8 (24.5-31.1)	29.8 (26.5-33.1)	30.1 (26.7-33.5)	26.6 (23.2-30.1)	27.2 (23.8-30.7)	30.0 (26.6-33.4)	27.9 (24.0-31.7)	32.2 (28.1-36.2)	30.4 (26.8-34.0)
Campo Grande	24.3 (21.6-26.9)	24.7 (21.7-27.7)	25.1 (22.2-28.0)	28.1 (25.2-31.1)	29.3 (26.4-32.3)	27.6 (24.7-30.5)	28.8 (25.4-32.2)	28.5 (25.2-31.9)	24.8 (20.9-28.7)	32.6 (29.1-36.1)	33.9 (29.9-37.8)
Cuiabá	25.9 (22.8-29.0)	27.9 (24.8-31.0)	31.1 (28.0-34.3)	25.8 (22.7-28.9)	27.9 (25.0-30.8)	27.7 (24.8-30.6)	26.7 (23.5-29.8)	27.7 (24.5-30.9)	27.9 (24.2-31.6)	29.3 (25.2-33.4)	29.6 (26.3-32.9)
Curitiba	23.9 (21.3-26.4)	26.2 (23.5-28.8)	28.0 (25.1-30.9)	28.2 (25.4-31.0)	24.6 (21.8-27.4)	27.0 (24.2-29.9)	30.4 (26.9-33.9)	29.8 (26.3-33.4)	30.5 (26.3-34.8)	31.3 (27.8-34.8)	31.1 (26.6-35.7)
Federal District	23.3 (20.1-26.5)	27.5 (24.6-30.5)	22.7 (20.0-25.4)	24.9 (20.9-28.8)	29.5 (22.5-36.5)	29.3 (26.3-32.3)	29.3 (26.0-32.5)	28.8 (25.6-31.9)	28.4 (24.7-32.0)	29.2 (24.5-33.8)	29.1 (24.9-33.3)
Florianópolis	21.9 (19.3-24.6)	22.1 (19.2-25.1)	23.9 (21.1-26.7)	24.2 (21.3-27.1)	25.0 (22.2-27.8)	24.2 (21.3-27.1)	30.5 (26.7-34.3)	25.4 (22.0-28.8)	27.6 (23.8-31.4)	25.3 (21.8-28.8)	24.9 (21.0-28.9)
Fortaleza	24.7 (21.8-27.7)	25.2 (22.2-28.1)	26.5 (23.3-29.7)	28.6 (25.5-31.6)	31.7 (28.5-34.9)	28.9 (25.9-32.0)	30.7 (27.2-34.2)	30.5 (27.2-33.7)	32.2 (28.6-35.8)	30.3 (27.1-33.6)	32.6 (29.0-36.2)
Goiânia	22.4 (19.7-25.0)	22.9 (20.1-25.6)	23.8 (21.1-26.6)	26.8 (23.8-29.7)	25.1 (22.3-27.8)	26.0 (23.2-28.8)	30.2 (27.0-33.4)	28.3 (25.1-31.5)	29.6 (26.2-33.0)	25.8 (21.7-29.9)	29.8 (26.7-33.0)
João Pessoa	24.7 (21.6-27.7)	27.2 (24.1-30.4)	28.8 (25.2-32.4)	26.4 (23.2-29.6)	26.8 (23.5-30.0)	27.2 (23.9-30.4)	27.6 (24.2-31.0)	25.9 (22.8-29.0)	29.0 (25.3-32.7)	31.7 (28.0-35.3)	32.8 (28.8-36.8)
Macapá	24.7 (21.7-27.7)	24.7 (21.5-27.9)	28.5 (25.1-31.9)	28.2 (24.7-31.7)	25.8 (22.5-29.2)	30.2 (26.7-33.7)	29.2 (25.5-33.0)	29.4 (25.9-32.8)	27.0 (23.3-30.6)	29.2 (25.6-32.8)	32.3 (28.6-36.0)

Maceió	23.0 (20.1-26.0)	23.4 (20.2-26.5)	28.3 (24.9-31.7)	25.0 (21.9-28.1)	28.7 (25.4-32.0)	26.8 (23.7-30.0)	27.3 (23.7-30.8)	28.5 (25.1-31.9)	32.0 (27.5-36.5)	30.4 (26.9-34.0)	30.7 (26.7-34.6)
Manaus	22.3 (19.6-24.9)	25.7 (22.5-28.8)	25.1 (22.1-28.0)	28.0 (25.0-31.0)	29.8 (26.7-32.9)	32.3 (29.1-35.5)	31.6 (27.8-35.3)	31.9 (28.3-35.5)	32.3 (28.4-36.3)	30.0 (26.4-33.7)	30.6 (26.8-34.4)
Natal	25.9 (22.9-28.8)	28.3 (25.2-31.4)	27.5 (24.3-30.7)	28.0 (25.0-31.1)	31.0 (27.8-34.2)	33.3 (30.1-36.5)	26.9 (23.6-30.3)	32.9 (29.4-36.5)	29.4 (25.8-33.1)	33.0 (29.3-36.6)	30.2 (26.9-33.5)
Palmas	20.3 (16.9-23.7)	21.7 (18.5-25.0)	21.0 (17.6-24.4)	25.8 (22.3-29.3)	25.0 (21.9-28.1)	24.8 (21.6-27.9)	23.6 (20.5-26.8)	28.6 (24.9-32.4)	29.7 (25.9-33.5)	29.7 (26.0-33.4)	27.1 (24.0-30.2)
Porto Alegre	27.1 (24.1-30.1)	25.1 (22.3-27.9)	26.2 (23.1-29.4)	26.4 (23.2-29.6)	33.0 (29.7-36.2)	28.9 (25.7-32.2)	29.4 (25.6-33.2)	29.3 (25.3-33.4)	28.5 (23.9-33.0)	28.7 (24.5-32.8)	26.7 (22.8-30.6)
Porto Velho	23.1 (20.0-26.1)	22.6 (19.5-25.6)	27.2 (24.0-30.3)	24.7 (21.7-27.6)	29.3 (26.0-32.5)	27.9 (24.8-31.1)	28.6 (25.1-32.0)	31.7 (27.9-35.5)	31.3 (26.5-36.2)	29.8 (26.2-33.4)	29.2 (25.5-32.9)
Recife	25.7 (22.8-28.7)	28.2 (24.9-31.4)	27.0 (23.9-30.0)	28.1 (24.9-31.4)	27.3 (24.3-30.3)	28.0 (24.8-31.1)	33.2 (29.4-37.0)	28.8 (25.4-32.2)	32.6 (28.8-36.3)	31.1 (27.9-34.4)	31.5 (28.0-35.1)
Rio Branco	29.0 (25.7-32.2)	26.6 (23.3-29.8)	25.7 (22.4-29.0)	29.1 (25.8-32.5)	27.6 (24.4-30.8)	28.8 (25.7-31.9)	26.4 (23.0-29.9)	30.0 (26.2-33.8)	32.1 (27.8-36.5)	32.4 (28.4-36.3)	32.6 (29.3-35.9)
Rio de Janeiro	28.2 (25.4-31.0)	26.4 (23.4-29.4)	26.6 (23.6-29.5)	27.3 (24.4-30.3)	29.1 (25.9-32.3)	26.2 (23.3-29.1)	26.9 (23.6-30.2)	26.2 (23.1-29.4)	30.1 (26.1-34.1)	30.3 (26.1-34.5)	28.9 (24.8-32.9)
Salvador	26.4 (23.5-29.3)	23.9 (21.1-26.8)	25.7 (22.8-28.6)	26.0 (23.2-28.7)	26.2 (23.4-29.1)	28.2 (25.2-31.1)	30.3 (27.1-33.6)	28.1 (25.1-31.2)	28.4 (25.0-31.9)	31.8 (28.3-35.2)	29.7 (26.6-32.9)
São Luís	22.0 (19.2-24.7)	22.5 (19.7-25.2)	23.2 (20.4-25.9)	23.0 (20.2-25.9)	24.8 (22.1-27.6)	26.8 (23.8-29.8)	27.3 (24.1-30.5)	25.3 (22.3-28.3)	24.7 (21.4-28.0)	27.7 (24.7-30.8)	27.1 (23.9-30.4)
São Paulo	27.6 (24.9-30.3)	25.0 (22.3-27.8)	26.7 (24.0-29.4)	27.8 (24.8-30.9)	28.9 (25.9-32.0)	26.4 (23.5-29.4)	28.7 (25.6-31.7)	28.6 (25.6-31.6)	30.9 (27.4-34.5)	29.6 (26.5-32.8)	28.2 (25.0-31.3)
Teresina	21.8 (18.7-24.8)	22.5 (19.8-25.3)	25.3 (21.9-28.6)	24.7 (21.8-27.7)	23.9 (21.1-26.7)	26.9 (23.8-30.0)	26.5 (23.0-30.0)	29.8 (26.3-33.3)	29.0 (25.3-32.7)	30.1 (26.7-33.4)	28.4 (25.1-31.7)
Vitória	23.8 (21.2-26.4)	22.0 (19.4-24.6)	26.0 (23.2-28.9)	25.8 (22.9-28.6)	24.4 (21.8-27.1)	26.4 (23.4-29.4)	26.9 (23.6-30.1)	27.5 (24.3-30.7)	31.3 (27.5-35.1)	28.3 (24.6-32.0)	26.8 (23.3-30.2)
State capitals overall	25.7 (24.8-26.6)	25.0 (24.1-26.0)	26.2 (25.3-27.1)	26.9 (25.9-27.9)	28.2 (27.1-29.3)	27.4 (26.5-28.4)	28.8 (27.8-29.9)	28.8 (27.8-29.8)	29.9 (28.7-31.0)	29.7 (28.6-30.8)	29.4 (28.3-30.5)
