# Supplementary material for: Adult body weight trends in 27 urban populations of Brazil from 2006 to 2016: A population-based study
Source: PLoS One. 2019 Mar 6;14(3):e0213254. doi: 10.1371/journal.pone.0213254 (PMC6402686; doi:10.1371/journal.pone.0213254)
Supplement: S9 Table — Numbers in brackets show 95% confidence intervals. (PDF) [file pone.0213254.s009.pdf]

S9 Table. Age-standardized prevalence (%) of moderate obesity (30 kg/m² ≤ BMI < 35 kg/m²) in Brazil's state capitals, from 2006 to 2016, among men. Numbers in brackets show 95% confidence intervals.

State capital	2006	2007	2008	2009	2010	2011	2012	2013	2014	2015	2016
Aracaju	10.8 (7.8-13.8)	8.3 (5.9-10.6)	10.5 (7.9-13.1)	14.1 (10.8-17.5)	12.8 (9.4-16.1)	10.4 (7.8-13.1)	15.1 (11.5-18.6)	10.9 (8.4-13.5)	13.5 (10.0-17.0)	14.1 (11.0-17.2)	15.4 (11.9-19.0)
Belém	13.6 (10.6-16.7)	13.4 (10.7-16.2)	12.4 (9.5-15.3)	9.6 (7.1-12.1)	12.7 (9.7-15.7)	13.8 (11.0-16.6)	13.4 (10.0-16.7)	12.8 (9.9-15.7)	17.8 (13.3-22.3)	15.0 (11.8-18.2)	15.8 (12.2-19.4)
Belo Horizonte	8.0 (5.8-10.2)	8.9 (6.5-11.2)	8.7 (6.4-10.9)	8.0 (5.9-10.2)	9.2 (7.0-11.4)	10.4 (8.1-12.8)	10.8 (8.0-13.6)	10.3 (7.5-13.0)	10.7 (7.5-13.8)	12.7 (9.9-15.5)	11.7 (9.1-14.3)
Boa Vista	11.3 (8.4-14.2)	11.2 (8.4-14.0)	11.6 (7.1-16.2)	12.7 (9.4-15.9)	11.4 (8.5-14.4)	14.6 (11.2-18.0)	11.7 (8.7-14.7)	16.3 (12.7-19.9)	14.5 (11.1-18.0)	18.2 (13.6-22.9)	17.8 (13.4-22.2)
Campo Grande	9.5 (7.1-11.8)	13.5 (10.7-16.2)	10.8 (8.3-13.3)	13.5 (10.4-16.5)	12.4 (10.0-14.9)	15.0 (12.2-17.8)	15.8 (12.4-19.1)	11.5 (8.7-14.3)	13.5 (9.5-17.4)	15.3 (12.4-18.2)	16.8 (13.1-20.4)
Cuiabá	9.0 (6.8-11.3)	11.0 (8.7-13.4)	13.2 (10.6-15.9)	12.0 (9.5-14.6)	15.5 (12.4-18.7)	15.0 (12.3-17.8)	13.8 (10.5-17.0)	14.8 (11.8-17.9)	13.2 (9.9-16.5)	13.6 (10.0-17.1)	18.2 (14.8-21.6)
Curitiba	10.5 (8.1-12.9)	11.3 (8.7-13.8)	12.5 (9.9-15.0)	11.5 (9.0-14.1)	13.8 (11.2-16.5)	12.1 (9.5-14.7)	12.2 (9.4-15.0)	13.7 (10.5-17.0)	12.5 (9.1-15.8)	10.6 (8.4-12.9)	16.0 (12.1-20.0)
Federal District	10.5 (7.2-13.8)	8.9 (6.7-11.1)	7.6 (5.5-9.7)	7.0 (4.3-9.7)	9.2 (6.2-12.2)	12.0 (9.4-14.5)	10.7 (8.2-13.2)	12.4 (9.6-15.3)	11.6 (8.3-15.0)	11.3 (7.9-14.7)	13.1 (9.1-17.2)
Florianópolis	9.0 (6.7-11.2)	8.8 (6.6-11.0)	10.9 (8.4-13.3)	10.5 (8.2-12.8)	11.2 (8.7-13.6)	13.6 (10.9-16.4)	12.6 (9.6-15.5)	12.8 (10.1-15.5)	10.2 (7.3-13.1)	13.3 (9.8-16.8)	10.2 (7.1-13.2)
Fortaleza	11.6 (8.3-14.9)	11.6 (8.9-14.4)	13.7 (10.8-16.6)	12.8 (9.7-15.8)	14.0 (11.0-17.0)	16.8 (13.4-20.2)	14.7 (11.0-18.3)	16.2 (12.6-19.9)	15.7 (11.9-19.4)	17.2 (13.7-20.7)	15.5 (12.3-18.6)
Goiânia	7.7 (5.9-9.6)	9.3 (7.0-11.5)	8.7 (6.6-10.7)	9.5 (7.2-11.8)	10.3 (8.0-12.5)	10.4 (8.0-12.8)	9.1 (6.8-11.4)	15.3 (12.2-18.4)	12.0 (8.9-15.2)	11.2 (6.5-15.9)	12.0 (9.1-14.9)
João Pessoa	13.0 (10.0-16.0)	11.4 (8.6-14.3)	11.2 (8.5-13.9)	8.5 (6.3-10.8)	12.0 (8.8-15.2)	14.3 (11.2-17.3)	18.3 (14.0-22.5)	12.5 (9.7-15.4)	13.6 (9.7-17.4)	13.8 (10.5-17.0)	17.6 (13.5-21.8)
Macapá	14.1 (10.9-17.3)	16.0 (12.6-19.3)	11.7 (8.9-14.5)	14.1 (10.7-17.4)	14.4 (11.3-17.5)	14.9 (11.7-18.0)	11.7 (8.4-15.0)	17.1 (13.3-21.0)	12.1 (9.1-15.1)	15.8 (12.3-19.4)	16.5 (13.0-20.1)

Maceió	10.4 (7.6-13.2)	10.7 (7.7-13.7)	11.7 (8.5-14.8)	8.2 (5.8-10.6)	13.9 (10.5-17.4)	13.1 (10.0-16.3)	15.5 (11.4-19.5)	14.3 (11.2-17.5)	16.4 (11.6-21.2)	14.6 (11.5-17.8)	14.0 (10.5-17.5)
Manaus	10.2 (7.8-12.5)	11.6 (9.0-14.3)	12.0 (9.3-14.8)	13.0 (9.9-16.0)	13.8 (10.9-16.6)	15.8 (12.5-19.1)	15.0 (11.5-18.6)	14.5 (11.1-17.8)	13.2 (9.9-16.5)	18.3 (13.7-22.9)	14.8 (11.6-17.9)
Natal	10.3 (7.9-12.7)	13.3 (10.1-16.4)	10.8 (8.1-13.5)	12.0 (9.0-15.0)	11.2 (8.4-13.9)	14.6 (10.9-18.4)	14.5 (11.3-17.8)	14.3 (10.7-17.9)	12.2 (8.5-15.9)	13.9 (11.0-16.8)	15.8 (12.4-19.2)
Palmas	10.2 (7.3-13.2)	8.3 (6.0-10.6)	11.9 (8.6-15.3)	9.7 (7.4-12.0)	11.9 (9.0-14.8)	11.4 (8.5-14.2)	13.6 (10.1-17.1)	13.8 (10.1-17.5)	13.6 (10.2-17.0)	11.4 (8.6-14.1)	12.1 (9.5-14.7)
Porto Alegre	8.9 (6.7-11.2)	10.8 (8.2-13.4)	12.1 (9.5-14.7)	9.4 (7.2-11.7)	11.4 (8.7-14.0)	11.6 (9.1-14.1)	15.0 (11.5-18.5)	12.6 (9.5-15.7)	17.8 (13.6-21.9)	16.0 (12.5-19.5)	13.7 (10.5-16.9)
Porto Velho	10.3 (7.7-12.8)	11.6 (8.8-14.4)	11.8 (8.9-14.7)	14.7 (11.5-17.9)	11.5 (8.8-14.3)	13.8 (10.9-16.6)	14.7 (11.3-18.1)	15.1 (12.1-18.1)	15.3 (11.3-19.3)	13.1 (9.9-16.2)	17.8 (13.1-22.4)
Recife	8.8 (6.5-11.2)	10.1 (7.6-12.5)	11.3 (8.2-14.4)	9.8 (7.1-12.6)	14.8 (11.6-18.1)	10.6 (8.2-13.0)	14.1 (10.7-17.6)	12.8 (9.2-16.4)	11.4 (8.3-14.4)	12.5 (9.9-15.1)	15.9 (12.7-19.0)
Rio Branco	10.2 (7.7-12.7)	11.3 (8.2-14.3)	9.5 (6.6-12.4)	11.6 (8.6-14.6)	13.1 (10.1-16.0)	13.5 (10.2-16.9)	14.0 (10.2-17.9)	13.4 (9.9-16.9)	16.5 (12.0-20.9)	18.2 (13.9-22.6)	17.4 (14.1-20.8)
Rio de Janeiro	8.4 (6.2-10.5)	10.6 (8.2-12.9)	10.2 (7.5-12.8)	14.3 (11.2-17.4)	10.4 (7.9-13.0)	12.9 (10.1-15.7)	12.2 (9.1-15.2)	16.2 (13.0-19.5)	15.3 (11.6-18.9)	12.3 (8.5-16.0)	14.3 (10.6-18.0)
Salvador	8.3 (5.9-10.7)	8.2 (6.0-10.5)	8.3 (6.0-10.5)	8.6 (6.5-10.7)	7.3 (5.4-9.2)	9.6 (7.4-11.8)	8.1 (5.7-10.5)	10.8 (8.1-13.6)	12.3 (8.8-15.8)	12.2 (9.2-15.3)	14.1 (10.9-17.3)
São Luís	7.0 (4.8-9.2)	8.9 (6.3-11.5)	9.3 (6.0-12.5)	8.6 (6.4-10.7)	11.6 (8.8-14.3)	10.3 (7.8-12.9)	11.8 (8.6-14.9)	10.2 (7.2-13.2)	10.1 (7.1-13.1)	12.0 (8.2-15.8)	9.6 (7.1-12.0)
São Paulo	7.6 (5.5-9.7)	12.9 (10.2-15.6)	11.6 (8.9-14.2)	13.2 (10.2-16.2)	10.0 (7.7-12.3)	10.8 (8.5-13.2)	14.5 (11.5-17.6)	12.2 (9.7-14.6)	12.3 (9.3-15.4)	15.7 (12.7-18.8)	12.3 (9.7-14.9)
Teresina	8.9 (6.5-11.2)	10.1 (7.1-13.1)	10.0 (7.4-12.6)	11.1 (8.2-13.9)	12.3 (9.2-15.3)	9.9 (7.5-12.4)	14.0 (9.6-18.4)	12.2 (8.5-15.9)	12.5 (9.2-15.8)	13.7 (10.6-16.8)	12.2 (9.1-15.3)
Vitória	8.4 (6.1-10.6)	11.3 (8.8-13.7)	10.2 (7.9-12.5)	9.3 (7.1-11.5)	11.3 (8.8-13.9)	9.9 (7.6-12.3)	13.0 (10.0-16.0)	12.0 (9.2-14.8)	12.3 (9.5-15.2)	10.8 (7.8-13.9)	10.0 (7.3-12.6)
State capitals overall	9.1 (8.3-9.8)	11.1 (10.3-12.0)	10.8 (9.9-11.7)	11.5 (10.6-12.5)	11.1 (10.2-11.9)	12.1 (11.3-12.9)	13.0 (12.0-14.0)	13.3 (12.4-14.2)	13.2 (12.2-14.3)	14.0 (13.0-15.1)	13.8 (12.8-14.7)
