# Supplementary material for: Adult body weight trends in 27 urban populations of Brazil from 2006 to 2016: A population-based study
Source: PLoS One. 2019 Mar 6;14(3):e0213254. doi: 10.1371/journal.pone.0213254 (PMC6402686; doi:10.1371/journal.pone.0213254)
Supplement: S10 Table — Numbers in brackets show 95% confidence intervals. (PDF) [file pone.0213254.s010.pdf]

S10 Table. Age-standardized prevalence (%) of moderate obesity (30 kg/m² ≤ BMI < 35 kg/m²) in Brazil's state capitals, from 2006 to 2016, among women. Numbers in brackets show 95% confidence intervals.

State capital	2006	2007	2008	2009	2010	2011	2012	2013	2014	2015	2016
Aracaju	9.6 (7.6-11.5)	7.6 (5.8-9.4)	10.8 (8.6-13.0)	9.7 (7.7-11.6)	10.1 (8.2-12.0)	12.3 (10.1-14.4)	10.9 (8.6-13.3)	11.9 (9.5-14.2)	11.5 (9.2-13.8)	10.8 (8.7-12.8)	13.0 (10.6-15.4)
Belém	7.3 (5.7-9.0)	9.5 (7.3-11.7)	9.7 (7.4-12.0)	10.1 (7.9-12.3)	10.4 (8.3-12.6)	9.2 (7.3-11.2)	8.9 (6.8-11.1)	10.5 (8.3-12.7)	13.1 (10.2-16.0)	12.1 (9.8-14.4)	11.7 (9.4-14.0)
Belo Horizonte	6.8 (5.1-8.4)	8.7 (6.8-10.6)	9.0 (7.1-10.9)	10.2 (8.2-12.1)	9.1 (7.3-11.0)	9.9 (7.9-11.9)	11.3 (9.1-13.5)	9.9 (7.9-11.9)	11.8 (9.3-14.4)	11.4 (9.3-13.5)	11.0 (8.9-13.2)
Boa Vista	8.3 (6.4-10.2)	8.9 (6.7-11.1)	10.3 (8.1-12.6)	10.4 (8.3-12.4)	12.8 (10.2-15.3)	13.1 (10.4-15.8)	11.7 (9.3-14.2)	12.0 (9.5-14.5)	13.0 (10.1-15.8)	15.8 (12.7-18.9)	11.4 (9.0-13.8)
Campo Grande	9.2 (7.2-11.2)	9.8 (7.8-11.7)	10.8 (8.8-12.7)	11.7 (9.5-13.8)	12.7 (10.6-14.8)	11.3 (9.3-13.3)	14.6 (11.9-17.3)	15.0 (12.2-17.8)	15.0 (11.9-18.0)	15.9 (13.3-18.5)	12.2 (9.4-14.9)
Cuiabá	9.2 (7.1-11.3)	9.8 (7.7-11.9)	10.4 (8.2-12.5)	11.3 (9.4-13.3)	11.4 (9.4-13.4)	11.5 (9.3-13.6)	12.8 (10.4-15.3)	15.9 (13.2-18.5)	15.5 (12.7-18.3)	13.0 (9.5-16.4)	14.5 (12.1-17.0)
Curitiba	8.3 (6.7-9.9)	9.5 (7.7-11.3)	8.7 (7.1-10.4)	8.8 (6.9-10.7)	11.8 (9.8-13.7)	10.9 (9.1-12.8)	10.6 (8.5-12.7)	12.6 (10.1-15.1)	14.3 (11.0-17.7)	12.0 (9.7-14.3)	10.3 (7.6-13.0)
Federal District	7.2 (5.3-9.2)	7.6 (6.0-9.2)	10.7 (8.7-12.7)	7.0 (4.6-9.5)	6.6 (4.0-9.2)	10.1 (8.2-11.9)	11.7 (9.5-13.9)	9.9 (7.9-11.8)	8.8 (6.8-10.9)	11.8 (8.8-14.8)	12.0 (8.4-15.6)
Florianópolis	8.5 (6.7-10.4)	7.5 (5.8-9.2)	8.2 (6.5-9.9)	10.0 (8.0-12.0)	9.3 (7.6-11.1)	10.3 (8.2-12.3)	10.6 (8.3-13.0)	8.8 (6.9-10.7)	8.3 (6.2-10.4)	9.3 (7.0-11.5)	9.2 (6.8-11.5)
Fortaleza	6.6 (4.9-8.3)	9.3 (7.1-11.5)	10.6 (8.2-12.9)	11.5 (9.2-13.9)	11.9 (9.5-14.3)	12.1 (9.8-14.3)	13.5 (10.7-16.3)	10.3 (8.2-12.4)	11.1 (8.7-13.5)	13.4 (11.0-15.7)	12.5 (9.9-15.2)
Goiânia	7.6 (5.9-9.2)	8.7 (6.9-10.5)	8.9 (7.1-10.6)	8.8 (7.0-10.6)	9.0 (7.2-10.8)	10.7 (8.7-12.6)	11.3 (9.2-13.4)	11.4 (9.3-13.5)	12.5 (9.8-15.2)	9.6 (6.3-12.8)	10.9 (8.6-13.3)
João Pessoa	8.7 (6.8-10.5)	9.3 (7.2-11.4)	10.4 (8.1-12.8)	12.1 (9.5-14.6)	10.6 (8.3-12.9)	12.4 (10.0-14.8)	13.3 (10.8-15.8)	13.0 (10.5-15.5)	12.3 (9.7-14.9)	16.3 (13.3-19.2)	14.5 (10.9-18.1)
Macapá	9.7 (7.4-11.9)	10.7 (8.5-13.0)	10.8 (8.3-13.3)	10.1 (7.7-12.6)	8.9 (6.6-11.2)	11.2 (8.9-13.6)	13.7 (10.5-16.8)	10.4 (8.3-12.5)	13.7 (10.9-16.4)	11.1 (8.9-13.4)	11.5 (9.3-13.8)

Maceió	10.1 (7.7-12.5)	8.8 (6.9-10.6)	7.4 (5.7-9.1)	8.6 (6.6-10.7)	9.8 (7.7-12.0)	13.0 (10.3-15.6)	15.0 (11.7-18.2)	10.9 (8.6-13.2)	13.8 (11.1-16.4)	14.0 (11.5-16.5)	13.4 (10.8-16.0)
Manaus	10.8 (8.6-13.0)	11.5 (9.2-13.7)	11.7 (9.4-14.1)	11.3 (9.1-13.4)	12.8 (10.5-15.0)	13.1 (10.8-15.3)	14.5 (11.9-17.1)	12.8 (10.4-15.2)	14.8 (11.4-18.3)	15.0 (11.8-18.2)	15.0 (12.1-18.0)
Natal	7.5 (5.8-9.2)	8.3 (6.5-10.2)	9.9 (7.8-12.1)	10.4 (8.3-12.5)	13.0 (10.6-15.5)	10.1 (8.1-12.2)	12.3 (10.0-14.6)	10.8 (8.7-12.9)	12.9 (10.3-15.5)	12.1 (9.7-14.5)	13.4 (10.7-16.1)
Palmas	8.9 (6.2-11.6)	8.6 (6.4-10.8)	7.7 (5.5-9.9)	7.2 (5.1-9.2)	9.0 (6.8-11.2)	11.6 (8.7-14.6)	11.9 (9.2-14.6)	11.1 (8.1-14.1)	10.7 (8.1-13.3)	11.5 (8.9-14.1)	11.1 (8.8-13.4)
Porto Alegre	8.0 (6.3-9.7)	9.1 (7.1-11.0)	10.2 (8.1-12.2)	11.7 (9.3-14.1)	9.8 (7.7-11.8)	13.0 (10.7-15.4)	11.9 (9.4-14.4)	10.5 (8.1-12.8)	10.1 (7.2-13.0)	13.7 (10.6-16.8)	10.2 (7.9-12.5)
Porto Velho	10.7 (8.4-13.0)	11.8 (9.4-14.3)	12.3 (10.0-14.7)	12.3 (9.9-14.8)	11.2 (8.9-13.5)	10.7 (8.7-12.7)	13.8 (11.1-16.5)	12.1 (9.2-15.0)	13.2 (10.1-16.2)	16.9 (13.4-20.5)	13.6 (10.8-16.5)
Recife	9.3 (7.4-11.2)	8.4 (6.2-10.5)	8.3 (6.6-9.9)	9.5 (7.4-11.6)	10.6 (8.5-12.6)	11.0 (9.0-13.1)	11.5 (9.1-14.0)	12.5 (10.2-14.7)	13.7 (10.9-16.6)	14.3 (12.0-16.7)	13.6 (10.9-16.3)
Rio Branco	10.8 (8.6-13.0)	8.8 (6.8-10.9)	11.3 (8.8-13.9)	9.8 (7.7-11.9)	11.8 (9.6-14.0)	12.9 (10.6-15.1)	16.5 (13.4-19.5)	12.6 (10.0-15.2)	12.4 (9.5-15.2)	14.6 (11.8-17.4)	16.5 (13.9-19.1)
Rio de Janeiro	9.2 (7.4-10.9)	9.4 (7.5-11.3)	9.9 (7.9-11.9)	10.4 (8.3-12.5)	11.7 (9.5-13.9)	11.3 (9.2-13.4)	13.5 (10.9-16.2)	11.0 (8.8-13.1)	10.9 (8.5-13.3)	13.2 (10.1-16.4)	14.0 (10.9-17.0)
Salvador	10.3 (8.2-12.5)	10.8 (8.8-12.9)	10.3 (8.4-12.3)	11.3 (9.2-13.5)	10.8 (8.9-12.7)	10.8 (8.7-12.8)	12.3 (9.9-14.8)	10.0 (8.1-11.9)	12.8 (10.4-15.3)	12.2 (9.9-14.5)	14.0 (11.3-16.7)
São Luís	7.2 (5.3-9.1)	7.0 (5.0-8.9)	8.4 (6.5-10.3)	8.9 (6.7-11.1)	9.3 (7.3-11.3)	10.3 (8.0-12.6)	7.8 (5.9-9.8)	9.9 (7.7-12.1)	11.8 (9.0-14.6)	10.7 (8.6-12.7)	14.6 (11.4-17.8)
São Paulo	7.8 (6.1-9.5)	8.9 (7.1-10.8)	9.6 (7.7-11.4)	8.8 (7.1-10.6)	10.9 (9.0-12.7)	11.3 (9.4-13.1)	11.7 (9.6-13.9)	12.6 (10.5-14.6)	12.0 (9.7-14.3)	13.8 (11.3-16.3)	13.0 (10.8-15.2)
Teresina	7.7 (5.9-9.5)	7.6 (5.8-9.4)	7.9 (6.0-9.7)	10.0 (7.7-12.4)	8.4 (6.4-10.5)	10.6 (8.0-13.1)	10.7 (8.2-13.2)	9.4 (7.1-11.6)	9.0 (6.6-11.5)	9.3 (7.2-11.5)	12.7 (9.8-15.6)
Vitória	6.5 (5.0-7.9)	8.4 (6.7-10.1)	8.1 (6.3-9.9)	9.0 (7.1-10.9)	9.7 (7.5-11.9)	10.4 (8.4-12.4)	9.5 (7.4-11.6)	10.9 (8.6-13.2)	11.2 (8.8-13.5)	10.2 (7.9-12.5)	12.8 (10.2-15.4)
State capitals overall	8.3 (7.8-8.9)	9.1 (8.5-9.7)	9.7 (9.1-10.4)	9.8 (9.2-10.4)	10.6 (9.9-11.2)	11.2 (10.6-11.8)	12.0 (11.3-12.8)	11.5 (10.8-12.2)	11.9 (11.1-12.7)	12.8 (12.0-13.6)	12.8 (12.1-13.6)
