# Supplementary material for: Adult body weight trends in 27 urban populations of Brazil from 2006 to 2016: A population-based study
Source: PLoS One. 2019 Mar 6;14(3):e0213254. doi: 10.1371/journal.pone.0213254 (PMC6402686; doi:10.1371/journal.pone.0213254)
Supplement: S11 Table — Numbers in brackets show 95% confidence intervals. (PDF) [file pone.0213254.s011.pdf]

S11 Table. Age-standardized prevalence (%) of severe obesity (35 kg/m² ≤ BMI < 40 kg/m²) in Brazil's state capitals, from 2006 to 2016, among men. Numbers in brackets show 95% confidence intervals.

State capital	2006	2007	2008	2009	2010	2011	2012	2013	2014	2015	2016
Aracaju	3.2 (1.3-5.2)	1.9 (0.3-3.6)	2.4 (0.9-3.8)	3.8 (1.4-6.1)	1.7 (0.6-2.8)	3.3 (1.3-5.2)	2.6 (1.1-4.1)	3.9 (1.7-6.1)	4.1 (1.6-6.6)	2.3 (1.0-3.6)	3.0 (1.6-4.4)
Belém	2.1 (0.9-3.3)	2.2 (1.0-3.4)	1.7 (0.5-2.9)	2.2 (0.9-3.5)	3.5 (1.9-5.0)	1.7 (0.8-2.6)	3.3 (1.4-5.2)	2.9 (1.6-4.1)	3.2 (1.4-5.1)	3.5 (1.7-5.3)	4.8 (2.4-7.1)
Belo Horizonte	1.3 (0.5-2.2)	1.0 (0.4-1.5)	1.9 (0.9-2.9)	1.1 (0.4-1.8)	2.3 (1.1-3.6)	1.8 (0.6-3.1)	1.9 (0.8-3.0)	2.2 (1.1-3.4)	3.1 (1.4-4.9)	2.1 (0.9-3.2)	2.5 (1.1-3.8)
Boa Vista	2.4 (1.0-3.9)	1.2 (0.4-2.1)	3.1 (1.3-4.9)	2.4 (1.0-3.9)	2.2 (1.1-3.4)	1.7 (0.5-2.8)	3.2 (1.6-4.7)	2.9 (1.6-4.3)	3.9 (1.7-6.1)	5.8 (2.3-9.2)	3.9 (0.2-7.5)
Campo Grande	2.9 (1.6-4.3)	2.9 (1.5-4.3)	2.9 (1.6-4.3)	2.0 (0.9-3.1)	2.1 (1.0-3.3)	3.3 (1.8-4.9)	3.0 (1.6-4.5)	2.7 (1.4-4.0)	3.9 (1.7-6.1)	2.8 (1.7-3.9)	2.7 (1.3-4.2)
Cuiabá	4.0 (2.2-5.8)	3.6 (2.0-5.1)	1.5 (0.6-2.4)	1.5 (0.7-2.4)	1.9 (0.9-2.9)	3.0 (1.6-4.5)	5.0 (2.8-7.3)	4.8 (1.7-8.0)	4.0 (1.5-6.4)	2.8 (1.4-4.2)	5.3 (3.1-7.4)
Curitiba	1.6 (0.7-2.6)	1.9 (0.9-2.8)	1.4 (0.6-2.2)	1.8 (0.9-2.8)	2.0 (1.0-2.9)	2.8 (1.6-4.0)	2.5 (1.3-3.7)	3.6 (1.4-5.8)	3.4 (1.6-5.2)	2.5 (1.4-3.5)	3.5 (1.9-5.2)
Federal District	0.9 (-0.0-1.8)	1.4 (0.6-2.3)	2.3 (0.9-3.7)	1.4 (0.5-2.4)	1.7 (0.2-3.3)	1.8 (0.7-2.9)	2.5 (1.2-3.7)	2.8 (1.2-4.5)	2.6 (1.2-4.1)	2.3 (0.8-3.9)	3.2 (0.3-6.0)
Florianópolis	1.4 (0.3-2.4)	1.3 (0.4-2.1)	0.9 (0.2-1.6)	2.8 (1.1-4.4)	3.4 (1.7-5.2)	2.9 (1.4-4.4)	2.1 (0.9-3.3)	3.1 (1.3-4.8)	1.4 (0.3-2.6)	1.9 (0.6-3.2)	2.6 (0.9-4.3)
Fortaleza	1.6 (0.7-2.6)	2.6 (1.4-3.9)	2.0 (0.7-3.2)	2.2 (1.0-3.4)	4.8 (2.4-7.2)	3.4 (1.5-5.2)	3.7 (1.8-5.6)	2.2 (1.2-3.3)	3.8 (0.7-7.0)	3.2 (1.4-5.1)	3.7 (2.0-5.4)
Goiânia	1.7 (0.7-2.6)	2.6 (1.2-4.0)	1.3 (0.5-2.2)	0.9 (0.2-1.7)	2.1 (1.1-3.1)	2.3 (1.1-3.5)	2.0 (0.9-3.1)	2.3 (1.1-3.5)	1.5 (0.3-2.7)	3.1 (1.0-5.2)	4.2 (2.2-6.2)
João Pessoa	2.5 (0.9-4.0)	2.5 (1.3-3.7)	3.7 (1.7-5.7)	1.5 (0.7-2.2)	3.0 (1.3-4.7)	1.9 (0.8-3.0)	2.1 (0.3-3.9)	2.0 (0.9-3.2)	2.1 (0.8-3.4)	3.0 (1.4-4.6)	3.5 (1.2-5.7)
Macapá	2.9 (1.4-4.3)	3.3 (1.7-4.9)	2.6 (0.8-4.4)	2.0 (0.5-3.5)	2.7 (1.1-4.3)	5.1 (2.9-7.4)	3.2 (1.3-5.1)	4.8 (2.3-7.2)	3.1 (1.3-4.8)	4.3 (1.9-6.8)	1.6 (0.7-2.4)

Maceió	2.0 (0.9-3.1)	1.6 (0.6-2.5)	2.7 (1.3-4.2)	2.3 (0.6-3.9)	1.3 (0.5-2.0)	3.2 (1.4-5.0)	2.7 (1.1-4.2)	3.7 (1.5-5.9)	3.1 (1.2-5.0)	1.8 (0.9-2.8)	3.5 (1.7-5.3)
Manaus	3.3 (1.6-5.0)	1.9 (0.8-3.0)	2.3 (1.1-3.6)	2.6 (1.2-4.0)	3.3 (1.9-4.6)	4.0 (2.1-5.8)	3.5 (0.8-6.2)	3.1 (1.2-5.1)	3.7 (1.5-5.8)	9.4 (4.9-13.9)	3.1 (1.8-4.4)
Natal	4.6 (2.5-6.6)	1.8 (0.6-3.0)	2.1 (0.5-3.7)	2.4 (0.9-3.9)	2.8 (1.0-4.5)	2.7 (1.5-4.0)	3.9 (2.1-5.6)	3.3 (1.2-5.4)	2.0 (0.6-3.5)	4.3 (1.4-7.1)	2.3 (1.1-3.4)
Palmas	1.1 (0.2-2.0)	1.5 (0.3-2.7)	1.7 (0.8-2.7)	0.9 (0.3-1.5)	3.6 (1.4-5.9)	2.9 (1.6-4.2)	1.2 (0.3-2.2)	6.9 (0.8-12.9)	3.5 (1.7-5.2)	2.8 (1.2-4.4)	2.4 (1.2-3.6)
Porto Alegre	2.1 (0.9-3.3)	2.3 (1.1-3.4)	2.3 (0.9-3.7)	2.1 (1.0-3.3)	2.8 (1.3-4.4)	4.1 (2.2-6.0)	2.4 (1.1-3.7)	3.4 (1.7-5.1)	4.9 (2.4-7.4)	5.6 (3.2-8.0)	3.9 (2.0-5.9)
Porto Velho	2.6 (1.2-3.9)	2.8 (1.2-4.4)	1.7 (0.7-2.7)	4.3 (2.2-6.4)	2.7 (1.3-4.1)	3.2 (1.9-4.5)	3.0 (1.4-4.7)	2.2 (1.2-3.2)	3.3 (1.6-4.9)	3.8 (1.8-5.8)	2.6 (1.3-4.0)
Recife	2.5 (1.1-3.9)	2.3 (1.2-3.4)	2.8 (1.0-4.6)	3.6 (1.9-5.2)	3.6 (2.0-5.3)	3.0 (1.7-4.2)	2.5 (1.0-4.0)	2.2 (1.1-3.4)	3.7 (1.9-5.4)	1.9 (0.8-3.1)	3.0 (1.5-4.5)
Rio Branco	3.6 (1.8-5.3)	2.3 (0.9-3.7)	3.2 (1.5-4.9)	3.4 (1.4-5.4)	2.6 (1.2-4.1)	3.1 (1.6-4.7)	3.1 (1.5-4.7)	2.9 (1.2-4.6)	4.1 (1.8-6.5)	2.6 (1.2-4.0)	5.2 (3.3-7.1)
Rio de Janeiro	2.1 (1.0-3.3)	2.8 (1.3-4.2)	2.3 (0.9-3.6)	2.5 (1.2-3.8)	2.5 (1.4-3.7)	3.0 (1.6-4.4)	3.7 (1.8-5.7)	4.4 (2.6-6.2)	3.6 (1.7-5.6)	1.9 (0.4-3.3)	4.4 (2.4-6.3)
Salvador	1.1 (0.3-1.9)	1.4 (0.5-2.2)	1.8 (0.8-2.8)	1.5 (0.4-2.7)	1.6 (0.8-2.5)	2.3 (1.1-3.4)	1.3 (0.3-2.3)	1.7 (0.6-2.7)	3.2 (1.2-5.2)	1.5 (0.4-2.5)	2.3 (0.9-3.7)
São Luís	2.0 (0.6-3.5)	1.9 (0.7-3.1)	1.5 (0.5-2.4)	0.9 (0.4-1.4)	1.9 (0.7-3.2)	1.6 (0.5-2.7)	2.4 (0.7-4.0)	1.9 (0.8-3.0)	2.6 (1.2-4.1)	2.3 (1.0-3.7)	2.8 (1.5-4.1)
São Paulo	0.9 (0.3-1.5)	1.5 (0.7-2.4)	3.2 (1.7-4.6)	1.5 (0.6-2.4)	3.1 (1.7-4.6)	2.4 (1.1-3.6)	1.4 (0.6-2.3)	3.1 (1.8-4.4)	3.2 (1.8-4.7)	3.5 (2.0-4.9)	2.0 (1.0-3.0)
Teresina	2.1 (0.8-3.3)	1.3 (0.5-2.1)	2.0 (0.7-3.3)	1.3 (0.4-2.2)	2.0 (0.8-3.1)	2.3 (1.1-3.4)	2.9 (1.3-4.6)	4.8 (2.0-7.6)	3.2 (1.0-5.3)	2.3 (0.9-3.7)	4.5 (2.2-6.8)
Vitória	2.5 (1.2-3.7)	1.9 (0.9-3.0)	1.7 (0.7-2.7)	1.1 (0.5-1.8)	2.7 (1.4-4.0)	1.8 (0.7-2.9)	3.8 (1.9-5.7)	3.3 (1.7-5.0)	2.2 (0.8-3.7)	1.0 (0.3-1.7)	2.1 (0.8-3.3)
State capitals overall	1.7 (1.4-2.0)	2.0 (1.6-2.3)	2.3 (1.9-2.8)	1.9 (1.5-2.2)	2.7 (2.3-3.2)	2.6 (2.2-3.1)	2.5 (2.1-2.9)	3.1 (2.7-3.6)	3.3 (2.7-3.9)	3.1 (2.6-3.6)	3.1 (2.7-3.6)
