# Supplementary material for: Adult body weight trends in 27 urban populations of Brazil from 2006 to 2016: A population-based study
Source: PLoS One. 2019 Mar 6;14(3):e0213254. doi: 10.1371/journal.pone.0213254 (PMC6402686; doi:10.1371/journal.pone.0213254)
Supplement: S12 Table — Numbers in brackets show 95% confidence intervals. (PDF) [file pone.0213254.s012.pdf]

S12 Table. Age-standardized prevalence (%) of severe obesity (35 kg/m² ≤ BMI < 40 kg/m²) in Brazil's state capitals, from 2006 to 2016, among women. Numbers in brackets show 95% confidence intervals.

State capital	2006	2007	2008	2009	2010	2011	2012	2013	2014	2015	2016
Aracaju	1.6 (0.9-2.3)	3.3 (1.9-4.7)	2.7 (1.6-3.7)	3.2 (1.9-4.5)	3.2 (1.9-4.4)	3.1 (2.0-4.3)	3.2 (1.9-4.5)	4.3 (2.9-5.7)	3.9 (2.3-5.6)	4.6 (3.1-6.0)	4.0 (2.8-5.2)
Belém	1.9 (1.0-2.7)	2.7 (1.4-3.9)	2.4 (1.3-3.5)	2.7 (1.5-3.9)	3.2 (2.0-4.4)	2.6 (1.6-3.5)	3.4 (2.1-4.8)	3.2 (2.0-4.4)	4.5 (2.6-6.4)	3.4 (2.0-4.9)	3.1 (2.0-4.2)
Belo Horizonte	1.6 (0.8-2.4)	2.4 (1.4-3.4)	2.1 (1.2-3.0)	2.0 (1.2-2.9)	2.4 (1.4-3.4)	3.4 (2.1-4.6)	2.0 (1.2-2.8)	2.6 (1.5-3.6)	4.0 (2.4-5.7)	4.5 (3.0-5.9)	3.3 (2.1-4.4)
Boa Vista	3.0 (1.7-4.4)	2.5 (1.5-3.6)	2.9 (1.7-4.1)	2.8 (1.5-4.0)	2.5 (1.5-3.5)	3.6 (2.2-5.1)	3.7 (2.3-5.1)	4.0 (2.6-5.4)	3.2 (1.9-4.5)	4.6 (2.7-6.4)	4.6 (2.3-6.8)
Campo Grande	3.1 (2.0-4.3)	3.2 (2.0-4.3)	2.3 (1.4-3.2)	3.2 (2.0-4.3)	3.7 (2.6-4.9)	3.8 (2.6-5.0)	5.3 (3.6-7.1)	3.6 (2.1-5.1)	6.3 (4.1-8.5)	4.8 (3.2-6.4)	3.9 (2.4-5.5)
Cuiabá	1.8 (1.0-2.6)	2.3 (1.4-3.3)	2.8 (1.6-3.9)	3.1 (1.8-4.3)	4.8 (3.3-6.4)	3.9 (2.6-5.2)	3.4 (2.1-4.7)	3.8 (2.5-5.1)	4.2 (2.3-6.1)	3.4 (2.0-4.8)	3.4 (2.2-4.6)
Curitiba	2.9 (1.8-4.1)	2.3 (1.4-3.2)	3.0 (1.9-4.0)	2.2 (1.4-3.0)	4.1 (2.9-5.2)	3.3 (2.1-4.5)	3.5 (2.2-4.7)	2.5 (1.5-3.5)	3.8 (2.0-5.7)	3.8 (2.5-5.1)	4.4 (2.3-6.5)
Federal District	3.1 (1.8-4.4)	2.3 (1.3-3.3)	2.3 (1.2-3.5)	2.2 (0.7-3.6)	2.6 (0.8-4.5)	3.2 (1.9-4.5)	1.5 (0.7-2.3)	3.0 (2.0-4.0)	3.5 (2.1-4.9)	1.3 (0.4-2.2)	3.6 (1.9-5.2)
Florianópolis	1.0 (0.4-1.7)	2.8 (1.6-4.0)	2.3 (1.2-3.3)	2.6 (1.3-3.8)	3.2 (1.9-4.4)	2.1 (1.1-3.1)	2.6 (1.1-4.0)	3.5 (2.2-4.8)	4.2 (2.2-6.1)	3.4 (2.0-4.9)	4.3 (2.3-6.3)
Fortaleza	2.5 (1.3-3.7)	2.7 (1.6-3.8)	2.2 (1.1-3.4)	2.8 (1.5-4.0)	4.6 (2.8-6.3)	3.2 (2.0-4.4)	4.3 (2.5-6.2)	4.5 (3.0-6.0)	2.1 (1.2-3.0)	3.2 (2.0-4.5)	4.3 (2.5-6.1)
Goiânia	1.9 (1.1-2.7)	1.7 (0.9-2.4)	3.1 (2.1-4.2)	2.5 (1.5-3.4)	2.7 (1.7-3.8)	2.9 (2.0-3.9)	3.5 (2.2-4.8)	2.0 (1.1-2.9)	1.9 (0.9-2.9)	1.3 (0.5-2.0)	1.9 (1.1-2.8)
João Pessoa	3.0 (1.9-4.1)	2.7 (1.6-3.8)	2.8 (1.5-4.1)	2.3 (1.2-3.5)	2.2 (1.3-3.0)	2.2 (1.3-3.2)	3.7 (2.1-5.4)	3.2 (1.9-4.5)	3.0 (1.7-4.2)	3.7 (2.2-5.2)	3.6 (2.1-5.1)
Macapá	2.3 (1.3-3.2)	2.7 (1.4-4.0)	3.7 (2.3-5.1)	3.3 (2.0-4.6)	4.8 (2.9-6.6)	4.6 (2.8-6.4)	5.3 (3.2-7.5)	3.5 (2.2-4.8)	5.4 (3.3-7.5)	5.7 (3.7-7.7)	3.5 (2.3-4.7)

Maceió	3.1 (1.9-4.4)	2.3 (1.3-3.3)	3.6 (2.0-5.2)	3.5 (2.0-5.0)	3.4 (2.1-4.6)	2.7 (1.7-3.8)	4.3 (2.4-6.2)	3.8 (2.4-5.2)	3.9 (2.4-5.5)	4.2 (2.9-5.6)	5.1 (3.4-6.9)
Manaus	2.8 (1.6-4.0)	3.1 (1.9-4.3)	3.3 (2.1-4.5)	3.0 (1.9-4.1)	4.2 (2.8-5.6)	4.1 (2.8-5.5)	2.9 (1.6-4.2)	5.1 (3.4-6.7)	4.0 (2.3-5.8)	5.9 (3.7-8.2)	4.6 (2.9-6.3)
Natal	1.9 (1.1-2.7)	2.6 (1.6-3.7)	2.5 (1.1-3.9)	1.8 (1.0-2.5)	2.6 (1.4-3.8)	3.6 (2.3-4.9)	7.3 (4.8-9.8)	2.2 (1.2-3.2)	4.8 (2.6-7.1)	3.8 (2.1-5.5)	4.2 (2.0-6.5)
Palmas	2.0 (0.9-3.1)	2.5 (1.1-3.8)	1.8 (0.9-2.7)	1.8 (0.5-3.1)	2.4 (0.9-4.0)	1.8 (0.8-2.7)	3.1 (1.8-4.4)	2.1 (0.8-3.3)	4.0 (2.0-6.0)	2.5 (1.3-3.7)	2.7 (1.5-3.9)
Porto Alegre	2.9 (1.6-4.1)	2.4 (1.2-3.7)	1.9 (1.1-2.7)	2.5 (1.4-3.6)	2.9 (1.8-4.1)	4.5 (3.0-6.0)	5.2 (3.0-7.5)	3.7 (2.0-5.3)	6.4 (3.8-9.0)	3.1 (1.6-4.6)	5.6 (3.3-8.0)
Porto Velho	3.0 (1.9-4.2)	3.4 (2.1-4.8)	2.4 (1.3-3.5)	3.8 (2.5-5.1)	4.1 (2.5-5.8)	3.1 (1.8-4.5)	3.7 (2.3-5.1)	3.3 (1.9-4.7)	4.7 (3.0-6.4)	3.3 (2.0-4.7)	5.7 (3.5-7.9)
Recife	3.7 (2.2-5.3)	2.6 (1.5-3.6)	2.7 (1.5-3.8)	2.9 (1.8-4.0)	3.8 (2.4-5.3)	3.7 (2.4-4.9)	3.1 (1.7-4.5)	3.6 (2.4-4.9)	4.3 (2.8-5.9)	4.3 (2.7-5.9)	3.4 (2.2-4.6)
Rio Branco	2.2 (1.2-3.3)	3.5 (2.0-5.0)	3.9 (2.3-5.4)	4.3 (2.4-6.2)	4.9 (3.2-6.6)	4.2 (2.7-5.6)	6.1 (3.5-8.6)	6.2 (3.8-8.5)	4.4 (2.2-6.6)	4.1 (2.6-5.6)	4.4 (3.0-5.8)
Rio de Janeiro	2.8 (1.8-3.7)	2.8 (1.6-4.0)	2.2 (1.3-3.1)	3.1 (1.9-4.4)	3.7 (2.3-5.0)	3.6 (2.3-4.9)	4.2 (2.7-5.7)	4.4 (2.9-5.8)	4.1 (2.5-5.7)	5.1 (3.2-7.1)	3.4 (1.9-4.9)
Salvador	1.7 (0.9-2.5)	2.6 (1.4-3.7)	2.6 (1.5-3.6)	3.5 (2.4-4.7)	2.0 (1.1-2.9)	3.9 (2.6-5.2)	3.3 (2.1-4.6)	3.3 (2.1-4.5)	3.7 (2.3-5.2)	3.6 (2.2-5.0)	4.4 (3.0-5.9)
São Luís	2.2 (0.8-3.6)	2.0 (0.9-3.0)	2.4 (1.4-3.4)	2.6 (1.6-3.7)	2.3 (1.3-3.4)	2.3 (1.3-3.2)	3.3 (1.8-4.7)	2.6 (1.5-3.7)	4.0 (2.3-5.7)	3.2 (2.1-4.3)	2.9 (1.5-4.3)
São Paulo	2.6 (1.5-3.7)	2.2 (1.3-3.1)	2.8 (1.8-3.8)	3.5 (2.3-4.6)	2.8 (1.8-3.7)	3.5 (2.2-4.8)	3.3 (1.8-4.8)	3.1 (2.0-4.1)	2.8 (1.5-4.1)	3.9 (2.7-5.1)	4.5 (3.1-5.9)
Teresina	1.7 (0.9-2.6)	4.0 (2.2-5.7)	2.8 (1.4-4.2)	2.1 (1.1-3.0)	2.1 (1.1-3.0)	2.7 (1.5-3.9)	2.7 (1.6-3.9)	3.7 (2.1-5.2)	3.0 (1.5-4.5)	4.0 (2.4-5.5)	2.4 (1.3-3.5)
Vitória	1.6 (0.9-2.4)	1.5 (0.8-2.3)	2.3 (1.4-3.2)	2.7 (1.5-3.8)	3.0 (1.9-4.1)	3.6 (2.2-4.9)	2.7 (1.6-3.9)	3.6 (2.1-5.0)	2.6 (1.5-3.7)	4.5 (2.7-6.2)	2.7 (1.7-3.8)
State capitals overall	2.5 (2.1-2.8)	2.5 (2.2-2.8)	2.6 (2.3-2.9)	2.9 (2.6-3.3)	3.2 (2.8-3.5)	3.4 (3.0-3.8)	3.5 (3.1-4.0)	3.5 (3.1-3.8)	3.6 (3.2-4.1)	3.9 (3.5-4.4)	4.0 (3.5-4.4)
