# Supplementary material for: Adult body weight trends in 27 urban populations of Brazil from 2006 to 2016: A population-based study
Source: PLoS One. 2019 Mar 6;14(3):e0213254. doi: 10.1371/journal.pone.0213254 (PMC6402686; doi:10.1371/journal.pone.0213254)
Supplement: S13 Table — Numbers in brackets show 95% confidence intervals. (PDF) [file pone.0213254.s013.pdf]

S13 Table. Age-standardized prevalence (%) of morbid obesity (BMI ≥ 40 kg/m²) in Brazil's state capitals, from 2006 to 2016, among men. Numbers in brackets show 95% confidence intervals.

State capital	2006	2007	2008	2009	2010	2011	2012	2013	2014	2015	2016
Aracaju	1.2 (0.0-2.4)	0.6 (-0.2-1.3)	0.5 (-0.2-1.1)	1.0 (0.1-2.0)	2.2 (0.6-3.8)	0.9 (-0.1-1.9)	2.0 (0.4-3.6)	1.0 (0.2-1.7)	1.1 (0.0-2.1)	1.2 (0.1-2.3)	2.2 (0.3-4.2)
Belém	1.3 (0.2-2.5)	1.2 (0.2-2.2)	0.5 (-0.0-1.0)	0.8 (0.2-1.4)	1.2 (0.4-2.1)	0.4 (-0.0-0.8)	0.7 (0.1-1.3)	0.5 (0.0-1.0)	0.9 (-0.2-2.1)	1.4 (0.5-2.3)	0.9 (0.2-1.7)
Belo Horizonte	0.4 (-0.0-0.9)	0.9 (0.1-1.7)	0.5 (-0.1-1.2)	1.1 (0.1-2.1)	0.6 (0.0-1.2)	0.6 (0.1-1.1)	0.7 (0.1-1.2)	1.1 (0.3-1.9)	1.1 (0.0-2.1)	0.5 (-0.1-1.2)	0.3 (-0.0-0.5)
Boa Vista	0.7 (-0.0-1.4)	1.2 (0.2-2.1)	1.4 (0.4-2.4)	0.7 (0.1-1.4)	0.6 (-0.1-1.3)	0.5 (0.0-1.0)	0.8 (0.0-1.7)	1.0 (0.1-1.9)	2.8 (0.6-5.0)	0.8 (-0.0-1.5)	0.9 (-0.1-2.0)
Campo Grande	0.7 (-0.3-1.7)	0.4 (-0.1-0.9)	0.5 (-0.0-1.0)	0.6 (-0.1-1.2)	1.4 (0.2-2.5)	0.6 (0.1-1.1)	0.8 (0.2-1.5)	1.0 (0.2-1.7)	1.5 (0.2-2.8)	1.8 (0.6-2.9)	2.0 (0.3-3.6)
Cuiabá	0.6 (-0.1-1.2)	0.7 (0.0-1.5)	0.6 (-0.1-1.4)	0.6 (0.1-1.2)	1.1 (0.4-1.7)	1.3 (0.6-2.0)	1.0 (0.2-1.8)	2.1 (0.5-3.7)	2.5 (0.6-4.4)	0.5 (-0.2-1.1)	0.5 (0.1-0.9)
Curitiba	0.9 (0.1-1.7)	0.4 (-0.1-0.9)	0.6 (-0.0-1.2)	0.3 (-0.1-0.7)	0.9 (0.1-1.8)	0.6 (-0.0-1.1)	1.4 (0.4-2.4)	1.5 (0.2-2.7)	1.6 (-0.1-3.2)	0.7 (0.0-1.4)	1.8 (0.3-3.3)
Federal District	0.2 (-0.1-0.4)	0.3 (-0.0-0.6)	0.7 (0.0-1.4)	0.3 (-0.2-0.8)	0.5 (0.0-0.9)	0.1 (-0.1-0.2)	0.6 (-0.1-1.3)	0.8 (0.2-1.3)	1.4 (-0.0-2.8)	0.5 (-0.0-1.1)	0.9 (-0.0-1.8)
Florianópolis	0.2 (-0.0-0.5)	0.9 (0.2-1.6)	0.9 (0.2-1.7)	0.4 (-0.1-0.9)	0.6 (-0.0-1.2)	0.7 (0.1-1.4)	1.6 (0.5-2.8)	0.6 (-0.3-1.6)	2.8 (0.9-4.7)	1.7 (0.4-3.1)	1.3 (-0.2-2.7)
Fortaleza	0.7 (0.1-1.3)	0.3 (-0.0-0.6)	0.9 (0.3-1.6)	0.5 (-0.0-0.9)	1.6 (0.2-2.9)	0.5 (-0.0-1.0)	0.5 (-0.0-1.1)	1.2 (0.2-2.1)	2.0 (0.6-3.4)	1.3 (0.1-2.4)	1.3 (0.2-2.3)
Goiânia	0.5 (-0.1-1.1)	0.5 (0.1-1.0)	0.4 (-0.0-0.8)	0.7 (0.1-1.2)	0.6 (0.0-1.1)	0.5 (0.1-0.9)	1.2 (0.0-2.4)	0.6 (-0.1-1.3)	0.2 (-0.2-0.6)	0.3 (-0.2-0.8)	2.1 (0.3-3.9)
João Pessoa	1.1 (-0.1-2.3)	1.0 (-0.1-2.1)	1.6 (-0.0-3.2)	0.6 (0.1-1.2)	0.9 (-0.3-2.2)	1.5 (0.2-2.7)	1.1 (0.2-2.0)	1.0 (0.0-2.0)	1.0 (-0.1-2.1)	0.8 (-0.2-1.7)	2.5 (0.7-4.3)
Macapá	0.5 (0.0-1.0)	1.0 (0.0-2.0)	0.6 (-0.1-1.3)	1.1 (0.2-2.1)	0.8 (-0.0-1.6)	0.3 (-0.0-0.6)	0.9 (0.0-1.7)	1.5 (0.4-2.6)	1.0 (-0.5-2.4)	2.1 (0.6-3.6)	2.2 (-0.0-4.3)

Maceió	0.4 (-0.1-1.0)	0.8 (-0.2-1.9)	1.2 (-0.6-3.0)	2.0 (-0.3-4.3)	0.8 (-0.5-2.0)	1.1 (0.1-2.1)	0.7 (0.0-1.4)	0.8 (0.2-1.5)	0.5 (-0.0-1.1)	1.4 (-0.1-2.8)	1.6 (0.6-2.5)
Manaus	0.7 (0.1-1.2)	0.9 (0.2-1.5)	0.6 (0.1-1.1)	0.7 (0.1-1.3)	1.2 (0.1-2.3)	1.1 (0.3-1.8)	0.7 (0.1-1.3)	1.5 (0.4-2.6)	1.7 (0.3-3.1)	2.5 (0.8-4.1)	1.8 (0.6-2.9)
Natal	1.4 (0.3-2.5)	0.9 (0.2-1.5)	0.5 (0.0-0.9)	0.9 (0.2-1.5)	0.8 (0.1-1.5)	2.0 (0.5-3.6)	2.0 (0.3-3.7)	1.5 (0.5-2.6)	1.7 (0.5-2.9)	1.5 (0.4-2.7)	1.4 (-0.2-3.0)
Palmas	0.7 (-0.3-1.6)	0.8 (-0.5-2.1)	0.1 (-0.0-0.2)	0.8 (-0.1-1.7)	1.1 (-0.5-2.7)	0.3 (-0.0-0.7)	1.1 (-0.6-2.7)	1.0 (-0.1-2.2)	1.7 (-0.1-3.5)	0.6 (0.0-1.2)	1.2 (0.3-2.1)
Porto Alegre	1.1 (0.2-2.0)	0.6 (0.1-1.1)	0.9 (-0.1-1.9)	0.2 (-0.1-0.6)	1.2 (0.3-2.1)	0.8 (0.1-1.4)	0.4 (-0.0-0.8)	2.4 (0.7-4.2)	1.2 (0.2-2.2)	1.8 (0.4-3.1)	1.0 (0.1-2.0)
Porto Velho	0.2 (-0.0-0.5)	1.1 (0.2-2.0)	1.8 (0.3-3.2)	1.0 (0.1-1.8)	0.8 (0.2-1.5)	1.0 (0.2-1.8)	1.4 (0.1-2.7)	1.8 (0.3-3.3)	1.7 (-0.4-3.8)	1.2 (-0.0-2.5)	1.7 (-0.1-3.5)
Recife	0.6 (0.0-1.1)	0.3 (-0.2-0.9)	0.4 (-0.1-0.8)	2.3 (0.5-4.0)	0.8 (0.1-1.5)	1.0 (0.3-1.8)	0.5 (-0.0-1.1)	1.3 (0.3-2.4)	0.5 (-0.2-1.1)	1.0 (0.1-1.9)	1.2 (0.1-2.3)
Rio Branco	0.4 (-0.1-0.8)	1.5 (-0.3-3.2)	1.5 (-0.3-3.3)	0.5 (-0.1-1.1)	1.2 (-0.0-2.5)	1.1 (0.2-2.0)	1.3 (0.1-2.5)	1.0 (0.2-1.8)	2.8 (-1.4-7.0)	1.6 (0.3-2.8)	2.3 (1.0-3.7)
Rio de Janeiro	0.9 (0.1-1.7)	0.9 (-0.0-1.9)	0.3 (-0.1-0.7)	1.0 (0.2-1.8)	0.9 (0.1-1.7)	1.3 (-0.0-2.6)	1.2 (0.1-2.3)	0.5 (-0.1-1.1)	1.1 (-0.4-2.6)	0.4 (-0.3-1.2)	1.8 (0.4-3.1)
Salvador	0.4 (-0.0-0.8)	1.6 (-0.4-3.6)	0.4 (-0.0-0.7)	0.9 (0.2-1.7)	0.9 (-0.0-1.9)	0.8 (-0.3-2.0)	0.7 (-0.1-1.5)	0.4 (-0.1-1.0)	0.5 (-0.1-1.1)	0.7 (-0.0-1.4)	1.2 (0.2-2.1)
São Luís	0.7 (-0.3-1.7)	0.0 (-0.0-0.1)	0.1 (-0.1-0.3)	1.1 (-0.0-2.3)	0.4 (-0.0-0.9)	0.9 (-0.2-2.0)	0.8 (0.1-1.6)	0.5 (-0.2-1.2)	0.2 (-0.0-0.4)	0.7 (-0.0-1.5)	1.0 (0.1-1.8)
São Paulo	1.3 (0.2-2.4)	0.9 (0.1-1.7)	0.3 (-0.0-0.7)	0.2 (-0.0-0.5)	0.5 (-0.1-1.2)	0.8 (0.1-1.6)	1.3 (0.4-2.3)	1.8 (0.8-2.8)	1.0 (0.3-1.7)	1.5 (0.1-3.0)	0.8 (-0.1-1.7)
Teresina	0.2 (-0.2-0.6)	0.4 (-0.2-0.9)	0.4 (-0.1-0.9)	0.2 (-0.1-0.6)	0.4 (0.0-0.8)	1.0 (0.2-1.8)	0.2 (-0.1-0.5)	1.0 (0.2-1.8)	0.4 (-0.0-0.9)	0.7 (0.1-1.4)	0.8 (0.1-1.5)
Vitória	0.3 (-0.3-0.8)	0.7 (-0.0-1.4)	0.1 (-0.1-0.3)	0.6 (0.0-1.2)	1.1 (0.4-1.8)	1.1 (0.3-2.0)	0.5 (-0.1-1.1)	0.7 (0.1-1.4)	2.2 (0.7-3.6)	0.6 (-0.3-1.6)	0.3 (-0.1-0.7)
State capitals overall	0.9 (0.5-1.2)	0.8 (0.5-1.0)	0.5 (0.4-0.7)	0.7 (0.5-0.8)	0.8 (0.6-1.1)	0.8 (0.6-1.1)	1.0 (0.7-1.3)	1.2 (0.9-1.5)	1.2 (0.8-1.5)	1.1 (0.7-1.5)	1.2 (0.9-1.5)
