# Supplementary material for: Adult body weight trends in 27 urban populations of Brazil from 2006 to 2016: A population-based study
Source: PLoS One. 2019 Mar 6;14(3):e0213254. doi: 10.1371/journal.pone.0213254 (PMC6402686; doi:10.1371/journal.pone.0213254)
Supplement: S14 Table — Numbers in brackets show 95% confidence intervals. (PDF) [file pone.0213254.s014.pdf]

S14 Table. Age-standardized prevalence (%) of morbid obesity (BMI ≥ 40 kg/m²) in Brazil's state capitals, from 2006 to 2016, among women. Numbers in brackets show 95% confidence intervals.

State capital	2006	2007	2008	2009	2010	2011	2012	2013	2014	2015	2016
Aracaju	1.5 (0.7-2.3)	2.3 (1.1-3.4)	1.3 (0.4-2.2)	1.6 (0.7-2.4)	2.1 (1.1-3.1)	1.7 (0.9-2.5)	2.3 (1.0-3.6)	1.7 (0.8-2.6)	1.0 (0.3-1.6)	1.9 (0.6-3.1)	2.6 (1.3-3.8)
Belém	1.7 (0.7-2.6)	1.0 (0.2-1.8)	1.3 (0.6-2.1)	1.9 (0.8-3.0)	0.8 (0.3-1.2)	1.3 (0.5-2.1)	2.9 (1.4-4.4)	1.6 (0.6-2.6)	2.4 (0.9-3.8)	2.4 (1.3-3.5)	2.2 (1.1-3.3)
Belo Horizonte	1.1 (0.3-1.8)	0.9 (0.3-1.5)	1.3 (0.5-2.0)	1.5 (0.8-2.2)	1.4 (0.5-2.3)	1.1 (0.5-1.7)	1.5 (0.8-2.2)	1.6 (0.8-2.4)	1.2 (0.6-1.9)	2.0 (1.0-2.9)	1.8 (0.8-2.7)
Boa Vista	1.4 (0.5-2.2)	1.5 (0.8-2.2)	1.6 (0.5-2.7)	1.2 (0.3-2.2)	2.1 (0.9-3.2)	0.9 (0.1-1.7)	1.6 (0.6-2.6)	1.3 (0.0-2.5)	1.2 (0.2-2.1)	1.4 (0.6-2.2)	0.3 (0.1-0.5)
Campo Grande	1.0 (0.2-1.8)	1.8 (0.8-2.9)	1.2 (0.5-1.9)	2.1 (1.2-3.0)	1.7 (1.0-2.4)	2.0 (1.2-2.8)	2.0 (1.0-3.1)	1.3 (0.4-2.2)	2.5 (1.0-4.0)	2.8 (1.7-3.9)	1.9 (0.7-3.2)
Cuiabá	1.6 (0.4-2.8)	1.9 (0.9-2.8)	0.8 (0.3-1.3)	1.5 (0.6-2.3)	2.0 (1.1-2.9)	2.3 (1.3-3.3)	2.4 (0.5-4.2)	2.6 (1.5-3.7)	3.1 (1.4-4.9)	1.4 (0.7-2.1)	1.8 (1.0-2.6)
Curitiba	1.1 (0.5-1.7)	1.1 (0.1-2.1)	1.6 (0.7-2.6)	0.7 (0.2-1.3)	1.0 (0.4-1.5)	1.1 (0.5-1.7)	1.5 (0.7-2.3)	0.8 (0.1-1.4)	1.3 (0.3-2.4)	1.4 (0.6-2.3)	0.8 (-0.2-1.7)
Federal District	0.8 (0.1-1.6)	1.0 (0.4-1.7)	1.6 (0.8-2.3)	1.0 (0.1-1.8)	0.3 (-0.0-0.7)	1.7 (0.9-2.5)	1.9 (1.0-2.8)	1.6 (0.8-2.4)	3.0 (1.6-4.5)	0.6 (0.0-1.2)	0.7 (0.1-1.3)
Florianópolis	0.9 (0.3-1.4)	1.0 (0.3-1.6)	0.8 (0.2-1.3)	1.4 (0.4-2.4)	1.0 (0.4-1.5)	0.8 (0.2-1.4)	1.6 (0.5-2.7)	0.9 (0.3-1.4)	0.9 (0.4-1.4)	1.7 (0.7-2.7)	0.4 (0.1-0.7)
Fortaleza	1.0 (0.3-1.8)	1.5 (0.6-2.3)	1.6 (0.7-2.4)	1.7 (0.6-2.8)	1.0 (0.4-1.7)	1.9 (0.9-3.0)	0.8 (0.2-1.4)	1.8 (0.9-2.6)	3.5 (1.2-5.7)	1.4 (0.7-2.1)	2.1 (0.7-3.4)
Goiânia	1.0 (0.3-1.7)	1.0 (0.3-1.7)	0.8 (0.2-1.4)	0.9 (0.4-1.4)	1.1 (0.5-1.7)	0.9 (0.4-1.3)	0.8 (0.3-1.3)	1.1 (0.4-1.8)	1.6 (0.2-2.9)	1.3 (0.3-2.3)	1.4 (0.4-2.4)
João Pessoa	1.1 (0.5-1.7)	0.7 (0.1-1.3)	2.2 (0.7-3.7)	2.0 (0.7-3.3)	1.5 (0.3-2.7)	1.4 (0.3-2.6)	1.4 (0.2-2.7)	1.9 (0.5-3.3)	1.0 (0.2-1.8)	1.3 (0.6-2.0)	1.4 (0.6-2.2)
Macapá	1.8 (0.8-2.9)	2.4 (1.3-3.6)	2.2 (1.2-3.2)	2.9 (1.6-4.3)	5.2 (2.6-7.7)	2.5 (1.3-3.8)	2.6 (1.3-4.0)	1.6 (0.5-2.7)	2.9 (1.1-4.7)	2.6 (1.4-3.8)	2.3 (1.1-3.4)

Maceió	0.6 (0.1-1.1)	1.7 (0.7-2.7)	3.1 (1.2-5.0)	3.1 (1.6-4.6)	1.2 (0.3-2.1)	1.9 (0.8-3.0)	1.2 (0.4-2.0)	3.1 (1.7-4.4)	1.8 (0.7-3.0)	2.6 (1.4-3.9)	2.8 (1.0-4.5)
Manaus	2.2 (1.2-3.3)	1.5 (0.8-2.3)	1.6 (0.7-2.4)	2.4 (1.3-3.6)	1.5 (0.6-2.5)	2.0 (1.0-3.0)	3.0 (1.6-4.5)	1.7 (0.8-2.6)	2.1 (1.0-3.1)	3.7 (1.7-5.8)	1.6 (0.7-2.5)
Natal	1.3 (0.5-2.2)	2.0 (0.8-3.2)	1.0 (0.2-1.7)	2.1 (0.9-3.3)	2.0 (1.0-2.9)	2.0 (0.9-3.0)	1.7 (0.7-2.6)	1.2 (0.4-1.9)	2.1 (1.0-3.2)	2.3 (1.2-3.3)	2.1 (1.1-3.2)
Palmas	1.3 (0.4-2.2)	1.1 (0.4-1.8)	1.8 (0.7-3.0)	0.7 (-0.0-1.4)	1.9 (0.6-3.3)	1.4 (0.4-2.4)	2.5 (0.6-4.4)	1.6 (0.5-2.6)	1.6 (0.6-2.6)	0.4 (-0.0-0.9)	1.5 (0.5-2.6)
Porto Alegre	1.3 (0.5-2.2)	0.9 (0.2-1.5)	1.8 (0.7-2.9)	2.1 (0.9-3.2)	1.5 (0.7-2.4)	1.2 (0.5-2.0)	1.1 (0.5-1.8)	0.8 (0.2-1.4)	1.6 (0.3-3.0)	1.9 (0.7-3.1)	2.1 (0.7-3.5)
Porto Velho	1.1 (0.4-1.9)	2.1 (1.1-3.2)	1.3 (0.5-2.1)	2.6 (1.4-3.8)	3.0 (1.7-4.3)	3.5 (2.0-5.0)	2.3 (0.9-3.6)	1.7 (0.6-2.7)	1.4 (0.4-2.5)	3.0 (1.7-4.4)	1.6 (0.6-2.5)
Recife	1.2 (0.5-1.9)	1.9 (0.9-2.9)	1.8 (0.7-2.9)	1.0 (0.4-1.6)	2.4 (1.2-3.7)	1.3 (0.6-2.0)	2.4 (1.1-3.8)	2.0 (1.0-2.9)	2.4 (1.3-3.5)	1.5 (0.8-2.2)	2.3 (0.9-3.8)
Rio Branco	1.6 (0.7-2.4)	1.8 (0.8-2.7)	3.7 (1.6-5.8)	2.5 (1.3-3.7)	2.8 (0.6-4.9)	2.7 (1.6-3.9)	1.8 (0.7-2.9)	1.4 (0.6-2.2)	1.3 (0.5-2.1)	3.2 (1.7-4.8)	2.4 (1.4-3.5)
Rio de Janeiro	0.9 (0.3-1.4)	1.3 (0.2-2.3)	1.8 (0.9-2.8)	1.7 (0.8-2.6)	1.8 (1.0-2.7)	1.3 (0.6-2.1)	2.3 (0.9-3.7)	2.6 (1.5-3.7)	2.6 (1.0-4.2)	3.4 (1.2-5.7)	3.2 (1.1-5.4)
Salvador	1.2 (0.5-1.9)	1.8 (0.9-2.6)	2.3 (1.2-3.4)	2.0 (1.0-2.9)	2.7 (1.5-3.8)	1.5 (0.8-2.2)	2.0 (1.0-2.9)	2.2 (1.2-3.1)	2.5 (1.2-3.9)	1.4 (0.8-2.0)	2.7 (1.3-4.0)
São Luís	0.9 (0.2-1.7)	2.0 (0.7-3.4)	0.5 (0.1-0.9)	1.2 (0.3-2.2)	1.5 (0.6-2.4)	1.7 (0.7-2.7)	1.6 (0.8-2.4)	1.6 (0.6-2.6)	1.7 (0.6-2.8)	0.9 (0.3-1.5)	0.8 (-0.1-1.7)
São Paulo	1.6 (0.8-2.4)	1.0 (0.3-1.6)	0.3 (0.0-0.6)	1.6 (0.7-2.4)	1.3 (0.6-2.0)	1.1 (0.4-1.8)	2.0 (1.1-3.0)	1.3 (0.7-1.9)	1.0 (0.4-1.6)	2.9 (1.2-4.6)	2.0 (0.9-3.0)
Teresina	1.1 (0.3-2.0)	1.1 (0.2-1.9)	0.8 (0.2-1.4)	0.9 (0.3-1.6)	1.9 (0.8-3.0)	0.6 (0.2-1.1)	0.8 (0.0-1.6)	1.9 (0.6-3.2)	2.7 (0.7-4.7)	2.0 (0.9-3.0)	1.6 (0.3-2.9)
Vitória	1.2 (0.5-2.0)	0.5 (0.1-0.9)	1.3 (0.4-2.3)	1.5 (0.7-2.3)	1.4 (0.4-2.4)	1.4 (0.6-2.1)	1.3 (0.6-2.0)	1.4 (0.4-2.4)	1.7 (0.7-2.7)	2.2 (1.0-3.4)	0.9 (0.3-1.6)
State capitals overall	1.2 (1.0-1.5)	1.2 (1.0-1.5)	1.3 (1.1-1.5)	1.6 (1.3-1.9)	1.5 (1.3-1.8)	1.4 (1.1-1.6)	1.9 (1.5-2.2)	1.7 (1.4-2.0)	1.9 (1.5-2.2)	2.2 (1.7-2.7)	2.0 (1.6-2.4)
