# Supplementary material for: Adult body weight trends in 27 urban populations of Brazil from 2006 to 2016: A population-based study
Source: PLoS One. 2019 Mar 6;14(3):e0213254. doi: 10.1371/journal.pone.0213254 (PMC6402686; doi:10.1371/journal.pone.0213254)
Supplement: S15 Table — Numbers in brackets show 95% confidence intervals. (PDF) [file pone.0213254.s015.pdf]

**S15 Table. Age-standardized prevalence (%) of overweight (BMI ≥ 25 kg/m²) in Brazil's state capitals, from 2006 to 2016, among men.** Numbers in brackets show 95% confidence intervals.

| State capital | 2006 | 2007 | 2008 | 2009 | 2010 | 2011 | 2012 | 2013 | 2014 | 2015 | 2016 |
|---|---|---|---|---|---|---|---|---|---|---|---|
| Aracaju | 48.6 (44.0-53.2) | 43.7 (39.1-48.3) | 48.5 (43.9-53.1) | 53.5 (49.0-58.1) | 52.3 (47.5-57.1) | 48.6 (44.2-53.1) | 60.0 (55.2-64.7) | 50.9 (45.9-56.0) | 54.8 (49.7-59.8) | 56.0 (51.3-60.6) | 58.3 (53.9-62.7) |
| Belém | 50.3 (46.0-54.6) | 52.0 (47.9-56.1) | 53.6 (49.3-57.8) | 52.2 (47.9-56.5) | 52.8 (48.3-57.4) | 52.2 (47.6-56.8) | 57.5 (52.8-62.1) | 55.2 (50.6-59.8) | 62.6 (57.1-68.1) | 58.4 (54.2-62.7) | 61.2 (56.6-65.9) |
| Belo Horizonte | 45.6 (41.7-49.5) | 47.3 (43.2-51.4) | 47.0 (43.0-51.1) | 47.8 (43.8-51.9) | 48.6 (44.5-52.6) | 49.9 (46.0-53.8) | 52.4 (48.0-56.7) | 47.6 (43.5-51.8) | 48.7 (43.8-53.6) | 54.5 (50.3-58.8) | 52.2 (48.0-56.4) |
| Boa Vista | 50.7 (46.3-55.1) | 49.3 (44.6-54.0) | 49.0 (44.2-53.8) | 56.0 (51.2-60.8) | 52.7 (48.4-57.0) | 58.4 (53.7-63.2) | 54.5 (49.7-59.3) | 55.9 (50.8-61.0) | 59.5 (54.4-64.7) | 64.4 (59.2-69.6) | 61.2 (56.5-65.8) |
| Campo Grande | 49.6 (45.5-53.7) | 51.8 (47.5-56.1) | 54.2 (49.9-58.6) | 52.3 (48.1-56.5) | 54.9 (50.9-58.8) | 56.8 (52.8-60.7) | 61.3 (56.9-65.6) | 55.5 (50.5-60.5) | 58.5 (53.1-63.9) | 59.4 (55.2-63.7) | 62.9 (58.2-67.6) |
| Cuiabá | 50.5 (46.1-54.8) | 56.2 (52.1-60.3) | 52.4 (48.1-56.7) | 53.1 (49.0-57.2) | 53.3 (49.1-57.5) | 56.6 (52.6-60.7) | 58.2 (53.8-62.6) | 59.0 (54.3-63.6) | 58.8 (53.9-63.8) | 60.2 (54.3-66.1) | 63.1 (59.0-67.2) |
| Curitiba | 52.0 (48.2-55.7) | 49.8 (45.9-53.7) | 51.5 (47.8-55.1) | 52.7 (48.7-56.7) | 56.1 (52.2-60.0) | 57.0 (53.2-60.7) | 55.6 (51.2-60.0) | 57.1 (52.5-61.8) | 55.7 (50.5-60.8) | 57.4 (53.4-61.4) | 59.5 (54.4-64.6) |
| Federal District | 51.1 (45.7-56.6) | 44.6 (40.4-48.7) | 48.5 (44.4-52.6) | 43.5 (37.4-49.5) | 53.3 (45.9-60.7) | 53.8 (49.9-57.8) | 50.3 (46.0-54.5) | 55.0 (50.7-59.3) | 56.5 (51.4-61.5) | 53.2 (47.1-59.2) | 52.1 (46.4-57.7) |
| Florianópolis | 51.8 (47.8-55.9) | 52.3 (48.3-56.4) | 49.0 (45.1-52.9) | 53.7 (49.9-57.5) | 51.6 (47.6-55.6) | 56.7 (52.6-60.7) | 51.0 (46.7-55.4) | 56.9 (52.4-61.3) | 58.1 (53.0-63.2) | 61.8 (57.2-66.3) | 56.8 (51.5-62.1) |
| Fortaleza | 49.0 (44.5-53.5) | 50.3 (45.7-54.8) | 51.9 (47.3-56.5) | 50.4 (45.6-55.3) | 56.2 (51.7-60.8) | 58.1 (53.8-62.4) | 56.5 (51.6-61.4) | 54.7 (50.3-59.1) | 63.3 (58.3-68.4) | 63.1 (59.0-67.2) | 61.5 (56.9-66.2) |
| Goiânia | 44.8 (40.9-48.7) | 45.9 (42.0-49.7) | 48.7 (44.8-52.7) | 49.2 (45.2-53.3) | 52.6 (48.7-56.5) | 52.9 (48.9-56.8) | 52.6 (48.5-56.7) | 51.7 (47.6-55.9) | 52.6 (48.2-57.0) | 55.0 (48.0-62.0) | 51.9 (47.2-56.7) |
| João Pessoa | 49.7 (45.5-53.9) | 53.0 (48.4-57.6) | 51.4 (46.7-56.2) | 46.6 (41.6-51.5) | 52.9 (47.5-58.3) | 57.4 (52.9-61.9) | 56.0 (50.6-61.3) | 59.4 (54.3-64.5) | 57.0 (51.8-62.3) | 53.3 (48.8-57.9) | 59.6 (54.3-64.9) |
| Macapá | 49.8 (45.5-54.2) | 50.8 (46.3-55.4) | 55.7 (50.9-60.6) | 50.9 (45.9-55.9) | 56.7 (51.9-61.5) | 56.8 (52.0-61.6) | 54.1 (48.9-59.3) | 61.6 (55.8-67.3) | 55.6 (50.0-61.2) | 56.9 (52.0-61.9) | 58.5 (52.7-64.3) |

| | | | | | | | | | | | |
|---|---|---|---|---|---|---|---|---|---|---|---|
| Maceió | 44.0 (39.7-48.3) | 49.0 (43.7-54.2) | 50.5 (45.4-55.7) | 47.0 (41.9-52.0) | 53.0 (48.0-58.0) | 58.3 (53.7-62.9) | 57.2 (51.9-62.4) | 58.6 (53.7-63.5) | 53.1 (47.5-58.7) | 54.3 (49.8-58.9) | 56.5 (51.5-61.4) |
| Manaus | 53.7 (49.6-57.8) | 54.1 (50.0-58.2) | 47.6 (43.4-51.8) | 51.9 (47.2-56.6) | 54.7 (50.5-58.9) | 56.5 (52.3-60.7) | 54.2 (48.9-59.5) | 55.7 (51.2-60.3) | 61.3 (56.3-66.3) | 68.4 (63.5-73.3) | 61.5 (56.5-66.4) |
| Natal | 51.8 (47.5-56.0) | 51.1 (46.6-55.6) | 49.2 (44.5-53.8) | 51.8 (47.4-56.2) | 49.3 (44.6-54.0) | 56.0 (51.5-60.5) | 55.7 (50.8-60.6) | 57.0 (52.4-61.6) | 53.0 (47.3-58.7) | 57.6 (52.9-62.3) | 62.0 (57.4-66.7) |
| Palmas | 49.1 (44.5-53.7) | 42.1 (37.5-46.7) | 49.0 (44.1-53.9) | 49.6 (45.2-54.0) | 50.5 (46.0-55.0) | 48.2 (43.8-52.6) | 55.5 (50.6-60.4) | 58.7 (53.1-64.4) | 56.4 (51.6-61.3) | 56.7 (52.1-61.2) | 55.1 (50.9-59.2) |
| Porto Alegre | 57.2 (53.0-61.4) | 50.1 (45.7-54.4) | 55.2 (51.1-59.4) | 49.5 (45.4-53.5) | 55.3 (51.0-59.7) | 59.1 (54.8-63.4) | 59.9 (55.0-64.8) | 61.8 (57.2-66.5) | 62.1 (56.5-67.7) | 64.5 (60.2-68.9) | 61.5 (56.9-66.1) |
| Porto Velho | 47.7 (43.6-51.8) | 50.4 (45.9-54.9) | 50.1 (45.6-54.6) | 57.5 (53.2-61.8) | 53.8 (49.5-58.1) | 52.1 (47.8-56.4) | 56.7 (51.9-61.6) | 58.5 (54.0-63.0) | 64.1 (58.9-69.3) | 55.1 (50.0-60.3) | 61.6 (56.4-66.9) |
| Recife | 47.5 (43.2-51.9) | 47.6 (43.1-52.1) | 49.3 (44.7-54.0) | 52.3 (47.9-56.8) | 55.7 (51.3-60.2) | 53.4 (48.8-57.9) | 54.7 (50.0-59.4) | 52.5 (47.8-57.3) | 52.4 (47.5-57.3) | 53.0 (48.6-57.3) | 59.3 (54.8-63.8) |
| Rio Branco | 48.1 (43.8-52.5) | 47.9 (42.8-53.0) | 55.7 (50.6-60.8) | 52.3 (47.1-57.4) | 57.4 (52.7-62.1) | 56.2 (51.8-60.7) | 57.7 (52.3-63.0) | 58.1 (52.7-63.5) | 61.4 (55.5-67.4) | 59.3 (54.1-64.4) | 66.2 (62.2-70.2) |
| Rio de Janeiro | 51.0 (46.8-55.1) | 52.1 (48.0-56.2) | 49.8 (45.6-54.0) | 53.1 (48.6-57.5) | 53.7 (49.4-58.0) | 56.7 (52.3-61.0) | 54.5 (49.8-59.3) | 58.0 (53.6-62.3) | 58.8 (53.6-63.9) | 57.2 (51.7-62.7) | 58.7 (53.6-63.9) |
| Salvador | 43.5 (39.4-47.5) | 44.2 (40.1-48.4) | 43.9 (39.7-48.0) | 47.3 (43.1-51.5) | 42.4 (38.3-46.4) | 46.7 (42.6-50.7) | 46.4 (41.8-50.9) | 49.3 (45.0-53.5) | 55.1 (50.2-59.9) | 54.9 (50.2-59.6) | 53.2 (48.7-57.7) |
| São Luís | 41.4 (37.2-45.7) | 45.5 (41.1-49.9) | 48.4 (43.7-53.1) | 47.7 (43.4-52.0) | 47.5 (43.2-51.8) | 44.7 (40.3-49.2) | 52.2 (47.1-57.2) | 46.9 (42.1-51.7) | 52.7 (47.6-57.8) | 54.7 (50.0-59.5) | 51.6 (46.7-56.6) |
| São Paulo | 45.5 (41.5-49.5) | 49.3 (45.3-53.3) | 52.9 (48.9-57.0) | 50.9 (46.7-55.2) | 53.2 (49.2-57.2) | 51.7 (47.9-55.6) | 55.0 (50.7-59.4) | 54.4 (50.5-58.2) | 55.3 (50.5-60.1) | 58.5 (54.4-62.6) | 57.5 (53.6-61.4) |
| Teresina | 45.4 (41.2-49.6) | 45.2 (40.7-49.6) | 42.5 (38.2-46.9) | 45.2 (40.5-49.8) | 54.1 (48.9-59.4) | 51.6 (47.1-56.1) | 53.6 (48.1-59.1) | 54.8 (49.8-59.8) | 52.5 (46.8-58.2) | 54.6 (50.1-59.1) | 58.6 (53.9-63.2) |
| Vitória | 47.2 (43.3-51.1) | 52.4 (48.4-56.3) | 50.2 (46.2-54.2) | 50.2 (46.2-54.2) | 54.8 (50.7-59.0) | 50.9 (46.8-55.0) | 55.5 (51.0-60.0) | 52.9 (48.5-57.3) | 54.4 (49.6-59.2) | 53.2 (48.1-58.4) | 54.3 (49.4-59.1) |
| State capitals overall | 48.1 (46.8-49.5) | 49.4 (48.0-50.7) | 50.4 (49.1-51.7) | 50.4 (49.0-51.8) | 52.7 (51.4-54.1) | 53.6 (52.3-54.9) | 54.4 (53.0-55.9) | 54.8 (53.5-56.2) | 56.5 (54.9-58.1) | 57.7 (56.2-59.1) | 57.5 (56.1-59.0) |
