# Supplementary material for: Adult body weight trends in 27 urban populations of Brazil from 2006 to 2016: A population-based study
Source: PLoS One. 2019 Mar 6;14(3):e0213254. doi: 10.1371/journal.pone.0213254 (PMC6402686; doi:10.1371/journal.pone.0213254)
Supplement: S16 Table — Numbers in brackets show 95% confidence intervals. (PDF) [file pone.0213254.s016.pdf]

S16 Table. Age-standardized prevalence (%) of overweight (BMI ≥ 25 kg/m²) in Brazil's state capitals, from 2006 to 2016, among women. Numbers in brackets show 95% confidence intervals.

State capital	2006	2007	2008	2009	2010	2011	2012	2013	2014	2015	2016
Aracaju	35.9 (32.9-38.8)	36.2 (33.1-39.4)	41.4 (38.0-44.7)	40.7 (37.3-44.1)	46.0 (42.7-49.3)	43.1 (40.0-46.3)	43.8 (40.2-47.4)	46.4 (42.9-49.9)	46.1 (42.4-49.8)	46.8 (43.6-50.0)	52.4 (48.8-56.0)
Belém	34.0 (31.1-37.0)	35.2 (31.8-38.6)	39.7 (36.1-43.2)	36.9 (33.6-40.2)	40.3 (37.0-43.5)	43.2 (39.9-46.5)	43.5 (39.7-47.3)	46.4 (42.8-50.0)	46.4 (42.3-50.6)	48.0 (44.6-51.3)	46.5 (42.9-50.2)
Belo Horizonte	32.2 (29.4-35.1)	34.3 (31.4-37.2)	37.6 (34.5-40.6)	38.6 (35.5-41.8)	38.1 (35.0-41.2)	40.2 (36.9-43.4)	42.8 (39.4-46.2)	43.8 (40.3-47.4)	45.5 (41.6-49.5)	43.5 (40.1-46.8)	42.9 (39.3-46.4)
Boa Vista	36.8 (33.4-40.1)	37.6 (34.2-41.0)	42.7 (39.0-46.3)	44.1 (40.5-47.8)	47.5 (43.9-51.0)	44.3 (40.5-48.0)	44.3 (40.5-48.1)	47.3 (43.6-51.0)	45.2 (40.9-49.6)	54.0 (49.8-58.2)	46.7 (42.8-50.7)
Campo Grande	37.6 (34.5-40.6)	39.5 (36.1-42.8)	39.4 (36.2-42.5)	45.1 (41.8-48.3)	47.5 (44.3-50.6)	44.7 (41.5-47.9)	50.7 (47.0-54.5)	48.4 (44.6-52.3)	48.5 (44.1-53.0)	56.1 (52.5-59.8)	51.9 (47.6-56.1)
Cuiabá	38.5 (35.1-41.9)	41.9 (38.6-45.2)	45.1 (41.8-48.4)	41.7 (38.2-45.1)	46.2 (42.9-49.4)	45.4 (42.2-48.7)	45.3 (41.5-49.0)	50.0 (46.5-53.6)	50.8 (46.8-54.8)	47.1 (42.5-51.8)	49.4 (45.8-53.0)
Curitiba	36.2 (33.4-39.0)	39.1 (36.1-42.0)	41.3 (38.2-44.4)	40.0 (36.9-43.0)	41.4 (38.3-44.5)	42.4 (39.3-45.5)	45.9 (42.2-49.6)	45.7 (41.8-49.5)	50.0 (45.3-54.7)	48.5 (44.7-52.3)	46.6 (41.8-51.4)
Federal District	34.4 (30.9-37.9)	38.5 (35.4-41.6)	37.3 (34.3-40.3)	35.1 (30.7-39.5)	39.0 (32.3-45.8)	44.3 (41.0-47.5)	44.4 (41.0-47.8)	43.2 (39.9-46.6)	43.7 (39.8-47.7)	42.8 (37.9-47.8)	45.4 (40.7-50.1)
Florianópolis	32.4 (29.4-35.3)	33.4 (30.2-36.5)	35.1 (32.1-38.2)	38.2 (34.9-41.4)	38.5 (35.4-41.5)	37.4 (34.2-40.7)	45.3 (41.2-49.4)	38.6 (34.9-42.3)	40.9 (36.7-45.2)	39.7 (35.7-43.7)	38.7 (34.3-43.2)
Fortaleza	34.9 (31.7-38.1)	38.7 (35.2-42.1)	40.9 (37.5-44.3)	44.6 (41.2-47.9)	49.2 (45.7-52.6)	46.2 (42.8-49.6)	49.3 (45.5-53.1)	47.0 (43.5-50.5)	48.9 (45.0-52.8)	48.3 (44.8-51.8)	51.5 (47.6-55.4)
Goiânia	32.8 (29.9-35.7)	34.2 (31.3-37.2)	36.6 (33.6-39.6)	39.0 (35.8-42.1)	37.9 (34.8-40.9)	40.5 (37.4-43.5)	45.8 (42.3-49.2)	42.9 (39.5-46.4)	45.5 (41.6-49.4)	38.0 (33.2-42.8)	44.1 (40.6-47.6)
João Pessoa	37.4 (34.1-40.7)	40.0 (36.5-43.4)	44.3 (40.5-48.0)	42.8 (39.4-46.3)	41.0 (37.3-44.7)	43.2 (39.6-46.9)	46.0 (42.2-49.9)	44.0 (40.3-47.6)	45.3 (41.3-49.3)	52.9 (49.0-56.8)	52.3 (47.9-56.7)
Macapá	38.5 (35.1-41.9)	40.6 (37.0-44.1)	45.2 (41.6-48.9)	44.6 (40.8-48.4)	44.7 (40.8-48.5)	48.6 (44.8-52.4)	50.9 (47.0-54.8)	44.8 (41.0-48.6)	49.0 (44.8-53.1)	48.6 (44.8-52.5)	49.6 (45.7-53.4)

Maceió	36.9 (33.4-40.4)	36.2 (32.8-39.6)	42.4 (38.7-46.1)	40.2 (36.7-43.7)	43.1 (39.5-46.7)	44.4 (40.8-48.0)	47.6 (43.5-51.7)	46.3 (42.5-50.1)	51.5 (47.0-56.1)	51.3 (47.5-55.0)	52.0 (48.1-56.0)
Manaus	38.2 (35.1-41.3)	41.8 (38.4-45.1)	41.6 (38.3-45.0)	44.8 (41.5-48.0)	48.3 (44.9-51.7)	51.5 (48.1-54.8)	52.0 (48.0-56.0)	51.5 (47.7-55.2)	53.2 (48.9-57.6)	54.7 (50.5-59.0)	51.9 (47.9-55.8)
Natal	36.6 (33.5-39.8)	41.2 (37.9-44.5)	40.9 (37.4-44.4)	42.3 (39.0-45.6)	48.6 (45.2-52.0)	49.0 (45.7-52.3)	48.2 (44.3-52.0)	47.1 (43.4-50.8)	49.2 (45.3-53.2)	51.1 (47.3-54.9)	50.0 (46.1-54.0)
Palmas	32.5 (28.7-36.4)	33.9 (30.3-37.4)	32.4 (28.5-36.3)	35.4 (31.7-39.1)	38.4 (35.1-41.7)	39.6 (36.0-43.2)	41.1 (37.3-44.9)	43.4 (39.5-47.3)	46.0 (42.1-49.8)	44.1 (40.2-48.0)	42.4 (38.9-46.0)
Porto Alegre	39.3 (36.0-42.5)	37.4 (34.2-40.6)	40.1 (36.6-43.6)	42.7 (39.1-46.3)	47.2 (43.7-50.7)	47.7 (44.1-51.3)	47.6 (43.6-51.7)	44.3 (40.0-48.6)	46.6 (41.5-51.8)	47.4 (42.8-52.0)	44.7 (40.2-49.1)
Porto Velho	37.9 (34.5-41.3)	40.0 (36.4-43.6)	43.1 (39.7-46.6)	43.4 (39.9-46.9)	47.7 (44.1-51.2)	45.2 (41.8-48.7)	48.4 (44.6-52.1)	48.8 (44.7-52.9)	50.6 (45.7-55.5)	53.1 (49.0-57.2)	50.1 (46.1-54.0)
Recife	40.0 (36.7-43.3)	41.0 (37.5-44.5)	39.7 (36.4-43.0)	41.6 (38.1-45.1)	44.2 (40.8-47.5)	44.0 (40.6-47.3)	50.3 (46.3-54.3)	46.9 (43.2-50.6)	53.0 (49.1-56.9)	51.3 (47.7-54.8)	50.8 (47.0-54.7)
Rio Branco	43.6 (40.2-47.0)	40.7 (37.2-44.2)	44.6 (40.7-48.5)	45.8 (42.2-49.4)	47.1 (43.5-50.6)	48.6 (45.1-52.0)	50.7 (46.7-54.7)	50.2 (46.1-54.2)	50.2 (45.7-54.7)	54.3 (50.2-58.4)	56.0 (52.6-59.4)
Rio de Janeiro	41.0 (38.0-44.1)	39.9 (36.6-43.1)	40.5 (37.2-43.8)	42.6 (39.3-45.9)	46.3 (42.8-49.8)	42.4 (39.0-45.7)	46.9 (43.1-50.7)	44.2 (40.7-47.6)	47.7 (43.3-52.0)	52.1 (47.5-56.7)	49.4 (44.9-53.9)
Salvador	39.6 (36.4-42.8)	39.1 (35.9-42.3)	40.9 (37.7-44.2)	42.8 (39.7-45.9)	41.6 (38.6-44.7)	44.3 (41.0-47.6)	48.0 (44.4-51.6)	43.7 (40.4-46.9)	47.5 (43.7-51.3)	48.9 (45.2-52.6)	50.8 (47.2-54.4)
São Luís	32.3 (29.2-35.3)	33.5 (30.2-36.7)	34.4 (31.3-37.5)	35.8 (32.5-39.1)	37.9 (34.8-41.0)	41.0 (37.8-44.3)	39.9 (36.4-43.4)	39.4 (35.9-42.8)	42.2 (38.4-46.1)	42.5 (39.1-45.8)	45.4 (41.5-49.3)
São Paulo	39.6 (36.7-42.5)	37.2 (34.2-40.2)	39.4 (36.4-42.3)	41.7 (38.4-45.0)	43.8 (40.6-47.1)	42.3 (39.1-45.4)	45.7 (42.3-49.2)	45.5 (42.3-48.8)	46.8 (43.0-50.5)	50.2 (46.6-53.8)	47.6 (44.2-51.1)
Teresina	32.3 (29.1-35.6)	35.3 (32.0-38.5)	36.7 (33.2-40.3)	37.8 (34.5-41.1)	36.3 (33.1-39.5)	40.8 (37.3-44.3)	40.8 (36.9-44.6)	44.7 (41.1-48.4)	43.7 (39.6-47.8)	45.4 (41.7-49.1)	45.1 (41.3-49.0)
Vitória	33.2 (30.4-36.0)	32.4 (29.6-35.3)	37.8 (34.7-40.9)	38.9 (35.8-42.1)	38.5 (35.3-41.7)	41.7 (38.4-45.0)	40.3 (36.8-43.9)	43.4 (39.8-47.0)	46.7 (42.7-50.8)	45.1 (41.0-49.2)	43.2 (39.3-47.1)
State capitals overall	37.8 (36.8-38.8)	37.9 (36.9-38.9)	39.8 (38.8-40.8)	41.2 (40.2-42.3)	43.4 (42.3-44.5)	43.4 (42.4-44.5)	46.3 (45.2-47.4)	45.4 (44.3-46.5)	47.3 (46.0-48.5)	48.6 (47.4-49.8)	48.2 (47.0-49.4)
