# Supplementary material for: Adult body weight trends in 27 urban populations of Brazil from 2006 to 2016: A population-based study
Source: PLoS One. 2019 Mar 6;14(3):e0213254. doi: 10.1371/journal.pone.0213254 (PMC6402686; doi:10.1371/journal.pone.0213254)
Supplement: S17 Table — Numbers in brackets show 95% confidence intervals. (PDF) [file pone.0213254.s017.pdf]

S17 Table. Age-standardized prevalence (%) of obesity (BMI ≥ 30 kg/m²) in Brazil's state capitals, from 2006 to 2016, among men. Numbers in brackets show 95% confidence intervals.

State capital	2006	2007	2008	2009	2010	2011	2012	2013	2014	2015	2016
Aracaju	15.2 (11.6-18.8)	10.8 (7.9-13.6)	13.4 (10.4-16.4)	18.9 (14.9-23.0)	16.7 (12.9-20.4)	14.6 (11.4-17.8)	19.7 (15.7-23.6)	15.8 (12.4-19.1)	18.7 (14.7-22.8)	17.6 (14.2-21.0)	20.7 (16.9-24.5)
Belém	17.1 (13.7-20.4)	16.8 (13.7-19.9)	14.6 (11.4-17.7)	12.6 (9.8-15.4)	17.4 (14.1-20.8)	15.9 (13.0-18.9)	17.3 (13.6-21.1)	16.2 (13.1-19.3)	21.9 (17.2-26.7)	19.9 (16.3-23.5)	21.5 (17.4-25.6)
Belo Horizonte	9.7 (7.4-12.1)	10.7 (8.2-13.2)	11.1 (8.6-13.6)	10.3 (7.8-12.7)	12.1 (9.6-14.6)	12.9 (10.3-15.5)	13.3 (10.4-16.3)	13.5 (10.6-16.5)	14.9 (11.2-18.5)	15.3 (12.2-18.3)	14.4 (11.6-17.3)
Boa Vista	14.4 (11.2-17.6)	13.6 (10.5-16.7)	16.1 (11.6-20.6)	15.8 (12.3-19.3)	14.3 (11.1-17.5)	16.7 (13.2-20.3)	15.7 (12.3-19.2)	20.3 (16.5-24.1)	21.2 (16.9-25.5)	24.7 (19.4-30.1)	22.6 (17.4-27.8)
Campo Grande	13.1 (10.4-15.8)	16.8 (13.7-19.8)	14.2 (11.4-17.1)	16.0 (12.8-19.3)	16.0 (13.1-18.8)	18.9 (15.8-22.0)	19.6 (16.1-23.2)	15.1 (12.0-18.2)	18.8 (14.4-23.3)	19.9 (16.7-23.1)	21.4 (17.4-25.5)
Cuiabá	13.6 (10.8-16.5)	15.3 (12.5-18.1)	15.4 (12.5-18.2)	14.1 (11.4-16.8)	18.5 (15.3-21.8)	19.4 (16.3-22.5)	19.8 (16.0-23.6)	21.8 (17.5-26.0)	19.6 (15.5-23.8)	16.8 (13.0-20.6)	23.9 (20.2-27.7)
Curitiba	13.1 (10.4-15.7)	13.5 (10.8-16.2)	14.5 (11.8-17.1)	13.6 (10.9-16.4)	16.8 (13.9-19.6)	15.5 (12.7-18.3)	16.1 (13.0-19.2)	18.8 (15.0-22.6)	17.4 (13.4-21.4)	13.8 (11.3-16.3)	21.3 (17.0-25.7)
Federal District	11.6 (8.2-15.0)	10.6 (8.2-13.0)	10.6 (8.1-13.2)	8.8 (5.9-11.7)	11.4 (8.0-14.8)	13.8 (11.1-16.5)	13.7 (10.9-16.5)	16.1 (12.9-19.2)	15.7 (12.0-19.4)	14.1 (10.4-17.9)	17.2 (12.5-21.9)
Florianópolis	10.6 (8.1-13.0)	11.0 (8.6-13.4)	12.7 (10.1-15.3)	13.7 (10.9-16.4)	15.2 (12.3-18.2)	17.3 (14.2-20.3)	16.4 (13.1-19.6)	16.5 (13.2-19.7)	14.5 (10.9-18.0)	17.0 (13.2-20.8)	14.0 (10.4-17.7)
Fortaleza	13.9 (10.5-17.4)	14.6 (11.6-17.5)	16.6 (13.5-19.8)	15.4 (12.1-18.7)	20.4 (16.6-24.1)	20.6 (16.8-24.4)	18.9 (15.0-22.9)	19.6 (15.9-23.4)	21.5 (16.8-26.2)	21.7 (17.9-25.6)	20.4 (16.8-24.0)
Goiânia	9.9 (7.8-12.0)	12.4 (9.7-15.0)	10.4 (8.2-12.6)	11.1 (8.6-13.5)	12.9 (10.4-15.4)	13.2 (10.5-15.8)	12.3 (9.6-15.0)	18.2 (14.9-21.5)	13.8 (10.4-17.1)	14.6 (9.6-19.5)	18.3 (14.5-22.0)
João Pessoa	16.6 (13.2-20.0)	14.9 (11.8-18.1)	16.4 (12.9-20.0)	10.6 (8.2-13.1)	15.9 (12.3-19.5)	17.6 (14.3-21.0)	21.5 (17.0-25.9)	15.6 (12.4-18.7)	16.7 (12.6-20.7)	17.6 (14.0-21.1)	23.6 (19.0-28.2)
Macapá	17.5 (14.1-20.9)	20.2 (16.6-23.9)	14.9 (11.6-18.2)	17.2 (13.5-20.9)	17.9 (14.4-21.4)	20.3 (16.6-24.0)	15.7 (12.0-19.5)	23.4 (18.9-27.9)	16.2 (12.5-19.8)	22.2 (18.1-26.4)	20.3 (16.2-24.4)

Maceió	12.8 (9.8-15.8)	13.1 (9.9-16.3)	15.6 (11.8-19.3)	12.5 (8.9-16.1)	16.0 (12.4-19.5)	17.5 (13.9-21.1)	18.8 (14.6-23.1)	18.9 (15.1-22.6)	20.0 (15.0-25.0)	17.8 (14.3-21.3)	19.1 (15.3-22.9)
Manaus	14.2 (11.3-17.0)	14.4 (11.6-17.2)	15.0 (12.0-18.0)	16.3 (13.0-19.6)	18.2 (15.0-21.5)	20.8 (17.2-24.4)	19.2 (15.0-23.4)	19.1 (15.4-22.9)	18.6 (14.6-22.5)	30.2 (24.4-36.0)	19.6 (16.1-23.2)
Natal	16.3 (13.1-19.5)	15.9 (12.6-19.3)	13.4 (10.3-16.4)	15.3 (12.0-18.6)	14.7 (11.5-18.0)	19.4 (15.4-23.3)	20.4 (16.5-24.2)	19.1 (15.2-23.1)	15.9 (11.9-20.0)	19.7 (15.7-23.7)	19.4 (15.6-23.2)
Palmas	12.0 (8.9-15.1)	10.6 (7.7-13.5)	13.7 (10.3-17.2)	11.4 (8.9-14.0)	16.6 (12.9-20.3)	14.6 (11.6-17.6)	15.8 (11.9-19.8)	21.7 (15.7-27.6)	18.7 (14.8-22.6)	14.8 (11.6-17.9)	15.7 (12.8-18.7)
Porto Alegre	12.2 (9.5-14.8)	13.7 (10.9-16.5)	15.3 (12.2-18.3)	11.8 (9.3-14.3)	15.4 (12.3-18.5)	16.5 (13.4-19.5)	17.8 (14.2-21.5)	18.4 (14.7-22.1)	23.9 (19.2-28.5)	23.4 (19.3-27.5)	18.7 (15.1-22.3)
Porto Velho	13.1 (10.3-15.9)	15.5 (12.2-18.7)	15.3 (12.0-18.6)	19.9 (16.2-23.6)	15.0 (12.0-18.1)	18.0 (14.9-21.1)	19.1 (15.3-22.9)	19.1 (15.7-22.4)	20.3 (16.0-24.7)	18.1 (14.4-21.8)	22.1 (17.2-26.9)
Recife	11.9 (9.2-14.6)	12.7 (10.0-15.4)	14.5 (10.9-18.0)	15.7 (12.2-19.1)	19.3 (15.7-22.8)	14.6 (11.9-17.3)	17.2 (13.5-20.8)	16.3 (12.6-20.1)	15.5 (12.1-18.9)	15.5 (12.6-18.4)	20.0 (16.5-23.6)
Rio Branco	14.2 (11.2-17.1)	15.0 (11.4-18.6)	14.2 (10.6-17.8)	15.5 (12.0-19.0)	16.9 (13.5-20.3)	17.7 (14.1-21.4)	18.4 (14.2-22.6)	17.3 (13.4-21.2)	23.4 (17.2-29.5)	22.4 (17.8-27.0)	25.0 (21.3-28.8)
Rio de Janeiro	11.4 (8.9-13.9)	14.2 (11.4-17.1)	12.7 (9.8-15.7)	17.8 (14.4-21.1)	13.9 (11.0-16.8)	17.2 (13.9-20.5)	17.1 (13.5-20.7)	21.2 (17.5-24.8)	19.9 (15.8-24.1)	14.6 (10.5-18.6)	20.4 (16.2-24.6)
Salvador	9.8 (7.3-12.3)	11.2 (8.1-14.2)	10.4 (8.0-12.9)	11.1 (8.7-13.5)	9.9 (7.6-12.1)	12.7 (10.1-15.4)	10.1 (7.4-12.7)	12.9 (10.0-15.8)	16.0 (12.1-20.0)	14.4 (11.1-17.6)	17.6 (14.1-21.1)
São Luís	9.7 (7.0-12.5)	10.8 (8.0-13.6)	10.8 (7.4-14.2)	10.6 (8.1-13.0)	13.9 (10.9-16.9)	12.8 (10.0-15.7)	15.0 (11.4-18.5)	12.6 (9.4-15.8)	13.0 (9.7-16.3)	15.1 (11.1-19.0)	13.3 (10.5-16.2)
São Paulo	9.8 (7.4-12.2)	15.3 (12.4-18.2)	15.0 (12.1-18.0)	14.9 (11.8-18.0)	13.7 (11.0-16.4)	14.0 (11.4-16.7)	17.3 (14.1-20.5)	17.0 (14.2-19.9)	16.5 (13.2-19.9)	20.7 (17.3-24.1)	15.1 (12.3-17.9)
Teresina	11.1 (8.5-13.8)	11.8 (8.7-14.8)	12.4 (9.5-15.3)	12.6 (9.6-15.5)	14.6 (11.4-17.8)	13.2 (10.4-15.9)	17.1 (12.6-21.6)	17.9 (13.5-22.4)	16.1 (12.3-19.9)	16.7 (13.3-20.1)	17.5 (13.7-21.3)
Vitória	11.1 (8.5-13.7)	13.9 (11.2-16.6)	12.0 (9.5-14.5)	11.1 (8.7-13.4)	15.1 (12.3-17.9)	12.9 (10.2-15.6)	17.3 (13.8-20.7)	16.1 (12.9-19.2)	16.7 (13.3-20.1)	12.5 (9.3-15.7)	12.3 (9.4-15.3)
State capitals overall	11.7 (10.8-12.5)	13.9 (12.9-14.8)	13.7 (12.7-14.6)	14.1 (13.0-15.1)	14.6 (13.7-15.6)	15.6 (14.6-16.5)	16.5 (15.4-17.6)	17.6 (16.6-18.6)	17.7 (16.5-18.9)	18.2 (17.0-19.4)	18.1 (17.0-19.2)
