# Supplementary material for: Adult body weight trends in 27 urban populations of Brazil from 2006 to 2016: A population-based study
Source: PLoS One. 2019 Mar 6;14(3):e0213254. doi: 10.1371/journal.pone.0213254 (PMC6402686; doi:10.1371/journal.pone.0213254)
Supplement: S18 Table — Numbers in brackets show 95% confidence intervals. (PDF) [file pone.0213254.s018.pdf]

S18 Table. Age-standardized prevalence (%) of obesity (BMI ≥ 30 kg/m²) in Brazil's state capitals, from 2006 to 2016, among women. Numbers in brackets show 95% confidence intervals.

State capital	2006	2007	2008	2009	2010	2011	2012	2013	2014	2015	2016
Aracaju	12.7 (10.6-14.8)	13.1 (10.7-15.6)	14.8 (12.3-17.3)	14.4 (12.0-16.8)	15.3 (13.0-17.7)	17.1 (14.6-19.6)	16.5 (13.7-19.3)	17.9 (15.2-20.6)	16.4 (13.6-19.1)	17.2 (14.6-19.8)	19.5 (16.8-22.3)
Belém	10.9 (8.9-12.9)	13.2 (10.7-15.7)	13.5 (10.9-16.0)	14.7 (12.1-17.2)	14.4 (12.0-16.8)	13.1 (10.9-15.3)	15.2 (12.5-18.0)	15.3 (12.7-17.9)	20.0 (16.5-23.5)	17.9 (15.2-20.7)	17.0 (14.4-19.7)
Belo Horizonte	9.4 (7.5-11.3)	12.0 (9.8-14.2)	12.4 (10.2-14.6)	13.7 (11.5-15.9)	12.9 (10.7-15.1)	14.3 (12.0-16.7)	14.8 (12.4-17.2)	14.0 (11.7-16.3)	17.1 (14.1-20.1)	17.8 (15.3-20.4)	16.1 (13.6-18.5)
Boa Vista	12.7 (10.4-15.1)	12.9 (10.5-15.3)	14.9 (12.2-17.5)	14.4 (12.0-16.8)	17.3 (14.5-20.1)	17.6 (14.7-20.6)	17.0 (14.2-19.9)	17.3 (14.4-20.2)	17.4 (14.2-20.5)	21.8 (18.3-25.4)	16.3 (13.2-19.4)
Campo Grande	13.3 (11.0-15.6)	14.8 (12.4-17.1)	14.3 (12.1-16.4)	16.9 (14.5-19.4)	18.1 (15.8-20.5)	17.1 (14.8-19.5)	22.0 (18.8-25.1)	19.9 (16.8-23.0)	23.8 (20.0-27.5)	23.5 (20.5-26.5)	18.0 (14.8-21.3)
Cuiabá	12.6 (10.1-15.0)	14.0 (11.7-16.4)	14.0 (11.5-16.4)	15.8 (13.5-18.2)	18.3 (15.7-20.8)	17.7 (15.1-20.2)	18.6 (15.4-21.7)	22.3 (19.4-25.2)	22.9 (19.5-26.3)	17.8 (14.1-21.5)	19.8 (17.1-22.5)
Curitiba	12.3 (10.3-14.3)	12.9 (10.8-15.0)	13.3 (11.2-15.4)	11.7 (9.7-13.8)	16.8 (14.6-19.0)	15.3 (13.1-17.5)	15.5 (13.0-18.0)	15.9 (13.2-18.6)	19.5 (15.7-23.3)	17.2 (14.5-19.8)	15.4 (12.0-18.9)
Federal District	11.1 (8.7-13.5)	11.0 (9.0-12.9)	14.6 (12.3-16.9)	10.2 (7.4-13.0)	9.5 (6.4-12.7)	14.9 (12.6-17.2)	15.1 (12.7-17.5)	14.5 (12.2-16.7)	15.4 (12.7-18.1)	13.7 (10.6-16.8)	16.3 (12.5-20.1)
Florianópolis	10.4 (8.4-12.4)	11.2 (9.1-13.3)	11.2 (9.2-13.3)	13.9 (11.5-16.4)	13.5 (11.4-15.6)	13.2 (10.9-15.5)	14.8 (12.0-17.6)	13.2 (10.9-15.5)	13.3 (10.5-16.2)	14.4 (11.6-17.2)	13.8 (10.8-16.8)
Fortaleza	10.2 (8.1-12.3)	13.5 (11.0-16.1)	14.4 (11.8-16.9)	16.0 (13.3-18.7)	17.5 (14.6-20.4)	17.2 (14.6-19.9)	18.6 (15.4-21.8)	16.5 (14.0-19.1)	16.7 (13.5-19.8)	18.0 (15.4-20.6)	19.0 (15.7-22.2)
Goiânia	10.5 (8.6-12.3)	11.3 (9.4-13.3)	12.8 (10.8-14.8)	12.2 (10.1-14.3)	12.8 (10.7-14.9)	14.5 (12.3-16.7)	15.6 (13.2-18.0)	14.6 (12.3-16.9)	15.9 (12.8-19.0)	12.2 (8.7-15.6)	14.3 (11.7-16.9)
João Pessoa	12.8 (10.6-15.0)	12.7 (10.4-15.1)	15.4 (12.6-18.3)	16.4 (13.5-19.3)	14.2 (11.6-16.9)	16.1 (13.4-18.8)	18.5 (15.4-21.5)	18.1 (15.1-21.1)	16.3 (13.4-19.2)	21.2 (18.0-24.5)	19.5 (15.7-23.4)
Macapá	13.8 (11.3-16.3)	15.9 (13.2-18.6)	16.7 (13.8-19.6)	16.4 (13.4-19.3)	18.8 (15.4-22.2)	18.4 (15.4-21.4)	21.7 (18.1-25.2)	15.4 (12.9-18.0)	22.0 (18.4-25.6)	19.4 (16.5-22.4)	17.3 (14.7-19.9)

Maceió	13.9 (11.2-16.6)	12.8 (10.6-15.0)	14.1 (11.3-16.9)	15.2 (12.4-18.0)	14.4 (11.9-17.0)	17.6 (14.7-20.5)	20.4 (16.8-24.0)	17.8 (15.0-20.6)	19.6 (16.5-22.6)	20.9 (18.0-23.8)	21.4 (18.1-24.6)
Manaus	15.9 (13.3-18.4)	16.1 (13.6-18.6)	16.6 (13.9-19.2)	16.7 (14.2-19.3)	18.5 (15.8-21.2)	19.2 (16.5-21.8)	20.4 (17.4-23.4)	19.6 (16.7-22.4)	20.9 (17.1-24.7)	24.7 (20.7-28.7)	21.3 (18.0-24.5)
Natal	10.8 (8.7-12.8)	13.0 (10.6-15.3)	13.4 (10.8-15.9)	14.3 (11.9-16.7)	17.6 (14.8-20.3)	15.7 (13.2-18.2)	21.2 (17.9-24.5)	14.1 (11.8-16.5)	19.8 (16.4-23.2)	18.1 (15.2-21.1)	19.8 (16.4-23.2)
Palmas	12.2 (9.3-15.1)	12.2 (9.6-14.7)	11.3 (8.7-13.9)	9.6 (7.1-12.1)	13.4 (10.6-16.2)	14.8 (11.7-17.9)	17.5 (14.2-20.7)	14.8 (11.6-18.0)	16.3 (13.1-19.5)	14.4 (11.6-17.3)	15.3 (12.6-18.0)
Porto Alegre	12.2 (10.0-14.3)	12.3 (10.0-14.7)	13.9 (11.5-16.2)	16.3 (13.6-19.1)	14.2 (11.8-16.6)	18.8 (16.0-21.5)	18.3 (15.1-21.4)	15.0 (12.2-17.8)	18.1 (14.3-22.0)	18.7 (15.3-22.1)	18.0 (14.6-21.3)
Porto Velho	14.8 (12.3-17.4)	17.4 (14.6-20.3)	16.0 (13.4-18.6)	18.7 (15.9-21.6)	18.4 (15.4-21.3)	17.3 (14.7-20.0)	19.8 (16.6-22.9)	17.1 (13.9-20.3)	19.3 (15.8-22.8)	23.3 (19.5-27.1)	20.9 (17.4-24.4)
Recife	14.3 (11.8-16.7)	12.9 (10.4-15.3)	12.8 (10.5-15.0)	13.4 (11.1-15.8)	16.9 (14.2-19.5)	16.0 (13.6-18.4)	17.1 (14.2-20.1)	18.1 (15.5-20.7)	20.4 (17.2-23.7)	20.1 (17.3-22.9)	19.3 (16.2-22.4)
Rio Branco	14.6 (12.1-17.1)	14.1 (11.6-16.7)	18.9 (15.5-22.3)	16.6 (13.9-19.4)	19.5 (16.3-22.6)	19.8 (17.0-22.5)	24.3 (20.6-28.0)	20.2 (16.8-23.5)	18.1 (14.6-21.6)	21.9 (18.6-25.2)	23.4 (20.4-26.3)
Rio de Janeiro	12.8 (10.8-14.8)	13.5 (11.1-15.8)	13.9 (11.6-16.2)	15.2 (12.8-17.7)	17.2 (14.6-19.7)	16.2 (13.8-18.7)	20.0 (16.8-23.2)	17.9 (15.3-20.5)	17.6 (14.4-20.7)	21.8 (17.8-25.8)	20.6 (16.9-24.2)
Salvador	13.2 (10.9-15.5)	15.2 (12.8-17.6)	15.2 (12.9-17.5)	16.9 (14.4-19.3)	15.4 (13.1-17.7)	16.1 (13.7-18.6)	17.7 (14.9-20.4)	15.5 (13.2-17.8)	19.1 (16.1-22.0)	17.1 (14.5-19.8)	21.1 (17.9-24.2)
São Luís	10.3 (7.9-12.7)	11.0 (8.5-13.4)	11.3 (9.1-13.4)	12.8 (10.3-15.3)	13.1 (10.8-15.4)	14.3 (11.7-16.8)	12.7 (10.2-15.1)	14.1 (11.6-16.6)	17.5 (14.3-20.8)	14.7 (12.4-17.1)	18.3 (14.8-21.7)
São Paulo	12.0 (9.9-14.1)	12.1 (10.1-14.2)	12.7 (10.6-14.7)	13.8 (11.7-16.0)	14.9 (12.8-17.0)	15.8 (13.6-18.1)	17.1 (14.4-19.7)	16.9 (14.7-19.2)	15.8 (13.2-18.5)	20.6 (17.5-23.6)	19.4 (16.8-22.1)
Teresina	10.6 (8.5-12.7)	12.7 (10.2-15.2)	11.5 (9.2-13.7)	13.1 (10.5-15.6)	12.4 (10.0-14.8)	13.9 (11.2-16.7)	14.3 (11.5-17.0)	14.9 (12.1-17.8)	14.7 (11.4-18.1)	15.3 (12.6-18.0)	16.7 (13.5-19.9)
Vitória	9.4 (7.6-11.1)	10.4 (8.6-12.3)	11.7 (9.6-13.8)	13.2 (10.9-15.4)	14.1 (11.6-16.6)	15.3 (12.9-17.7)	13.5 (11.0-15.9)	15.9 (13.2-18.6)	15.5 (12.8-18.2)	16.8 (13.8-19.8)	16.5 (13.6-19.3)
State capitals overall	12.1 (11.4-12.8)	12.8 (12.1-13.5)	13.6 (12.9-14.3)	14.3 (13.6-15.1)	15.2 (14.5-16.0)	16.0 (15.2-16.7)	17.4 (16.6-18.3)	16.6 (15.9-17.4)	17.4 (16.5-18.3)	18.9 (17.9-19.9)	18.8 (17.8-19.7)
